# Genetic Polymorphisms Involved in Bladder Cancer: A Global Review

**DOI:** 10.3389/or.2023.10603

**Published:** 2023-11-06

**Authors:** Hampig Raphael Kourie, Joseph Zouein, Bahaa Succar, Avedis Mardirossian, Nizar Ahmadieh, Eliane Chouery, Cybel Mehawej, Nadine Jalkh, Joseph kattan, Elie Nemr

**Affiliations:** ^1^ Hematology-Oncology Department, Faculty of Medicine, Saint Joseph University, Beirut, Lebanon; ^2^ Department of Human Genetics, Gilbert and Rose-Marie Chagoury School of Medicine, Lebanese American University, Beirut, Lebanon; ^3^ Medical Genetics Unit, Faculty of Medicine, Saint Joseph University, Beirut, Lebanon; ^4^ Urology Department, Faculty of Medicine, Saint Joseph University, Beirut, Lebanon

**Keywords:** bladder cancer, genetic polymorphism, SNP, risk factor, gene

## Abstract

Bladder cancer (BC) has been associated with genetic susceptibility. Single peptide polymorphisms (SNPs) can modulate BC susceptibility. A literature search was performed covering the period between January 2000 and October 2020. Overall, 334 articles were selected, reporting 455 SNPs located in 244 genes. The selected 455 SNPs were further investigated. All SNPs that were associated with smoking and environmental exposure were excluded from this study. A total of 197 genes and 343 SNPs were found to be associated with BC, among which 177 genes and 291 SNPs had congruent results across all available studies. These genes and SNPs were classified into eight different categories according to their function.

## Introduction

Bladder cancer (BC), one of the most common cancers worldwide, is particularly prevalent in developed countries [1, 2]. Its global incidence was estimated to be equal to 3%, in 2020, according to GLOBOCAN [1, 2]. It is a complex disease that involves different risk factors, mainly smoking and occupational carcinogen exposure [3], in addition to genetic susceptibility [4]. In fact, the lifetime absolute risk (AR) of developing BC in 50 years-old white non-Hispanic never-smoker men is 1.9%, whereas the AR is 7.1% for current smokers among 50 years-old white non-Hispanic men [5]. Recent advances in DNA sequencing technologies, in particular next-generation sequencing approaches, have opened new horizons, allowing a better understanding of genetic triggers related to BC [6]. Several genomic alterations were found to be linked to this specific cancer, including gene rearrangements, amplifications, deletions, copy number variations, and point variants, including pathogenic and polymorphic variants that are also known as single nucleotide polymorphisms (SNPs) [7]. SNPs, which account for 90% of the human genome’s variability, are gaining much interest in the oncogenetics field since many of them have been shown to modulate cancer susceptibility by increasing or decreasing the risk of an individual developing cancer [8]. Among genes associated with cancer, we can cite genes regulating environmental carcinogen metabolism, DNA repair, or cell cycle pathways, all of which are involved in the development and/or progression of any type of cancer, including BC [9, 10].

Many studies have examined the association of SNPs with BC in different populations. This extensive literature review aims to list all SNPs that are significantly associated with BC and discuss their involvement in this type of cancer.

## Methods

**FIGURE 1:**
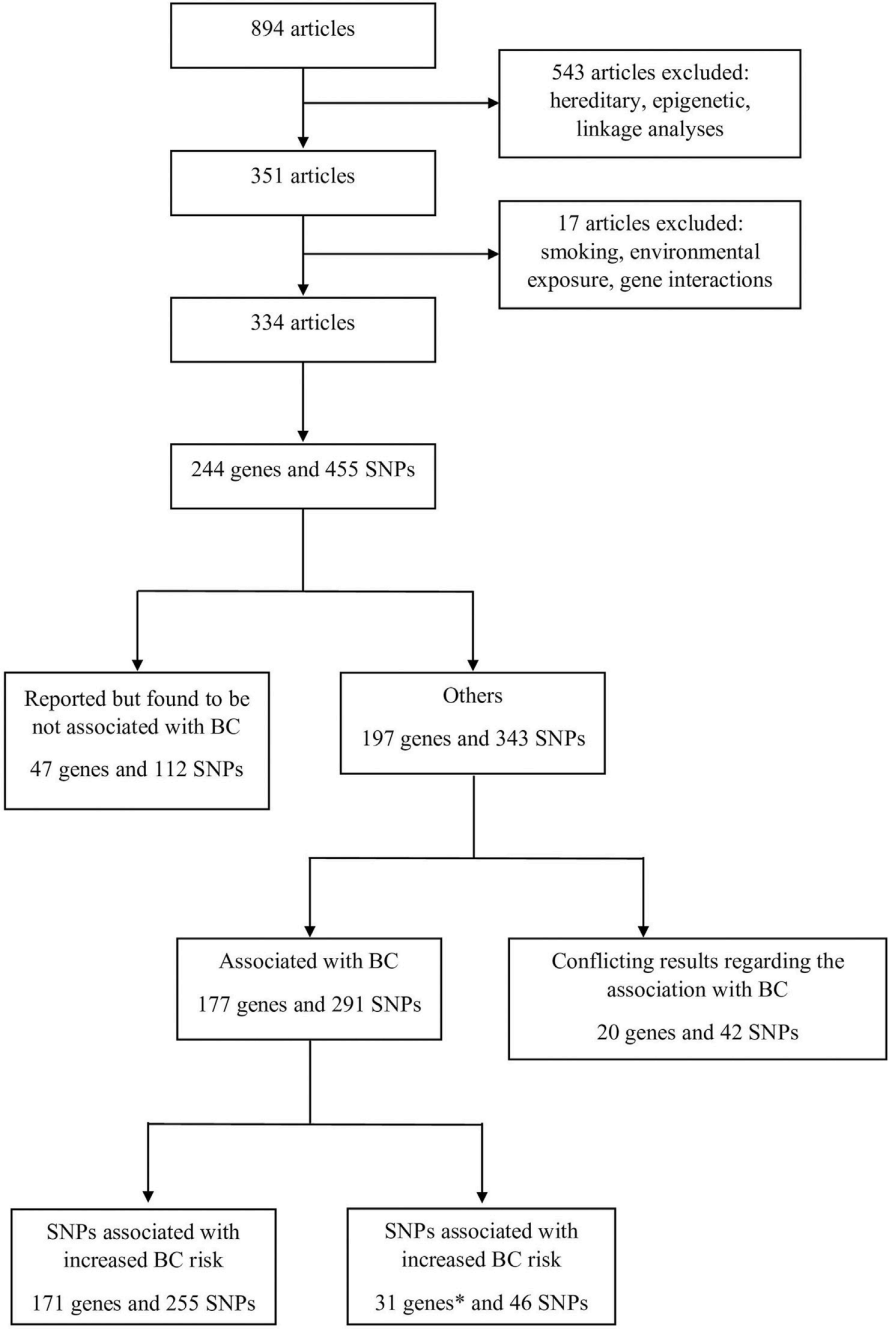
Process of the selection of the articles that were included in this study.* Genes with some SNPs that increase BC risk and others that decrease BC risk were counted twice.

In order to gather all available information related to the possible association of various SNPs with BC, an extensive literature search was performed in the PubMed database covering the period between January 2000 and October 2020. The keywords “*bladder cancer*” combined with “*genetic predisposition*” were used with Boolean operators. Overall, 894 articles were selected. The titles and abstracts of these articles were assessed for eligibility before evaluating their contents. Articles that target hereditary cancer, epigenetic studies, and linkage analysis studies were not included. At the end, 334 articles were selected, reporting 455 SNPs located in 244 genes. Selected genes were then classified based on the same method used by [11]. The article selection process is summarized in Figure 1 as a PRISMA flow diagram.

The selected 455 SNPs were further investigated. All SNPs that were associated with smoking and environmental exposure but did not present a direct association with BC were excluded from this study. However, SNPs associated with BC independently of interfering factors like smoking status, sex, age, and others were included.

## Results

In total, 197 genes and 343 SNPs were found to be associated with BC, of which 177 genes and 291 SNPs had congruent results between all available studies, meaning that all studies that assessed a particular SNP had the same results regarding the SNP association with BC. The remaining 20 genes and 42 SNPs were reported with conflicting data, thus representing a huge amount of scattered data that needs a clear classification in order to facilitate their accessibility to researchers. These genes and SNPs were thus thoroughly evaluated and classified into eight different categories according to their function: chemical carcinogenesis, signaling, cell death, DNA repair, cell cycle, cell architecture, metabolic, and other genes that do not correspond to any specific category. Further subclassifications were also enclosed in each category (Table 1).

**TABLE 1:** Summary of genes classification with number of genes in each category.

	Congruent data		Conflicting results	
	Genes	SNPs	Genes	SNPs
Chemical carcinogenesis	13	38	18	53
Signaling	34	54	39	63
Cell death	13	15	14	16
DNA repair	23	32	27	47
Cell cycle	9	16	10	18
Cell architecture	13	14	14	16
Metabolic	20	27	23	34
Others	52	95	52	96
Total	**177**	**291**	**197**	**343**

Genes and SNPs that showed concordant results between all available studies are designated “associated”. Under the category “conflicting results” are classified genes and SNPs that showed discordant results between the available studies.

Bold values represent the total number of genes and SNPs in each category.

### Chemical Carcinogenesis

Genetics and environmental factors impact the quantities of enzymes that participate in the activation and detoxification of chemicals, which can potentially lead to carcinogenesis. Most chemical compounds become carcinogens after being metabolized to chemically reactive electrophiles, which can interact with DNA to generate a carcinogenic response [12]. Genes encoding enzymes modulating chemical carcinogenesis were found to be linked to BC. In this section, genes were divided according to the different known enzyme families. Genes that encode for phase I and phase II enzymes were subcategorized into four categories: Cytochromes, Glutathione S-Transferase, N-acetyltransferase, and UDP-Glucuronosyltransferase (UGT).

Cytochromes are phase I enzymes whose role is to convert xenobiotics into excretable compounds. They are divided into subfamilies and are primarily located in the liver, but can also be found in the gastrointestinal tract, lungs, and kidneys. Mutations in these genes can affect substrate transformation, which may lead, in some cases, to cancer [13]. For instance, rs2472299 in *CYP1A* is known worldwide as a risk factor for BC [13], while rs4646903 and rs2198843 in the same gene [13, 14] are known to widely increase the risk for the same type of cancer but exclusively in the Asian population, and rs4646421 is specific for BC in Tunisians [15]. Results regarding rs1048943 in *CYP1A*1 are contradictory, since it increases the risk for BC in Asian and Brazilian populations but was not associated with BC in many other studies [13, 16–19]. Similarly, rs762551in *CYP1A2* showed conflicting results [20–22]. Regarding the *CYP1B1* gene: rs2855658 increases BC risks in the European population [23], and rs10012, rs1056827, and rs150799650 showed an increased risk in Indo-European cohorts [24], while rs1056827 was associated with a decreased risk in the Spanish population [25]. Contradictory results were seen for rs1056836, which presents a notable increased risk for BC in the Spanish population [13, 19, 25, 26]. On the other hand, rs4244285 and rs4986893 in *CYP2C19* showed a protective effect against BC in the Chinese population [27]. *CYP2E1* was associated with an increased risk for BC in the Lebanese population [28]. However, rs2031920 in the same gene was widely associated with a decreased risk in Asians [29] with controversial results in Caucasians [29, 30].

Glutathione S-Transferase constitutes a superfamily of phase II multipurpose enzymes that contribute to metabolic detoxification processes that protect macromolecules from environmental carcinogens, reactive oxygen species, and chemotherapeutic agents. The glutathione S-transferase family includes the following enzymes: GSTA, GSTM, GSTP, GSTT, and GSTO. Since these molecules contribute to the metabolism of potential carcinogens, any polymorphism affecting their expression or function may lead to cancer [31]. Most articles show that in most populations, the *GSTM1* null genotype [32–47] and rs1695 in *GSTP1* [16, 19, 33, 40, 42, 48–55] increase the risk for BC. However, a Chinese study showed that the *GSTT1* null genotype was associated with a decreased risk for BC [36]. Rs156697 in *GSTO2* also shows a positive association with BC in the Serbian population [56]. The polymorphisms in *GSTT1* were mostly associated with an increased risk for BC [16, 18, 32–34, 40, 42, 44, 47, 48, 57–63]. Rs3957356, rs3957357, and rs4715332 in *GSTA1* were associated with an increased risk for BC in both Balkan-Ben and Balkan-non Ben [63].

N-acetyltransferase (NAT) enzymes, also known as NAT1 and NAT2, are responsible for acetylating aromatic and heterocyclic amines in the liver, the gastrointestinal tract, and the urinary bladder. Genetic polymorphisms in these enzymes can alter the processing rate of various carcinogenic compounds, thus increasing the risk for cancer. Hence, slow NAT2 acetylation is associated with increased risk for BC [64]. Many SNPs in *NAT1* are associated with a significantly increased risk for BC in the Lebanese population, including rs15561, rs4986782, and rs1057126 [28, 65, 66]. However, the latter did not show any association with BC in two other meta-analyses [4, 67]. Rs9650592 in *NAT1* was positively associated with BC in the European population [23]. A study conducted on a French Caucasian population demonstrated an increased risk for BC in the presence of rs1208, rs1801280, or rs1041983 in *NAT2* [19]. Rs1041983 presents the same effect in the American population [40]. Rs1799929, rs1799930, and rs1799931 were shown to be associated with BC in French and Bangladeshi populations [19, 68]. However, a Chinese meta-analysis contradicted the French results and found no association between these SNPs and BC [69]. Finally, rs1495741 and rs4646249 increase the risk of BC [11, 37, 70] in the European population [23].

The UDP-Glucuronosyltransferase (UGT) family of enzymes are phase II enzymes that are involved in the glucuronidation of aromatic amines and other carcinogens. They are primarily located in the liver but also in the gastrointestinal tract, lungs, and kidneys. The UGT family is composed of UGT1A, UGT2A, and UGT2B. They present different subtypes that are all, except for UGT2B17, expressed in normal bladder tissue [71]. Conflicting data was documented regarding the association of rs11892031 in *UGT1A10/UGT1A8* with BC [23, 39, 70, 72–75]. Rs17863783 in *UGT1A6* decreases the risk of developing BC [70, 76]. Rs1104892, rs1105880, rs1113193, rs1604144, rs17854828, rs17864684, rs17868322, rs2602374, rs2741042, rs2741044, rs2741045, and rs4148326 in *UGT1A8* are associated with an increased risk in the American population, while rs1113193, rs1604144, rs17854828, rs17864684, rs4148328, rs4233633, and rs7571337 are associated with a decreased risk in the same population [77]. Rs2736520 in *UGT2B4* increases the risk for BC, while Rs3822179 decreases the risk in the American population [77].

CDC-like kinase 3 (CLK3) is a dual specificity kinase that belongs to the serine/threonine type kinase family. Its role in human cancer is still undetermined [78]. However, a Japanese study has established that rs11543198 is associated with an increased risk for BC in the Japanese population [79].

### Signaling

Signaling and immune-related genes were found to be associated with BC in the literature. These genes were divided into four categories, including cytokines, toll-like receptors, transcriptional factors, and other genes.

For an effective immune response against cancer cells, a sequence of steps must take place in fighting tumor cells. Each step of this sequence requires the presence of several stimulatory and inhibitory factors [80]. Among these actors, Cluster of Differentiation (CD) 274 [also known as Programmed Death Ligand 1 (PD-L1)], has an inhibitory function toward immune response activation. Rs4143815 in this gene increases the risk for BC in the Chinese population [81]. Cytotoxic T-lymphocyte-associated protein 4 (CTLA4) can also inhibit T cell-mediated immune responses, and rs231775 and rs3087243 in the corresponding gene have been shown to increase the risk for BC in the Indian population [82]. Intercellular Adhesion Molecule 1 (ICAM1) is responsible for T cell tumor infiltration, and therefore a variation in this gene can increase cancer susceptibility, as shown for rs5498 in the Taiwanese population [83]. Furthermore, chemokines help regulate the immune response and impact cancer progression [84]. Among the genes encoding chemokines, C-C chemokine receptor type 2 (*CCR2*) rs1799864 [85–87] and *CCR5* rs333 [85] were associated with an increased risk for BC in the Turkish population, rs1801157 in C-X-C motif chemokine 12 (*CXCL12*) also has a positive association with BC in Indian [88] and Turkish [85] populations; and rs1126579 in CXC chemokine receptors 2 (*CXCR2*) increases cancer’s susceptibility in the Indian population [88] while it shows a protective effect towards BC in the American population [89]. Finally, rs187115 in *CD44* was associated with an increased risk for BC in the Taiwanese population [90]. Tumor-secreted T-helper 17 (Th17) cell-associated interleukins (IL) mobilize immune suppressive cells and promote tumor growth [91]. Rs2275913 in *IL-17A* and rs187238 in *IL-18* increase the risk for BC in the Polish population, with the highest association for IL-18 [92]. Rs1946518 in *IL-18* also increases the risk of BC in the Indian population [93]. Rs2227485 in *IL-22* is also a risk factor for BC in the Chinese population [94]. Other interleukins that play a role in macrophages and neutrophils’ immunosuppressive roles include IL-10, IL-12, and IL-23 [95]. Rs1800896, rs1800871, and rs1800872 in *IL-10* [96] are associated with an increased risk for BC in Chinese [97] and Indian [98] populations; rs10889677 in *IL-23R* also increases BC risk in Chinese [99] and Polish [92] populations; and *IL-12* decreases the risk in the Indian population [93]. Other interleukins were also found to play a role in BC risk; for instance, rs2069762 in the *IL-2* gene increases the risk for BC in the Chinese population. Rs4073 in *IL-8/CXCL8* gives high BC susceptibility in the Indian population [100] and rs1800890 in *IL-19* in the Spanish population [101]. Last but not least, rs153109 and rs17855750 in *IL-27* predispose to BC in Spanish and Chinese individuals, respectively [102, 103]. Finally, rs1799964 in *TNF-*α [104] and rs1800470 in *TGFB1* [105] are positively associated with BC in the Indian population.

Toll-like receptors (TLR) play a key role in the initiation of the innate immune response. They are expressed on both immune and tumor cells and regulate the immune responses in tumor progression and as therapeutic targets for cancer [106]. *TLR2* mutations (−196 to −174del) increase the risk of BC in India [107]. Rs11536889 in *TLR4* [108–110] and rs72552316 in *TLR7* [111] constitute separate BC risk factors.

Transcription factors might be linked to BC [112]. For instance, rs2228570 in *VDR* (Vitamin D receptor) was found to increase the risk of developing BC in India [113] and Tunisia [114]. *TP63* can act as a tumor suppressor gene or as an oncogene depending on the cellular setting and pathways where it is implicated, and it can thus regulate the transcription of different genes [115]. Several SNPs have been shown to increase the risk of BC, among them rs4687100 [116] and rs710521 [39, 70, 116]. Of note, the latter has also been proven to present a protective effect in the Indian population [117], but no association with BC was identified in a Chinese study [118]. Finally, conflicting data were also obtained for rs35592567 since it was shown to have a positive association in one meta-analysis [11] and a negative association in another [119].

Vascular endothelial growth factor (VEGF) regulates angiogenesis and is upregulated and overexpressed in BC [120]. Rs699947 and Rs35569394 in *VEGF* increase and decrease, respectively, the BC risk in the Indian population [121]. Moreover, rs833052, rs25648 [122], rs3025039 [122, 123], and rs699947 [124] in *VEGFA* showed a positive association with BC, while the latter also presented a protective effect in the Tunisian population [125]. Rs3775194, rs1485762, rs6828869, and rs17697515 in *VEGFC,* and rs4557213 in *VEGFR* increase the risk for BC in the American Caucasian population [126] while rs1485766 in *VEGFC* increases the risk in the Taiwanese population [127]. Human Leukocyte Antigen G (HLA-G) expression in tumors promotes the immune suppressive microenvironment, which results in poor treatment response and prognosis [128]. Rs1063320, rs1610696, rs1704, rs1707, rs1710, rs17179101, and rs17179108 in *HLA-G* are associated with increased cancer susceptibility in the Brazilian population [129].

Other SNPs that were also studied in various genes include: rs6593205 and rs7799627 in Epidermal Growth Factor Receptor (*EGFR*), which decrease the risk for BC in the American Caucasian population, while rs11238349 increases the risk in the same population [126] and rs1050171 increases the risk in the Chinese population [130]. Rs696 in *NFKBIA* and Rs11188660 in *BLNK* (B cell linker) increase the risk in the Spanish population [101]. Rs28362491 in *NFKB1* [131–133] and rs7944701 in *MAML2* [134] increase the risk in the Chinese population. A more extensive list is available in Supplementary Table S1 under the section titled “signaling.”

### Cell Death

By evading death, immortal cells can develop into tumors. Mutations in certain pathways promoting cell death can thus lead to tumor formation. Cell death pathways involve multiple genes, including cell-death receptors such as Tumor Necrosis Factor Receptor 1 (*TNFR1*), *FAS*, and TNF-related apoptosis-inducing ligand (*TRAIL*) receptors and effectors such as caspases [135]. Genes and SNPs related to those pathways were reported in BC. Rs2234767 in *FAS*, Rs763110 in *FASLG* [136], and Rs1131580 in *TNFSF10* [137] increase the risk for BC in the Turkish population. In *DR4* (*TRAILR1* or *TNFRSF10A*), rs6557634 increases the risk for BC the Indian population [138] and rs13278062 increases the risk in the Chinese population [139] while rs20575 was found to present a protective effect in the Caucasian population [140]. Moreover, rs2647396 in *BCL10* [101], rs10999426 in *PRF1* [101], and rs42490 in *RIPK2* [141] each individually increase the risk for BC in the Spanish population, while rs401681 in *CLPTM1L* increases the risk widely in all studied populations [39, 73, 74]. Rs4647603 in *CASP3* and rs3181320 and rs507879 in *CASP5* increase the risk for BC in Indians [138] while rs3834129 in *CASP8* [142] and rs4645978 in *CASP9* [143] decrease the risk for BC in the Chinese and Indian populations, respectively. Finally, rs1045411 in *HMGB1* presents a protective effect in the Chinese population [144].

### DNA Repair

The DNA repair genes associated with BC are classified according to the repair mechanism: base excision repair (BER), nucleotide excision repair (NER), homologous recombination (HR), and poly-ADP-ribosylation.

BER is a mechanism of DNA reparation that is not restricted to the repair of single-strand breaks but also of damages resulting from defects in alkylation, oxidation, deamination, and depurination. Given that this mechanism repairs thousands of errors per cell and per day, it plays a major role in cancer prevention by ensuring genome integrity and stability. Therefore, BER guarantees the integrality of apoptosis pathways and prevents mutation accumulation that may initiate tumors [145]. In X-ray repair cross-complementing protein 1 (*XRCC1*), rs1799782 and rs25489 are widely associated with an increased risk for BC, especially in Asian and Indian populations [146–152]. Rs915927 in the same gene increases tumor susceptibility in the Italian population [153] while rs25487 gave contradictory results in different studies [147–149, 154–159]. Rs3218373, rs3218536, and rs6464268 in *XRCC2* present a protective effect towards BC in the Italian population [160]. Rs1805377 in *XRCC4* increases the risk of BC in Spanish [160] and Indian individuals [161]. Rs6869366 increases the risk of BC in Taiwanese people [162] but decreases the risk in the Indian population [161]. Rs828907 in *XRCC5* increases BC risk in the Taiwanese population [163]. Rs3213245 and rs7003908 in *XRCC7* (*PRKDC*) are positively associated with BC, respectively, in the Chinese Han [164] and in the Indians [165]. Other SNPs in BC-associated BER genes include rs1760944 in *APEX1*, which decreases the risk for BC in the Chinese population [146] while rs1130409 in *APE1* showed conflicting data [147, 166, 167]. Rs3136717 in *POLB* [168] and rs1052133 in *hOGG1* [147, 165, 169–171] are associated with a high risk for BC, while rs125701 in *hOGG1* decreases the risk for BC in the Spanish population [168]. Finally, rs2029167 in *SMUG1* and rs3219487 in *MUTYH* increase the risk for BC in the American population [172] and rs11039130 in *DDB2* has the same effect in the Caucasian population [173].

NER is also an important DNA repair pathway consisting of two distinct sub-pathways. The global-genome NER process fixes helix damages over the entire genome, whereas the transcription-coupled NER mechanism acts during transcription to resolve RNA polymerase blocking lesions [174]. Mutations in genes implicated in NER can result in a predisposition to cancer since mutations and chromosomal abnormalities can either activate oncogenes or inactivate tumor suppressor genes [175]. Rs3212961 in *ERCC1* increases BC risk in the Spanish population [176] whereas rs967591, rs735482, and rs2336219 have a protective effect towards BC in the Italian population [153]. Rs13181 [15, 157, 177–182], rs1799793 [166, 170, 177, 178, 183–186] and rs238406 [176, 178, 179] in *ERCC2* (*XPD*) were individually widely associated with an increased BC risk. Rs1047769 [176] and Rs17655 [187] in *ERCC5* increase the risk of BC, respectively, in Spanish and Chinese people. Rs2228526 and rs2228528 in *ERCC6* increase BC susceptibility in Belarussians [181] with the latter also having the same effect in the Taiwanese population [188]. Rs4150667 in *GTF2H1* is associated with an increased risk for BC in the Caucasian race [173], rs1805335 in *RAD23B* increases the risk in the Spanish population [176] and rs2228000 [189–193] and rs2228001 [155, 187, 193–195] in *XPC* are positively associated with BC in all study populations.

HR is another mechanism that helps maintain the genome’s integrity by repairing double-strand breaks. Therefore, HR deficiencies that result from gene mutations make individuals more susceptible to cancer [196]. Rs861539 in *XRCC3* is associated with an increased risk for BC in different populations [160, 197–199]. Rs1799794 and rs861530 in the same gene also increase BC risk in the Chinese population [200]. Rs11571833 in *BRCA2* increases BC risk in those of European descents [201]. Rs1805794 in *NBN* is associated with a high risk for BC [202] while rs8032440 in *FANCI* and rs3739177 in *PNKP* make the American population more susceptible to BC [172].

Poly-ADP-ribosylation is a post-translational modification catalyzed by poly(ADP-ribosyl)ation polymerases (PARPs) as a response to DNA damage [203]. Rs1136410 in *PARP1* is associated with a high BC risk in the Spanish population [168], whereas rs3219123 and rs12568297 in *PARP1*, rs1713413 in *PARP2*, and rs2862907 in *PARP4* increase separately the risk for BC in the American population [172].

### Cell Cycle

Genes and their corresponding SNPs related to the cellular cycle were separated into three groups consisting of tumor suppressor genes, inhibitors of apoptosis, and other genes associated with BC.

Tumor suppressor genes work by repairing DNA damage, inhibiting cell division, and, in some cases, triggering apoptosis to stop tumor development. Therefore, inactivation or loss of function resulting from mutations in these genes can lead to cancer [204]. An SNP in intron 3 of the *TP53* gene was associated with a decreased risk for BC in the American population [183]. Moreover, rs1042522 in the same gene is associated with an increased risk for BC in Asia [205–207]. However, this same SNP showed a protective effect towards BC in the Indian [208] and Brazilian [209] populations, and rs17878362 has the same effect in the American population [210]. Rs2839698 in *H19* shows a protective effect towards BC in the Netherlands [211] whereas rs217727 in *H19* [212, 213] and rs760805 in *RUNX3* [214] increase BC risk in the Chinese population. Finally, rs2073636 in *TSC2* is associated with a high risk for BC in the American population [172] and rs17879961 in *CHEK2* with a low risk for BC in European descent [201].

Inhibitors of apoptosis are a family of eight proteins. A mutation activating any of the corresponding genes will become a weapon that will be used by tumors to evade apoptosis [215]. Rs2071214 [216], rs8073069 [216], rs9904341 [216–219], and rs3764383 [219] in *BIRC5* increase BC risk, while rs17878467 decreases the risk in Asians [216, 219, 220].

Other genes include Cyclin D1 (*CCND1*) which regulates cell cycle progression through the activation of cyclin-dependent kinases 4 and 6 (CDK4 and CDK6) that lead to Rb protein phosphorylation, thus inactivating it and allowing the cell to progress past the G1/S checkpoint and continue its replication. Over-expression of *CCND1* can lead to cancer [221] and the rs9344 in this gene increases the risk for BC [221–225]. Similarly, Cyclin E1 (*CCNE1*) binds to CDK2 and activates it, allowing the cell to progress and enter phase S [226]. Rs8102137 in *CCNE1* widely increases BC development risk [11, 39, 73, 75, 116]. Moreover, the PI3K–AKT–mTOR is implicated in cell growth, tumorigenesis, and cell invasion [227]. Mitochondrial gene *POLG* polymorphism rs3087374 increases BC risk in the American population [172]. Rs2294008 [11, 37, 39, 73, 79, 228–235] and rs2978974 [70] in the prostate stem cell antigen (*PSCA*), an inhibitor of cell proliferation, increase widely BC risk.

### Cell Architecture

Caveolin‐1 (*CAV1*) is an essential membrane protein expressed in multiple cells. It plays a central role in the formation of caveolae, which are small plasma membrane invaginations involved in signaling and transport. The role of CAV1 in carcinogenesis has been proven. However, the mechanism is still unknown [236]. Rs3807987 and rs7804372 in *CAV1* increase the risk for BC in the Taiwanese population [237] while rs1049334 widely increases it. CLTA and CLTC, which encode the light and heavy chains of clathrin, are also associated with BC. Indeed, rs10972786 in *CLTA* and rs7224631 in *CLTC* increase the risk of BC in the European population [23]. Clathrin-mediated vesicle pathways include the *DNM1* and *DNM2* genes. Rs13285411 in *DNM1* and rs4804528 in *DNM2* increase the risk for BC, while rs4804149 in *KANK2* has a significant protective effect towards BC in the European Population [23]. Finally, rs12216499 in *RSPH3* widely increases the risk for BC [70], while rs907611 in *LSP1* increases the risk for BC in the European population [238] but was found not to be associated with BC in Chinese [70].

Rs738141 in Dynein light chain 4 (*DNAL4*) implicated in cell motricity increases the risk for BC in the European population [23].

Rs16260 in Cadherin-1 (*CDH1*) contributes to BC susceptibility in the Chinese population [239].

The extracellular degradation process is mediated by the Matrix Metalloprotease (MMP), which plays a regulatory role in multiple pathways such as apoptosis or angiogenesis. Hence, MMPs participate in carcinogenesis [240]. Conflicting results concerning the association of rs1799750 in *MMP1* with BC were obtained [241–245]. Rs243865 in *MMP2* has shown an increased BC risk in India [246] and in a meta-analysis conducted by [244]. However, another meta-analysis done by L. Tao et al. has shown, for the same SNP, a protective effect towards BC in the Asian population [245]. Contradictory results have also been reported for rs11568818 in *MMP7* [242, 245, 247]. Finally, rs28382575 in *MMP11* increases BC risk in Taiwan [248].

### Metabolic

Metabolism-related genes were found to be associated with BC. These genes were divided into alcohol metabolism genes, solute carrier transporters (SLC), folate metabolism enzymes, water-soluble vitamin metabolism genes, and other various metabolism-related genes.

Alcohol metabolism requires multiple enzymes. Rs12529 in *AKR1C3* decreases BC risk in the Turkish population [249] while Rs4680 in *COMT* has shown contradictory results [19, 250, 251].

SLC transporters are membrane proteins whose role is to supply cells with nutrients, neurotransmitters, hormones, and drugs. They are usually upregulated in cancer [252]. Rs17674580 in *SLC14A* [39, 253] and rs1058396 [74], rs10775480 [11, 254], rs10853535 [70, 254], rs17674580 [37, 79, 117], and rs7238033 [39, 70, 254] in *SLC14A1* increase BC susceptibility in different populations. Rs1385129 in *SLC2A1,* also known as Glucose transporter 1 (*GLUT1*), decreases the carcinogenesis risk in the Chinese population [255]. Rs11871756, rs11077654, rs9913017, and rs4969054 in *SLC39A11* increase the risk for BC in different populations [256] and rs2306283 in *SLCO1B1* increases the risk in the Japanese population [257].

Mitochondrial folate metabolic enzymes are associated with cancer [258]. Hence, rs1667627 in *MTHFD2* increases the risk of developing BC in the American population [89]. Other SNPS in genes involved in the folate metabolism are: rs1801131 [259–262] and rs1801133 [260–265] in *MTHFR* that are widely classified as risk factors for BC, whereas rs1476413 in the same gene decreases the risk of cancer occurrence [266], rs1805087 in *MTR* increases the risk for BC in the Tunisian population [262, 267] and rs1801394 in *MTRR* increases the risk for BC only in Saudi Arabia [18].

SNPs in the metabolism of water-soluble vitamin genes that increase BC risk include rs61330082 in *NAMPT* in the Chinese population [268], rs4652795 in *NMNAT2*, rs7636269 in *NMNAT3,* and rs2304191 in *NMRK2* in the European population [23].

Other metabolism genes that are associated with BC are reported in Supplementary Table S1.

### Others

Finally, unclassified genes that present various actions and are implicated in different pathways were also found to be associated with BC. These genes are reported under the section named “Others” in Supplementary Table S1.

### Not Associated SNPs

Many other SNPs in different genes were also studied and found not to modulate BC risk. Among these, rs3892097 in *CYP2D6* was not found to be associated with BC in the Indian [62] and Tunisian [269] populations. Moreover, rs1800629 in *TNF-*α [270, 271] and rs833061 in *VEGFA* [122] also showed no association with BC in a meta-analysis. Rs4253211 in *ERCC6* [272], rs1042489 in *BIRC5* [216] and rs4880 in *SOD2* [273] were also not associated with BC. Finally, rs11225395, rs35866072, and rs1940475 in *MMP8* [274], as well as rs3918242 [243, 244, 275], rs3918241, rs2250889, rs17576, and rs17577 in *MMP9* all showed no association with any risk for carcinogenesis [276]. Other genes and SNPs that strictly showed no association with BC risk are reported in Supplementary Table S1 under the section “No association.”

## Discussion

Several SNPs associated with BC are restricted to specific populations. A generalization on an SNP’s potential role in predisposing or protecting an individual from BC cannot be done without testing this specific SNP in a sufficient number of individuals from different races and origins. Some SNPs were studied in different populations, which enabled us to correlate them to BC risk. On the other hand, many other SNPs that were exclusively found not to be associated with BC were only studied in one or two populations. Therefore, the scarcity of the gathered data related to these SNPs renders the interpretation of their correlation with BC challenging and requires broader studies for the validation of their contribution to this type of cancer. For SNPs with conflicting results between studies, GWAS-specific repositories could be useful.

On the other hand, the risk conferred by these variants cannot alone explain the development of BC [277]. In fact, several environmental factors were found to play a role in the pathogenesis of the disease. These include: gender, age, smoking habits, alcohol consumption, and potential environmental exposure. It is also worth mentioning that some SNPs were found to be associated with more invasiveness and recurrence. For example, *PSCA* rs2294008 was associated with a more invasive disease [234].

Individual SNPs effects on modulating BC risk are minimal. Therefore, studies have shifted towards assessing the polygenic risk score (PRS), as it represents a more accurate representation of an individual’s risk of developing BC. PRS aggregates the effects of multiple SNPs to provide a disease risk prediction [278]. A PRS based on 24 independent GWAS markers showed a fourfold increase in BC risk for both smokers and nonsmokers [5].

Finally, the clinical value of such information has yet to be investigated. In fact, recommendations regarding the clinical management of patients based on their genotypes are still lacking [279].

## Conclusion

SNPs are genetic variants that are generally population specific [8]. This review shows that several SNPs were associated with BC, depending on the studied cohorts. The generalization of the link between a variant and BC is not always possible, especially in the absence of data related to large cohorts from different ethnicities. For instance, several SNPs associated with BC are private to specific populations. On the other hand, the involvement of many SNPs in BC was ruled out based on studies focusing on one to two cohorts only. The scarcity of related data urges us to gather all the information we can in order to make it accessible to the scientific community. However, one should consider the complexity of the interpretation of these genomic markers [277], especially that, in many cases, the cumulative effect of several SNPs contributes to modulating the risk for BC [280], in addition to epidemiological or environmental factors such as gender, age, smoking habits, and alcohol consumption. Last but not least, the clinical value of such information has yet to be investigated.

## Data Availability

The original contributions presented in the study are included in the article/Supplementary Material, further inquiries can be directed to the corresponding author.
