# Supplementary material for: Genetic Polymorphisms Involved in Bladder Cancer: A Global Review
Source: Oncol Rev. 2023 Nov 6;17:10603. doi: 10.3389/or.2023.10603 (PMC10657888; doi:10.3389/or.2023.10603)
Supplement: Supplementary file 1 [file Table1.docx]

| aaccc | SNP | Genomic/cDNA | Protein | | Risk | | Population | | Cohort | | OR (95% CI) | | P-value | | Ref | | |
| --- | --- | --- | --- | --- | --- | --- | --- | --- | --- | --- | --- | --- | --- | --- | --- | --- | --- |
| Chemical Carcinogenesis | | | | | | | | | | | | | | | | |  |
| Cytochromes | | | | | | | | | | | | | | | | |  |
| CYP1A1 | rs4646903 | g.11229T>C/A |  | Increased | | MA / Asian | | 2120 BC() + 2061 N() | | 1.43 (1.04–1.96) | | 0.027 | | [14] | |  |  |
| CYP1A1 | rs2198843 | g.75001230C>G/T |  | Increased | | MA / Asian | | 2120 BC() + 2061 N() | | 1.24 (1.05–1.46) | | 0.009 | | [14] | |  |  |
| CYP1A1 | rs2198843 | g.75001230C>G/T |  | Increased | | MA | | BC() + N() | | 1.2 | | 0.03 | | [13] | |  |  |
| CYP1A1 | rs1048943 | c.1384A>T/G/C | p.Ile462Phe/Val/Leu | Increased | | Brazilian | | 100 BC(36) + 100 N(22) | | 1.99 (1.07–3.73) | | <0.05 | | [16] | |  |  |
| CYP1A1 | rs1048943 | c.1384A>T/G/C | p.Ile462Phe/Val/Leu | Increased | | MA / Asian | | 2120 BC() + 2061 N() | | 1.81 (1.05–3.10) | | 0.033 | | [14] | |  |  |
| CYP1A1 | rs1048943 | c.1384A>T/G/C | p.Ile462Phe/Val/Leu | NA | | French / Caucasian | | 51 BC(1) + 45 N(3) | | 0.2 (0.02-2.04) | |  | | [19] | |  |  |
| CYP1A1 | rs1048943 | c.1384A>T/G/C | p.Ile462Phe/Val/Leu | NA | | MA | | 1059 BC () +1061 N() | | 1.47 (0.70-3.07) | | 0.308 | | [17] | |  |  |
| CYP1A1 | rs1048943 | c.1384A>T/G/C | p.Ile462Phe/Val/Leu | NA | | Saudi arabia | | 52 BC(2) + 102 N(0) | | 1.4 (0.5–3.4) | | 0.5 | | [18] | |  |  |
| CYP1A1 | rs2472299 | g.75033400A>G/T |  | Increased | | MA | | BC() + N() | | 0.87 | | 0.04 | | [13] | |  |  |
| CYP1A1 | rs4646421 | c.-26-728C>T |  | Increased | | Tunisian | | 140 BC() + 200 N() | | 1.31 (1.22-1.40) | | 3.68x10^−12^ | | [15] | |  |  |
| CYP1A1 | rs4646903 | g.1759A>T |  | NA | | Saudi Arabia | | 52 BC(2) + 102 N(2) | | 0.8 (0.5–1.5) | | 0.56 | | [18] | |  |  |
| CYP1A2 | rs762551 | c.-9-154C>A |  | Increased | | MA / Caucasian | | 2415 BC() + 2208 N() | | 0.88 ( 0.78–0.99) | | 0.563 | | [20] | |  |  |
| CYP1A2 | rs762551 | c.-9-154C>A |  | Decreased | | MA | | 3013 BC (308) + 2771 N(328) | | 0.82 (0.69-0.99) | | <0.05 | | [21] | |  |  |
| CYP1A2 | rs762551 | c.-9-154C>A |  | NA | | MA | | 3430 BC() + 3242 N() | | 0.84 (0.70-1.01) | | 0.23 | | [22] | |  |  |
| CYP1B1 | rs10012 | c.142C>G | p.Arg48Gly | Increased | | Indo-European | | 258 BC(177) + 234 N(147) | | 2.34 (1.59-3.45) | | <0.0001 | | [24] | |  |  |
| CYP1B1 | rs1056827 | c.355G>T | p.Ala119Ser | Decreased | | Spanish | | 208 BC(77) + 208 N(108) | | 0.54 (0.37–0.80) | | 0.002 | | [25] | |  |  |
| CYP1B1 | rs1056827 | c.355G>T | p.Ala119Ser | Increased | | Indo-European | | 258 BC(180) + 234 N(152) | | 2.32 (1.57-3.44) | | <0.0001 | | [24] | |  |  |
| CYP1B1 | rs1056836 | c.1294G>C | p.Val432Leu | Increased | | Spanish | | 208 BC(114) + 208 N(88) | | 1.65 (1.12–2.44) | | 0.011 | | [25] | |  |  |
| CYP1B1 | rs1056836 | c.1294C>G | p.Leu432Val | Decreased | | MA | | BC() + N() | | 0.86 (0.73-1.0) | | 0.05 | | [13] | |  |  |
| CYP1B1 | rs1056836 | c.1294C>G | p.Leu432Val | NA | | MA | | 1658 BC() + 1592 N() | | 0.942 (0.853–1.041) | | 0.504 | | [26] | |  |  |
| CYP1B1 | rs1056836 | c.1294C>G | p.Leu432Val | NA | | French / Caucasian | | 51 BC(44) + 45 N(37) | | 1.47 (0.47-4.60) | |  | | [19] | |  |  |
| CYP1B1 | rs150799650 | c.1043+728C>A |  | Increased | | Indo-European | | 258 BC(115) + 234 N(70) | | 2.59 (1.78-3.77) | | <0.0001 | | [24] | |  |  |
| CYP1B1 | rs2855658 | g.11355A>G |  | Increased | | European | | 3532 BC() + 5120 N() | | 0.94 (0.88-1) | | 0.0477 | | [23] | |  |  |
| CYP2C19 | rs4244285 | c.G681A | p.Pro227= | Decreased | | Chinese | | 112 BC(5) + 372 N(52) | | 0.288 (0.112-0.740) | | <0.01 | | [27] | |  |  |
| CYP2C19 | rs4986893 | c.G636A | p.Trp212Ter | Decreased | | Chinese | | 112 BC(5) + 372 N(52) | | 0.288 (0.112-0.740) | | <0.01 | | [27] | |  |  |
| CYP2E1 | NA | 1019C>T |  | Increased | | Lebanese | | 54 BC(36) + 106 N(46) | | 3.97 (0.48-32.7) | | 0.19 | | [28] | |  |  |
| CYP2E1 | rs2031920 | g.3979C>T |  | Decreased | | MA / Caucasian | | 1044 BC () + 1044 N() | | 0.398 (0.173-0.915) | | 0.03 | | [30] | |  |  |
| CYP2E1 | rs2031920 | g.3979C>T |  | Decreased | | MA / Asian | | 1733 BC () + 1814 N() | | 0.69 (0.5-0.9) | | 0.04 | | [29] | |  |  |
| CYP2E1 | rs2031920 | g.3979C>T |  | NA | | MA / Caucasian | | 1733 BC () + 1814 N() | | 0.9 (0.69-1.19) | | 0.3 | | [29] | |  |  |
| GSTM1 |  | Null |  | Increased | | USA/non Hispanic whites | | 731 BC(387) + 740 N(335) | | 1.58 (1.25–1.99) | | <0.05 | | [40] | |  |  |
| GSTM1 |  | Null |  | Increased | | MA | | BC() + N() | | 1.5 (1.3–1.6) | |  | | [39] | |  |  |
| GSTM1 |  | Null |  | Increased | | German / Caucasian | | 146 BC(45) + 206 N(23) | | 3.54 (2.99-4.11) | | <0.05 | | [41] | |  |  |
| GSTM1 |  | Null |  | Increased | | Turkish | | 121 BC(75) +121 N(55) | | 1.94 (1.15-3.26) | | 0.01 | | [42] | |  |  |
| GSTM1 |  | Null |  | Increased | | MA | | 5029 BC(2941) + 6680 N(3274) | | 1.46 (1.35-1.57) | | <0.05 | | [43] | |  |  |
| GSTM1 |  | Null |  | Increased | | Korean | | 110 BC(65)+ 220 N(103) | | 1.30 (1.01–1.68) | | 0.037 | | [38] | |  |  |
| GSTM1 |  | Null |  | Increased | | Chinese | | 1050 BC() + 1404 N() | | 1.37 (1.16-1.62) | | 2.306 x 10^-4^ | | [37] | |  |  |
| GSTM1 |  | Active |  | Increased | | Balkan-BEN | | 201 BC(32) + 122 N(10) | | 2.90 (1.30-6.60) | | 0.01 | | [58] | |  |  |
| GSTM1 |  | Null |  | Increased | | MA | | 20239 BC() + 24393 N() | | 1.40 (1.31–1.48) | | <0.00001 | | [44] | |  |  |
| GSTM1 |  | Null |  | Increased | | Chinese | | 1646 BC(937) + 1938 N(899) | | 1.56 (1.36–1.79) | | <0.05 | | [36] | |  |  |
| GSTM1 |  | Null |  | Increased | | MA | | 1496 BC() + 1444 N() | | 1.4 (1.20-1.64) | |  | | [281] | |  |  |
| GSTM1 |  | Null |  | Increased | | Spanish | | 1150 BC(1036) + 1149 N(1025) | | 1.7 (1.4–2.0) | | <0.0001 | | [45] | |  |  |
| GSTM1 |  | Null |  | Increased | | Tunisian | | 62 BC(39) + 79 N(36) | | 2.03 (0.97-4.24) | | 0.046 | | [34] | |  |  |
| GSTM1 |  | Null |  | Increased | | MA | | BC() + N() | | 1.53 (1.28-1.84) | | <0.05 | | [46] | |  |  |
| GSTM1 |  | Null |  | Increased | | South Korean | | 232 BC(149) + 165(86) | | 1.6 (1.0-2.4) | | 0.01 | | [47] | |  |  |
| GSTM1 |  | Null |  | Increased | | MA | | 11473 BC() + 13795 N() | | 1.39 (1.28-1.51) | | <0.05 | | [33] | |  |  |
| GSTM1 |  | Null |  | Increased | | MA | | 12751 BC() + 15519 N() | | 1.36 (1.25-1.47) | | <0.01 | | [32] | |  |  |
| GSTM1 |  | Null |  | NA | | MA | |  | | 1.06 (0.88-1.27) | | 0.55 | | [11] | |  |  |
| GSTM1 |  | Null |  | NA | | North India | | 100 BC(37) + 76 N(24) | | 1.34 (0.72–2.51) | | 0.02 | | [62] | |  |  |
| GSTM1 |  | Null |  | NA | | Egypt | | 625 BC(332) + 626 N(344) | | 0.94 (0.74-1.18) | |  | | [59] | |  |  |
| GSTM1 |  | Null |  | NA | | Iranian | | 166 BC(50) + 332 N(93) | | 1.32 (0.82–2.62) | | 0.67 | | [48] | |  |  |
| GSTM1 |  | Active |  | NA | | Balkan-non BEN | | 201 BC(58) + 122 N(51) | | 1 | |  | | [58] | |  |  |
| GSTM1 |  | Null |  | NA | | Shanghai | | BC() + N() | |  | |  | | [57] | |  |  |
| GSTM1 |  | Null |  | NA | | Brazilian | | 100 BC(40) + 100 N(33) | | 1.35 (0.76–2.41) | |  | | [16] | |  |  |
| GSTO2 | rs156697 | c.424A>G/T | p.Asn142Asp/Tyr | Increased | | Serbian | | 182 BC(28) + 140 N(11) | | 2.6 (1.2–5.8) | | 0.041 | | [56] | |  |  |
| GSTP1 | rs1695 | c.313A>G | p.Ile105Val | Increased | | USA / non Hispanic whites | | 731 BC(387) + 740 N(335) | | 1.12 (0.78–1.61) | |  | | [40] | |  |  |
| GSTP1 | rs1695 | c.313A>G | p.Ile105Val | Increased | | Turkish | | 121 BC(54) +121 N(38) | | 1.75 (1.03-2.99) | | 0.034 | | [42] | |  |  |
| GSTP1 | rs1695 | c.313A>G | p.Ile105Val | Increased | | North India | | 106 BC(16) + 162 N(6) | | 7.68 (2.77-21.26) | | 0.000 | | [54] | |  |  |
| GSTP1 | rs1695 | c.313A>G | p.Ile105Val | Increased | | MA | | 4273 BC() + 5081 N() | | 1.54 (1.21-1.99) | | <0.001 | | [55] | |  |  |
| GSTP1 | rs1695 | c.313A>G | p.Ile105Val | Increased | | French / Caucasian | | 51 BC(31) + 45 N(17) | | 2.27 (0.97-5.31) | |  | | [19] | |  |  |
| GSTP1 | rs1695 | c.313A>G | p.Ile105Val | Increased | | Iranian | | 166 BC(112) + 332 N(160) | | 3.48 (1.34–5.61) | | 0.002 | | [48] | |  |  |
| GSTP1 | rs1695 | c.313A>G | p.Ile105Val | Increased | | MA | | 4428 BC() + 5457 N() | | 1.5 (1.13–2.00) | | 0.005 | | [53] | |  |  |
| GSTP1 | rs1695 | c.313A>G | p.Ile105Val | Increased | | MA | | BC() + N() | | 1.41 (1.10–1.80) | | 0.006 | | [52] | |  |  |
| GSTP1 | rs1695 | c.313A>G | p.Ile105Val | Increased | | MA* | | 7236 BC() + 8468 N() | | 1.33 (1.04-1.69) | |  | | [50] | |  |  |
| GSTP1 | rs1695 | c.313A>G | p.Ile105Val | Increased | | Chinese | | 200 BC(45) + 200 N(27) | | 1.86 (1.10-3.14) | | 0.02 | | [49] | |  |  |
| GSTP1 | rs1695 | c.313A>G | p.Ile 105Val | Increased | | India / Kashmir Males | | 162 BC(45) + 171 N(12) | | 5.09 (1.57-16.5) | | 0 | | [51] | |  |  |
| GSTP1 | rs1695 | c.313A>G | p.Ile 105Val | NA | | India / Kashmir | | 180 BC(51) + 210 N(51) | | 1.23 (0.56-2.68) | | 0.3 | | [51] | |  |  |
| GSTP1 | rs1695 | c.313A>G | p.Ile105Val | NA | | MA | | 5080 BC() + 6187 N() | | 1.07 (0.96–1.20) | | 0.062 | | [33] | |  |  |
| GSTP1 | rs1695 | c.313A>G | p.Ile105Val | NA | | Brazilian | | 100 BC(27) + 100 N(33) | | 0.75 (0.41–1.38) | |  | | [16] | |  |  |
| GSTT1 |  | Null |  | Increased | | USA / non Hispanic whites | | 731 BC(387) + 740 N(335) | | 1.24 (0.93–1.66) | |  | | [40] | |  |  |
| GSTT1 |  | Null |  | Increased | | South Korean | | 232 BC(135) + 166 N(85) | | 1.3 (0.9–2.0) | | 0.01 | | [47] | |  |  |
| GSTT1 |  | Null |  | Increased | | North India | | 100 BC(30) + 76 N(11) | | 2.53 (1.17–5.46) | | 0.02 | | [62] | |  |  |
| GSTT1 |  | Null |  | Increased | | Brazilian | | 100 BC(51) + 100 N(37) | | 1.77 (1.01–3.12) | |  | | [16] | |  |  |
| GSTT1 |  | Null |  | Increased | | MA | | 10805 BC() + 13332 N() | | 1.1502 (1.0384–1.2741) | | 0.0073 | | [61] | |  |  |
| GSTT1 |  | Null |  | Increased | | Balkan-BEN | | 201 BC(46) + 122 N(16) | | 2.10 (1.10-4.00) | | 0.027 | | [58] | |  |  |
| GSTT1 |  | Null |  | Increased | | MA | | 12369 BC() + 15333 N() | | 1.11 (1.00–1.22) | | 0 | | [33] | |  |  |
| GSTT1 |  | Null |  | Increased | | MA | | 20239 BC() + 24393 N() | | 1.11 (1.01–1.22) | | 0.03 | | [44] | |  |  |
| GSTT1 |  | Null |  | Increased | | Taiwanese | | 59 BC(32) + 81 N(51) | | 1.21 (0.53-2.73) | |  | | [60] | |  |  |
| GSTT1 |  | Null |  | Increased | | MA** | | 11817 BC() + 14805 N() | | 1.13 (1.02-1.25) | | <0.01 | | [32] | |  |  |
| GSTT1 |  | Null |  | Decreased | | Chinese | | 1646 BC(423) + 1938 N(487) | | 1.00 (0.83–1.21) | | 0.673 | | [36] | |  |  |
| GSTT1 |  | Null |  | NA | | Turkish | | 121 BC(27) +121 N(21) | | 1.27 (0.66-2.47) | | 0.62 | | [42] | |  |  |
| GSTT1 |  | Null |  | NA | | Egypt | | 625 BC(147) + 626 N(156) | | 1.09 (0.83-1.42) | |  | | [59] | |  |  |
| GSTT1 |  | Null |  | NA | | Iranian | | 166 BC(35) + 332 N(69) | | 1.18 (0.79–1.76) | | 0.74 | | [48] | |  |  |
| GSTT1 |  | Null |  | NA | | Serbian | | 201 BC(56) + 122 N(34) | | 1.00 (0.59–1.70) | | 0.998 | | [63] | |  |  |
| GSTT1 |  | Null |  | NA | | Balkan-non BEN | | 201 BC(98) + 122 N(72) | | 1 | |  | | [58] | |  |  |
| GSTT1 |  | Null |  | NA | | Saudi arabia | | 52 BC(6) + 102 N(8) | | 1.6 (0.7–3.4) | | 0.27 | | [18] | |  |  |
| GSTT1 |  | Null |  | NA | | Shanghai | | BC() + N() | |  | |  | | [57] | |  |  |
| GSTT1 |  | Null |  | NA | | Tunisian | | 62 BC(26) + 79 N(35) | | 0.907 | | 0.77 | | [34] | |  |  |
| GSTA1 | rs3957357 | g.52668687A>G/T |  | NA | | Serbian | | 201 BC(134) + 122 N(73) | | 1.34 (0.82–2.20) | | 0.215 | | [63] | |  |  |
| GSTA1 | rs3957356 | g.52668670T>A/C/G |  | Increased | | Balkan-BEN | | 201 BC(44) + 122 N(15) | | 2.60 (1.20-5.54) | | 0.015 | | [63] | |  |  |
| GSTA1 | rs3957356 | g.52668670T>A/C/G |  | Increased | | Balkan-non BEN | | 201 BC(89) + 122 N(58) | | 1.40 (0.80-3.50) | | 0.232 | | [63] | |  |  |
| GSTA1 | rs3957357 | g.52668687A>G/T |  | Increased | | Balkan-BEN | | 201 BC(44) + 122 N(15) | | 2.60 (1.20-5.54) | | 0.015 | | [63] | |  |  |
| GSTA1 | rs3957357 | g.52668687A>G/T |  | Increased | | Balkan-non BEN | | 201 BC(89) + 122 N(58) | | 1.40 (0.80-3.50) | | 0.232 | | [63] | |  |  |
| GSTA1 | rs3957357 | g.52668687A>G/T |  | NA | | MA | | 585 BC() + 702 N() | | 1.05 (0.83-1.33) | | 0.184 | | [33] | |  |  |
| GSTA1 | rs4715332 | g.52669185C>A/G/T |  | Increased | | Balkan-BEN | | 201 BC(44) + 122 N(15) | | 2.60 (1.20-5.54) | | 0.015 | | [63] | |  |  |
| GSTA1 | rs4715332 | g.52669185C>A/G/T |  | Increased | | Balkan-non BEN | | 201 BC(89) + 122 N(58) | | 1.40 (0.80-3.50) | | 0.232 | | [63] | |  |  |
| NAT1 | rs15561 | g.57681A>C/T |  | Increased | | Lebanese | | 54 BC(7) + 105 N(14) | | 7.86 (1.53–40.39) | | <0.05 | | [65] | |  |  |
| NAT1 | rs1057126 | g.57674A>C/T |  | Increased | | Lebanese | | 54 BC(7) + 105 N(14) | | 7.86 (1.53–40.39) | | <0.05 | | [65] | |  |  |
| NAT1 | rs4986782 | c.560G>A | p.Arg187Gln | Increased | | Lebanese | | 54 BC(7) + 105 N(14) | | 7.86 (1.53–40.39) | | <0.05 | | [65] | |  |  |
| NAT1 | rs4986782 | c.560G>A | p.Arg187Gln | Increased | | Lebanese | | 54 BC(29) + 106 N(12) | | 14.4 (1.016-204.9) | | 0.049 | | [28] | |  |  |
| NAT1 | rs1057126 | g.57674A>C/T |  | NA | | MA | | 4322 BC() + 4944 N() | | 0.96 (0.84-1.10) | | 0.022 | | [67] | |  |  |
| NAT1 | rs9650592 | g.58859G>A/C |  | Increased | | European | | 3532 BC() + 5120 N() | | 0.86 (0.78-0.96) | | 0.0054 | | [23] | |  |  |
| NAT1 | rs1057126 | g.57674A>C/T |  | NA | | MA | | 3311 BC() +3906 N() | | 0.96 (0.81-1.10) | |  | | [4] | |  |  |
| NAT1 | rs4986782 | c.560G>A | p.Arg187Gln | Increased | | Lebanese | | 54 BC(29) + 105 N(12) | |  | |  | | [66] | |  |  |
| NAT2 | rs1041983 | c.282C>T | p.Tyr94= | Increased | | USA / non Hispanic whites | | 731 BC(364) + 740 N(315) | | 1.11 (0.87–1.41) | |  | | [40] | |  |  |
| NAT2 | rs1041983 | c.282C>T | p.Tyr94= | Increased | | French / Caucasian | | 51 BC(25) + 45 N(13) | | 2.70 (1.10-6.61) | |  | | [19] | |  |  |
| NAT2 | rs1041983 | c.282C>T | p.Tyr94= | NA | | MA*** | | BC() + N() | |  | |  | | [69] | |  |  |
| NAT2 | rs1208 | c.803G>A | p.Arg268Lys | Increased | | French / Caucasian | | 51 BC(25) + 45 N(13) | | 2.70 (1.10-6.61) | |  | | [19] | |  |  |
| NAT2 | rs1208 | c.803G>A | p.Arg268Lys | NA | | MA*** | | BC() + N() | |  | |  | | [69] | |  |  |
| NAT2 | rs1495741 | g.18272881G>A |  | Increased | | Chinese | | 1050 BC() + 1404 N() | | 1.13 (1.01-1.27) | | 0.034 | | [37] | |  |  |
| NAT2 | rs1495741 | g.18272881G>A |  | Increased | | MA | |  | |  | |  | | [11] | |  |  |
| NAT2 | rs1495741 | g.18272881G>A |  | Increased | | MA / Chinese | | 1050 BC() + 1404 N() | | 1.13 | | 0.034 | | [70] | |  |  |
| NAT2 | rs1799929 | c.481C>T | p.Leu161= | Increased | | Bangladeshi | | 102 BC(37) + 140 N(15) | | 4.45 (2.26-8.77) | | < 0.001 | | [68] | |  |  |
| NAT2 | rs1799929 | c.481C>T | p.Leu161= | Increased | | French / Caucasian | | 51 BC(25) + 45 N(13) | | 2.70 (1.10-6.61) | |  | | [19] | |  |  |
| NAT2 | rs1799929 | c.481C>T | p.Leu161= | NA | | MA*** | | BC() + N() | |  | |  | | [69] | |  |  |
| NAT2 | rs1799930 | c.590G>A | p.Arg197Gln | Increased | | Bangladeshi | | 102 BC(37) + 140 N(15) | | 4.45 (2.26-8.77) | | <0.001 | | [68] | |  |  |
| NAT2 | rs1799930 | c.590G>A | p.Arg197Gln | Increased | | French / Caucasian | | 51 BC(25) + 45 N(13) | | 2.70 (1.10-6.61) | |  | | [19] | |  |  |
| NAT2 | rs1799930 | c.590G>A | p.Arg197Gln | NA | | MA*** | | BC() + N() | |  | |  | | [69] | |  |  |
| NAT2 | rs1799931 | c.857G>A | p.Gly286Glu | Increased | | Bangladeshi | | 102 BC(37) + 140 N(15) | | 4.45 (2.26-8.78) | | <0.001 | | [68] | |  |  |
| NAT2 | rs1799931 | c.857G>A | p.Gly286Glu | Increased | | French / Caucasian | | 51 BC(25) + 45 N(13) | | 2.70 (1.10-6.61) | |  | | [19] | |  |  |
| NAT2 | rs1799931 | c.857G>A | p.Gly286Glu | NA | | MA*** | | BC() + N() | |  | |  | | [69] | |  |  |
| NAT2 | rs1801279 | c.191G>A | p.Arg64Gln | NA | | MA*** | | BC() + N() | |  | |  | | [69] | |  |  |
| NAT2 | rs1801280 | c.341T>C | p.Ile114Thr | Increased | | French / Caucasian | | 51 BC(25) + 45 N(13) | | 2.70 (1.10-6.61) | |  | | [19] | |  |  |
| NAT2 | rs1801280 | c.341T>C | p.Ile114Thr | NA | | MA*** | | BC() + N() | |  | |  | | [69] | |  |  |
| NAT2 | rs4646249 | g.16677T>G |  | Increased | | European | | 3532 BC() + 5120 N() | | 0.89 (0.83-0.95) | | 0.0013 | | [23] | |  |  |
| NAT2 |  | slow acetylator |  | Increased | | MA | | BC() + N() | | 1.4 (1.2–1.6) | |  | | [39] | |  |  |
| NAT2 |  | slow acetylator |  | Increased | | Spanish | | 1150 BC(728) + 1149 N(637) | | 1.4 (1.2-1.7) | | 2×10−4 | | [45] | |  |  |
| UGT1A | rs1495741 | g.18272881G>A |  | Increased | | European decent | | 11804 BC() + 52860 N() | | 0.87 (0.83-0.91) | | 4.2×10^−11^ | | [75] | |  |  |
| UGT1A10 | rs11892031 | c.855+19260A>C |  | Decreased | | MA | | BC () + N () | | 0.84 | |  | | [70] | |  |  |
| UGT1A8 | rs11892031 | c.855+19260A>C |  | Increased | | European decent | | 11808 BC() + 52835 N() | | 0.84 (0.79-0.89) | | 1×10^−7^ | | [75] | |  |  |
| UGT1A10 / UGT1A8 | rs11892031 | c.855+19260A>C |  | Decreased | | Chinese | | 367 BC(4) + 420 N(19) | | 0.27 (0.09–0.81) | | 0.019 | | [74] | |  |  |
| UGT1A10 / UGT1A8 | rs11892031 | c.855+19260A>C |  | Decreased | | European | | 3532 BC() + 5120 N() | | 0.77 (0.68-0.87) | | 3.6×10−5 | | [23] | |  |  |
| UGT1A10 / UGT1A8 | rs11892031 | c.855+19260A>C |  | Increased | | Swedish | | 11808 BC() + 52835 N() | | 0.84 (0.79–0.89) | | 1.0x10^-7^ | | [73] | |  |  |
| UGT1A10 / UGT1A8 | rs11892031 | c.855+19260A>C |  | Increased | | MA | | 1703 BC() + 1950 N() | | 1.18 (1.02–1.37) | | 0.0262 | | [72] | |  |  |
| UGT1A10 / UGT1A8 | rs11892031 | c.855+19260A>C |  | Increased | | MA | | BC() + N() | | 0.84 (0.79–0.89) | |  | | [39] | |  |  |
| UGT1A6 | rs17863783 | c.627G>T | p.Val209= | Decreased | | MA | | 4035 BC(114) + 5284 N(262) | | 0.55 (0.44–0.69) | | 3.3×10^−7^ | | [76] | |  |  |
| UGT1A6 | rs17863783 | c.627G>T | p.Val209= | Decreased | | MA | | BC () + N () | | 0.55 | |  | | [70] | |  |  |
| UGT1A8 | rs1104892 | c.856-55408G>A |  | Increased | | USA | | 718 BC(369) + 783 N(375) | | 1.3 (1.03-1.64) | | 0.03 | | [77] | |  |  |
| UGT1A8 | rs1113193 | c.855+41929G>A |  | Decreased | | USA | | 718 BC(259) + 783 N(319) | | 0.79 (0.64-0.98) | | 0.028 | | [77] | |  |  |
| UGT1A8 | rs1604144 | c.856-69845C>T |  | Decreased | | USA | | 718 BC(328) + 783 N(397) | | 0.82 (0.69-0.96) | | 0.015 | | [77] | |  |  |
| UGT1A8 | rs17854828 | c.855+18653C>A |  | Decreased | | USA | | 718 BC(157) + 783 N(205) | | 0.76 (0.6-0.96) | | 0.022 | | [77] | |  |  |
| UGT1A8 | rs17864684 | c.855+52155G>A |  | Decreased | | USA | | 718 BC(165) + 783 N (227) | | 0.72 (0.57-0.9) | | 0.005 | | [77] | |  |  |
| UGT1A8 | rs17868322 | c.855+53286G>A |  | Increased | | USA | | 718 BC(63) + 783 N(44) | | 1.63 (1.09-2.44) | | 0.019 | | [77] | |  |  |
| UGT1A8 | rs2602374 | c.855+41756C>T |  | Increased | | USA | | 718 BC(73) + 783 N(56) | | 1.57 (1.07-2.29) | | 0.02 | | [77] | |  |  |
| UGT1A8 | rs2741042 | c.855+38709C>A |  | Increased | | USA | | 718 BC(84) + 783 N(67) | | 1.49 (1.05-2.12) | | 0.026 | | [77] | |  |  |
| UGT1A8 | rs2741044 | c.855+52160G>A |  | Increased | | USA | | 718 BC(75) + 783 N(60) | | 1.49 (1.03-2.16) | | 0.034 | | [77] | |  |  |
| UGT1A8 | rs2741045 | c.855+52932C>T |  | Increased | | USA | | 718 BC(75) + 783 N(60) | | 1.49 (1.03-2.15) | | 0.036 | | [77] | |  |  |
| UGT1A8 | rs4148326 | c.856-2218T>A |  | Increased | | USA | | 718 BC(358) + 783 N(361) | | 1.33 (1.06-1.67) | | 0.015 | | [77] | |  |  |
| UGT1A8 | rs4148328 | c.1295+574C>T |  | Decreased | | USA | | 718 BC(411) + 783 N(484) | | 0.84 (0.72-0.98) | | 0.023 | | [77] | |  |  |
| UGT1A8 | rs4233633 | c.856-55743G>C |  | Decreased | | USA | | 718 BC(123) + 783 N(177) | | 0.72 (0.55-0.93) | | 0.012 | | [77] | |  |  |
| UGT1A8 | rs7571337 | c.855+39210T>C |  | Decreased | | USA | | 718 BC(336) + 783 N(391) | | 0.71 (0.56-0.9) | | 0.005 | | [77] | |  |  |
| UGT2B4 | rs2736520 | g.70336172T>C |  | Increased | | USA | | 718 BC(189) + 783 N(171) | | 1.36 (1.07-1.73) | | 0.012 | | [77] | |  |  |
| UGT2B4 | rs3822179 | c.871-907G>T |  | Decreased | | USA | | 718 BC(98) + 783 N(139) | | 0.70 (0.53-0.93) | | 0.015 | | [77] | |  |  |
| CLK3 | rs11543198 | c.597-22G>A |  | Increased | | Japanese | | 539 BC(335) + 5581 N(4894) | | 1.41 (1.26-1.59) | | 4.03×10^−9^ | | [79] | |  |  |
| Signaling | | | | | | | | | | | | | | | |  |  |
| CD274 | rs4143815 | g.5468257G>C |  | Increased | | MA / Chinese | | 293 BC() + 321 N() | | 2.015 (1.556–2.608) | | <0.001 | | [81] | |  |  |
| CTLA4 | rs231775 | c.49A>G | p.Thr17Ala | Increased | | Indian | | 200 BC(16) + 200 N(6) | | 3.74 (1.16–12.11) | | 0.028 | | [82] | |  |  |
| CTLA4 | rs3087243 | g.11411G>A |  | Increased | | Indian | | 200 BC(13) + 200 N(28) | | 1.36 (1.08–2.56) | | 0.04 | | [82] | |  |  |
| ICAM1 | rs5498 | c.1405A>G | p.Lys469Glu | Increased | | Taiwanese | | 279 BC(128) + 279 N(105) | | 1.603 (1.082–2.376) | | <0.05 | | [83] | |  |  |
| CCR2 | rs1799864 | c.190G>A | p.Val64Ile | Increased | | Turkish | | 72 BC(10) + 76 N(2) | | 5.96 (1.26-28.26) | | 0.012 | | [87] | |  |  |
| CCR2 | rs1799864 | c.190G>A | p.Val64Ile | Increased | | MA | | 387 BC(21) + 550 N(15) | | 2.06 (1.02–4.15) | | 0.11 | | [86] | |  |  |
| CCR2 | rs1799864 | c.190G>A | p.Val64Ile | Increased | | Turkish | | 142 BC(8) + 197 N(3) | | 2.39 (1.13-5.03) | | 0.022 | | [85] | |  |  |
| CCR5 | rs333 | c.554_585del or CCR5-Δ32 | p.Ser185fs | NA | | MA | | 337 BC() + 391 N() | | 1.614 (0.875–2.978) | | 0.125 | | [282] | |  |  |
| CCR5 | rs333 | c.554_585del or CCR5-Δ32 | p.Ser185fs | Increased | | Turkish | | 142 BC(11) + 197 N(4) | | 3.92 (1.23–12.46) | | 0.012 | | [85] | |  |  |
| CXCL12 | rs1801157 | g.17289G>A |  | Increased | | Indian | | 200 BC(132) + 200 N(83) | | 2.72 (1.78–4.15) | | <0.001 | | [88] | |  |  |
| CXCL12 | rs1801157 | g.17289G>A |  | Increased | | Turkish | | 142 BC(26) + 197 N(23) | | 1.5 (1.05-2.13) | | 0.023 | | [85] | |  |  |
| CXCR2 | rs1126579 | g.15722T>C |  | Decreased | | USA | | 832 BC(150) + 1191 N(84) | | 0.6 (0.5-0.9) | | <0.004 | | [89] | |  |  |
| CXCR2 | rs1126579 | c.*127T>C |  | Increased | | Indian | | 200 BC(14) + 200 N(15) | | 1.58 (0.59–4.27) | | 0.028 | | [88] | |  |  |
| CD44 | rs187115 | c.67+15242T>C |  | Increased | | Taiwanese | | 275 BC(97) + 275 N(71) | | 1.566 (1.086–2.258) | | <0.05 | | [90] | |  |  |
| IL17A | rs2275913 | g.4849G>A/C |  | Increased | | Polish | | 175 BC(97) + 207 N(89) | | 1.53 (0.99-2.36) | | 0.06 | | [92] | |  |  |
| IL18 | rs187238 | g.4853G>T/C |  | Increased | | Polish | | 175 BC(83) + 207 N(68) | | 1.84 (1.22-2.79) | | 0.008 | | [92] | |  |  |
| IL18 | NA | -137G>C |  | Increased | | North India | | 200 (112) BC+ 200 (77)N | | 1.96 (1.26–3.06) | | 0.03 | | [93] | |  |  |
| IL18 | rs1946518 | g.4383A>C |  | Increased | | North India | | 200 (89) BC+ 200 (113)N | | 1.59 (1.01–2.95) | | 0.031 | | [93] | |  |  |
| IL22 | rs2227485 | g.4672C>T |  | Increased | | Chinese | | 210 BC (58) + 210 N(33) | | 2.04 (1.19-3.49) | | 0.009 | | [94] | |  |  |
| IL10 | rs1800896 | g.3943A>G |  | Increased | | Chinese | | 400 BC() + 400 N() | | 2.058 | |  | | [97] | |  |  |
| IL10 | rs1800896 | g.3943A>G |  | Increased | | North India | | 214 BC(196) + 385 N(324) | | 1.95 (1.09-3.50) | | 0.023 | | [98] | |  |  |
| IL10 | rs1800871 | g.4206T>C |  | Increased | | Chinese | | 400 BC() + 400 N() | | 1.979 | |  | | [97] | |  |  |
| IL10 | rs1800871 | g.4206T>C |  | Increased | | North India | | 214 BC(168) + 385 N(270) | | 1.77 (1.14-2.75) | | 0.01 | | [98] | |  |  |
| IL10 | rs1800872 | g.4433A>C |  | Increased | | Chinese | | 400 BC() + 400 N() | | 1.979 | |  | | [97] | |  |  |
| IL10 | rs1800872 | g.4433A>C |  | Increased | | MA | | 4516 BC(707) + 5401 N(1031) | | 1.15 (1.02–1.31) | | 0.03 | | [96] | |  |  |
| IL23R | rs10889677 | c.309C>A |  | Increased | | Chinese | | 226 BC(321) + 270 N(441) | | 1.818 (1.349–2.449) | | <0.001 | | [99] | |  |  |
| IL23R | rs10889677 | c.309C>A |  | Increased | | Polish | | 175 BC(83) + 207 N(83) | | 1.48 (0.98-2.22) | | 0.06 | | [92] | |  |  |
| IL12 | NA | -16974A>C |  | Decreased | | North India | | 200 BC(89) + 200 N(113) | | 0.62 (0.42–0.92) | | 0.017 | | [93] | |  |  |
| IL2 | rs2069762 | g.4671T>G |  | Increased | | Chinese | | 365 BC(307) + 390 N(266) | | 1.40 (1.14–1.73) | | 0.002 | | [283] | |  |  |
| IL6 | rs1800795 | g.4880C>G/T |  | Increased | | MA | | 580 BC() + 643 N() | | 2.19 (1.32–3.64) | | 0.003 | | [284] | |  |  |
| IL6 | rs1800795 | g.4880C>G/T |  | Decreased | | India | | 232 BC(137) + 250 N(184) | | 0.52 (0.35-0.75) | | 0.00073 | | [285] | |  |  |
| IL8 and CXCL8 | rs4073 | g.4802A>C/G/T |  | Increased | | Northern India | | 205 BC(52) + 205 N(71) | | 2.12 (1.28–3.52) | | 0.003 | | [100] | |  |  |
| IL19 | rs1800890 | c.-35+4942A>T |  | Increased | | Spanish | | 1047 BC() + 988 N() | | 2.26 | | 0.019 | | [101] | |  |  |
| IL27 | rs153109 | g.28519096T>C |  | Increased | | Chinese | | 332 BC(205) + 499 N(270) | | 1.37 (1.03–1.82) | | 0.029 | | [102] | |  |  |
| IL27 | rs17855750 | c.175T>G/A | p.Ser59Ala/Thr | Increased | | Chinese | | 332 BC(4) + 499 N(0) | | 0 | | 0.006 | | [102] | |  |  |
| IL27 | rs17855750 | c.175T>G/A | p.Ser59Ala/Thr | Increased | | Chinese | | 7570 BC() + 9839 N() | | 1.156 (1.005–1.329) | | 0.41 | | [103] | |  |  |
| TNF-α | rs1799964 | g.3959T>C |  | Increased | | north India | | 220 BC(83) + 206 N(47) | | 2.23 (1.17–4.26) | | 0.014 | | [104] | |  |  |
| TGFB1 | rs1800470 | c.29C>T/G | p.Pro10Leu/Arg | Increased | | India | | 237 BC(85) + 301 N(73) | | 1.72 (1.18–2.50) | | 0.004 | | [105] | |  |  |
| TGFB1 | rs1800470 | c.29C>T/G | p.Pro10Leu/Arg | NA | | Spanish | | 1157 BC(189) + 1157 N(175) | | 1.01 (0.78–1.31) | | 0.768 | | [286] | |  |  |
| Toll-like receptors | | | | | | | | | | | | | | | |  |  |
| TLR2 | NA | -196 to -174 del | TLR2Δ22 | Increased | | North India | | 200 BC(90) + 200 N(8) | | 3.14 (2.04-4.86) | | <0.001 | | [107] | |  |  |
| TLR4 | NA | -2242T/C |  | NA | | Chinese | | 436 BC() + 522 N() | |  | |  | | [108] | |  |  |
| TLR4 | NA | 729G/C |  | NA | | Chinese | | 436 BC() + 522 N() | |  | |  | | [108] | |  |  |
| TLR4 | NA | 729G/C |  | Increased | | Chinese | | 376 BC(24) + 380 N(2) | | 3.67 (2.11-7.27) | | 0.017 | | [110] | |  |  |
| TLR4 | NA | −729G/C |  | Increased | | Chinese | | 282 BC(39) + 298 N(18) | | 2.50 (1.39–4.48) | | 0.002 | | [109] | |  |  |
| TLR4 | rs11536889 | g.16672G>C |  | Increased | | Chinese | | 436 BC() + 522 N() | | 2.33 (1.52–3.58) | | < 0.0001 | | [108] | |  |  |
| TLR7 | rs72552316 | g.27509T>C |  | Increased | | Chinese | | 317 BC() + 268 N() | |  | | ≤0.0001 | | [111] | |  |  |
| Transcription factors | | | | | | | | | | | | | | | |  |  |
| VDR | rs2228570 | c.2T>G/C/A | p.Met1Arg/Thr/Lys | Increased | | North India | | 130 BC(74) + 346 N(151) | | 2.042 (0.803-5.193) | | 0.033 | | [113] | |  |  |
| VDR | rs2228570 | c.2T>G/C/A | p.Met1Arg/Thr/Lys | Increased | | Tunisian | | 200 BC(24) + 200 N(11) | | 2.66 (1.24–5.73) | | 0.012 | | [114] | |  |  |
| TP63 | rs4687100 | c.1747-969A>G |  | Increased | | MA | | 1601 BC() + 1819 N() | |  | |  | | [116] | |  |  |
| TP63 | rs710521 | g.189645933T>C/G |  | Increased | | MA | | BC() + N() | | 1.19 (1.12–1.27) | |  | | [39] | |  |  |
| TP63 | rs710521 | g.189645933T>C/G |  | Increased | | MA / Chinese | | 1050 BC() + 1404 N() | | 1.14 | | 0.061 | | [70] | |  |  |
| TP63 | rs710521 | g.189645933T>C/G |  | Increased | | MA | | 1601 BC() + 1819 N() | | 0.78 (0.68–0.90) | | 0.00034741 | | [116] | |  |  |
| TP63 | rs710521 | g.189645933T>C/G |  | Decreased | | India | | 225 BC(17) + 240 N(19) | | 0.53 (0.18–1.16) | | 0.045 | | [117] | |  |  |
| TP63 | rs710521 | g.189645933T>C/G |  | NA | | Chinese | | 415 BC() + 465 N() | | 1.48 (0.80–2.76) | | 0.216 | | [118] | |  |  |
| TP63 | rs35592567 | g.270421C>G/T |  | Increased | | MA | |  | |  | |  | | [11] | |  |  |
| TP63 | rs35592567 | g.270421C>G/T |  | Decreased | | MA / European | | 3094 BC() + 3738 N() | | 0.82 (0.75-0.90) | | 9.797×10^-6^ | | [119] | |  |  |
| Vascular endothelial growth factor | | | | | | | | | | | | | | | |  |  |
| VEGF | rs699947 | g.3437A>C/T |  | Increased | | North India | | 200 BC(116 ) + 250 N(112) | | 1.69 (1.02–2.80) | | 0.044 | | [121] | |  |  |
| VEGF | NA | 1001G/C |  | Increased | | North India | | 200 BC(13) + 250 N(8) | | 3.29 (1.10–9.72) | | 0.032 | | [121] | |  |  |
| VEGF | rs35569394 | -2549I/D | 18-bp insertion/deletion | Decreased | | North India | | 200 BC(41) + 250 N(68) | | 0.53 (0.29–0.96) | | 0.037 | | [121] | |  |  |
| VEGFA | rs833052 | g.43723335A>C/G/T |  | Increased | | MA | | 1745 BC() + 2032 N() | | 2.33 (1.67–3.26) | | 0 | | [122] | |  |  |
| VEGFA | rs25648 | c.534C>G/T | p.Ser178= | Increased | | MA | | 1645 BC() + 2009 N() | | 2.57 (1.12–5.90) | | 0.026 | | [122] | |  |  |
| VEGFA | rs3025039 | g.19584C>T |  | NA | | Tunisian | | 218 BC(88) + 204 N(75) | | 1.16 (0.78‐1.72) | | 0.448 | | [125] | |  |  |
| VEGFA | rs3025039 | g.19584C>T |  | Increased | | MA | | 2614 BC() + 3199 N() | | 1.48 (1.10–1.98) | | 0.009 | | [122] | |  |  |
| VEGFA | rs3025039 | g.19584C>T |  | Increased | | MA | | 4359 BC(2614) + 5417 N(3385) | | 1.51 (1.13‐2.02) | | 0.005 | | [123] | |  |  |
| VEGFA | rs699947 | g.3437A>C/T |  | Decreased | | Tunisian | | 218 BC(100) + 204 N(106) | | 0.62 (0.41-0.94) | | 0.026 | | [125] | |  |  |
| VEGFA | rs699947 | g.3437A>C/T |  | Increased | | MA | | 1031 BC() + 1845 N() | | 1.48 (1.17-1.89) | | <0.05 | | [124] | |  |  |
| VEGFA | rs699947 | g.3437A>C/T |  | NA | | MA | | 1942 BC() + 2307 N() | | 0.99 (0.74,1.33) | | 0.959 | | [122] | |  |  |
| VEGFC | rs3775194 | c.704+8776G>C |  | Increased | | USA / Caucasian | | 801 BC(545) + 801 N(486) | | 1.27 (1.08–1.49) | | 3.28×10^−3^ | | [126] | |  |  |
| VEGFC | rs1485762 | c.1145+610A>T |  | Increased | | USA / Caucasian | | 801 BC(438) + 801 N(383) | | 1.42 (1.19–1.69) | | 8.12x10^-5^ | | [126] | |  |  |
| VEGFC | rs6828869 | c.705-1052G>T |  | Increased | | USA / Caucasian | | 801 BC(573) + 801 N(529) | | 1.30 (1.10–1.54) | | 1.91x10^-3^ | | [126] | |  |  |
| VEGFC | rs17697515 | c.705-1343G>A |  | Increased | | USA / Caucasian | | 801 BC(123) + 801 N(93) | | 1.57 (1.14–2.17) | | 5.82x10^-3^ | | [126] | |  |  |
| VEGFC | rs1485766 | c.705-1803A>T |  | Increased | | Taiwanese | | 233 BC(170) + 520 N(330) | | 1.554 (1.106-2.182) | | <0.05 | | [127] | |  |  |
| VEGFR | rs4557213 | c.147+24186C>T |  | Increased | | USA / Caucasian | | 801 BC(163) + 801 N(124) | | 1.50 (1.13–1.99) | | 4.69×10^−3^ | | [126] | |  |  |
| HLA-G | rs1063320 | g.8994C>G/T |  | Increased | | Brazilian | | 160 BC() + 214 N() | | 6.965 (0.8659–56.029) | | 0.0474 | | [129] | |  |  |
| HLA-G | rs1610696 | g.9048C>G |  | Increased | | Brazilian | | 160 BC() + 214 N() | | 6.965 (0.8659–56.029) | | 0.0474 | | [129] | |  |  |
| HLA-G | rs1704 | 14-bp insertion/deletion |  | Increased | | Brazilian | | 160 BC() + 214 N() | | 6.965 (0.8659–56.029) | | 0.0474 | | [129] | |  |  |
| HLA-G | rs1707 | g.8855C>A/T |  | Increased | | Brazilian | | 160 BC() + 214 N() | | 6.965 (0.8659–56.029) | | 0.0474 | | [129] | |  |  |
| HLA-G | rs1710 | g.8862G>C |  | Increased | | Brazilian | | 160 BC() + 214 N() | | 6.965 (0.8659–56.029) | | 0.0474 | | [129] | |  |  |
| HLA-G | rs17179101 | g.8879C>A |  | Increased | | Brazilian | | 160 BC() + 214 N() | | 6.965 (0.8659–56.029) | | 0.0474 | | [129] | |  |  |
| HLA-G | rs17179108 | g.8887C>T |  | Increased | | Brazilian | | 160 BC() + 214 N() | | 6.965 (0.8659–56.029) | | 0.0474 | | [129] | |  |  |
| Others | | | | | | | | | | | | | | | |  |  |
| EGFR | rs6593205 | c.89-41287A>G |  | Decreased | | USA / Caucasian | | 801 BC(502) + 801 N(544) | | 0.76 (0.65–0.90) | | 1.17x10^-3^ | | [126] | |  |  |
| EGFR | rs7799627 | c.89-48823G>A |  | Decreased | | USA / Caucasian | | 801 BC(65) + 801 N(93) | | 0.59 (0.41–0.86) | | 5.08x10^-3^ | | [126] | |  |  |
| EGFR | rs11238349 | c.89-53908G>A |  | Increased | | USA / Caucasian | | 801 BC(405) + 801 N(359) | | 1.28 (1.08–1.51) | | 4.28x10^-3^ | | [126] | |  |  |
| EGFR | rs1050171 | c.2361G>A/C | p.Gln787=/His | Increased | | Chinese | | 908 BC(227) + 1239 N(256) | | 1.29 (1.05–1.58) | | 0.017 | | [130] | |  |  |
| NFKBIA | rs696 | g.7852G>C |  | Increased | | Spanish | | 1047 BC() + 988 N() | | 2.47 | | 0.004 | | [101] | |  |  |
| BLNK | rs11188660 | c.1096-505C>T |  | Increased | | Spanish | | 1047 BC() + 988 N() | | 2.56 | | 0.004 | | [101] | |  |  |
| NFKB1 | rs28362491 | g.4670_4673ATTG |  | Increased | | Chinese | | 609 BC(151) + 640 N(93) | | 1.92 (1.40–2.59) | | <0.001 | | [132] | |  |  |
| NFKB1 | rs28362491 | g.4670_4673ATTG |  | Increased | | Chinese | | 730 BC(187) + 780 N(124) | | 1.82 (1.41-2.35) | | <0.001 | | [131] | |  |  |
| NFKB1 | rs28362491 | g.4670_4673ATTG |  | Increased | | Chinese | | 207 BC(270) + 228 N(256) | | 1.465 (1.114–1.927) | | 0.006 | | [133] | |  |  |
| NFKB1 | rs28362491 | g.4670_4673ATTG |  | NA | | MA | | 1058 BC() + 1175 N() | | 1.03 (0.70-1.51) | | 0.89 | | [287] | |  |  |
| MAML2 | rs7944701 | c.514-4342G>T |  | Increased | | Chinese | | 580 BC()+ 1101 N() | | 1.329 (1.115-1.583) | | 0.001 | | [134] | |  |  |
| MCP1 and CCL2 | rs1024611 | g.2493A>G |  | Decreased | | Mexican | | 47 BC(3) + 126 N(37) | | 6.097 (1.885-19.570) | | 0.001 | | [288] | |  |  |
| MCP1 and CCL2 | rs1024611 | g.2493A>G |  | Increased | | Turkish | | 72 BC(8) + 76 N(3) | | 3.04 (0.77-11.9) | | 0.08 | | [87] | |  |  |
| ING2 | rs6854224 | g.184420872C>T |  | Decreased | | Caucasian | | 803 BC() + 803 N() | | 0.70 (0.53-0.93) | | 0.013 | | [173] | |  |  |
| ING2 | rs11132186 | g.184438723G>C/T |  | Decreased | | Caucasian | | 803 BC() + 803 N() | | 0.52 (0.32-0.83) | | 0.005 | | [173] | |  |  |
| ING2 | rs11735038 | g.184440980A>C/T |  | Decreased | | Caucasian | | 803 BC() + 803 N() | | 0.66 (0.49-0.90) | | 0.008 | | [173] | |  |  |
| CCN4 | rs2977530 | c.70-9995G>A |  | Increased | | Taiwan | | 369 BC(265) + 738 N(527) | | 1.021 (0.698–1.494) | |  | | [289] | |  |  |
| Cell death | | | | | | | | | | | | | | | |  |  |
| FAS | rs2234767 | c.112G>A/T | p.Ala38Thr/Ser | Increased | | Turkish | | 91 BC(67) + 101 N(52) | | 1.403 (1.123-1.753) | | 0.003 | | [136] | |  |  |
| FASLG | rs763110 | g.4314C>T |  | Increased | | Turkish | | 91 BC(91) + 101 N(86) | | 1.208 (1.021-1.428) | | 0.027 | | [136] | |  |  |
| TNFSF10 | rs1131580 | g.172223620A>C/G/T |  | Increased | | Turkish | | 91 BC(138) + 139 N(184) | | 1.256 (1.138–1.386) | | < 0.001 | | [137] | |  |  |
| DR4 | rs6557634 | c.422A>G | p.His141Arg | Increased | | North India | | 200 BC(22) + 225 N(13) | | 2.51 (1.06-5.94) | | 0.036 | | [138] | |  |  |
| TNFRSF10A | rs13278062 | g.4710C>T/G/A |  | Increased | | Chinese | | 368 BC(216) + 368 N(172) | | 1.55 (1.15-2.09) | | 0.005 | | [139] | |  |  |
| TNFRSF10A | rs20575 | c.626G>T/C | p.Arg209Ile/Thr | Decreased | | Caucasian | | 253 BC(62) + 215 N(76) | | 0.59 (0.40–0.89) | |  | | [140] | |  |  |
| BCL10 | rs2647396 | c.58-1841G>C |  | Increased | | Spanish | | 1047 BC() + 988 N() | | 0.4 | | 0.011 | | [101] | |  |  |
| PRF1 | rs10999426 | c.539+82C>T |  | Increased | | Spanish | | 1047 BC() + 988 N() | | 2.11 | | 0.019 | | [101] | |  |  |
| RIPK2 | rs42490 | c.483+789G>A |  | Increased | | Spanish | | 275 BC(90) +349 N(75) | | 1.46 (1.01–2.10) | | 0.042 | | [141] | |  |  |
| CLPTM1L | rs401681 | c.1316-153G>A |  | Increased | | Swedish | | 7673 BC() + 40105 N() | | 1.11 (1.07-1.16) | | 5.0x10^-7^ | | [73] | |  |  |
| CLPTM1L | rs401681 | c.1316-153G>A |  | Increased | | MA | | BC() + N() | | 1.12 (1.06-1.18) | |  | | [39] | |  |  |
| CLPTM1L | rs401681 | c.1316-153G>A |  | Increased | | Chinese | | 367 BC(339) + 420 N(367) | | 1.79 (1.10–2.91) | | 0.02 | | [74] | |  |  |
| Casp3 | rs4647603 | c.-16+819G>A |  | Increased | | North India | | 200 BC(128) + 225 N(96) | | 2.12 (1.34-3.36) | | 0.001 | | [138] | |  |  |
| Casp5 | rs3181320 | c.37T>C | p.Phe13Leu | Increased | | North India | | 200 BC(125) + 225 N(129) | | 1.68 (1.03-2.66) | | 0.026 | | [138] | |  |  |
| Casp5 | rs507879 | c.268A>G | p.Ala90Thr | Increased | | North India | | 200 BC(183) + 225 N(187) | | 0.83 (0.90-1.56) | | 0.036 | | [138] | |  |  |
| CASP8 | rs3834129 | g.4352_4357del | 652 6N ins/del | Decreased | | Chinese | | 365 BC(115) + 368 N(138) | | 0.72 (0.53-0.99) | | 0.041 | | [142] | |  |  |
| CASP9 | rs4645978 | g.4252G>T |  | Decreased | | North India | | 212 BC(28) + 250 N(62) | | 0.487 (0.28–0.84) | | 0.01 | | [143] | |  |  |
| HMGB1 | rs1045411 | g.31033232C>T |  | Decreased | | Chinese | | 431 BC(148) + 862 N(359) | | 0.722 (0.565-0.924) | | <0.05 | | [144] | |  |  |
| DNA Repair | | | | | | | | | | | | | | | |  |  |
| Base excision repair | | | | | | | | | | | | | | | |  |  |
| XRCC1 | rs1799782 | c.580C>T | p.Arg194Trp | Increased | | Chinese | | 234 BC(23) + 253 N(9) | | 3.90 (1.69-8.98) | | 0.008 | | [146] | |  |  |
| XRCC1 | rs1799782 | c.580C>T | p.Arg194Trp | Increased | | MA | | 4751 BC() + 6102 N() | | 1.69 (1.25-2.28) | | 0.001 | | [148] | |  |  |
| XRCC1 | rs1799782 | c.580C>T | p.Arg194Trp | Increased | | MA / Asian | | 1931 BC() + 2192 N() | | 1.12 (1.02–1.41) | | <0.05 | | [149] | |  |  |
| XRCC1 | rs1799782 | c.580C>T | p.Arg194Trp | Increased | | MA / asian | | 1284 BC() + 1326 N() | | 1.199 (1.021-1.408) | | <0.05 | | [150] | |  |  |
| XRCC1 | rs1799782 | c.580C>T | p.Arg194Trp | Increased | | MA / asian | | 4977 BC() + 6186 N() | | 1.327 (1.086-1.622) | | 0.006 | | [151] | |  |  |
| XRCC1 | rs1799782 | c.580C>T | p.Arg194Trp | NA | | MA | | 5228 BC() + 6633 N() | | 1.78 (1.12–2.82) | | 0.49 | | [152] | |  |  |
| XRCC1 | rs1799782 | c.580C>T | p.Arg194Trp | Increased | | North India | | 195 BC(40) + 250 N(43) | | 1.10 (0.68–1.78) | | 0.674 | | [147] | |  |  |
| XRCC1 | rs1799782 | c.580C>T | p.Arg194Trp | NA | | French | | 51 BC(4) + 45 N(5) | | 1.50 (0.37-6.08) | |  | | [155] | |  |  |
| XRCC1 | rs1799782 | c.580C>T | p.Arg194Trp | NA | | Caucasian | | 547 BC(59) + 579 N(64) | | 0.95 (0.65–1.40) | | 0.81 | | [290] | |  |  |
| XRCC1 | rs1799782 | c.580C>T | p.Arg194Trp | NA | | MA | | 3091 BC() + 3219 N() | | 0.90 (0.77-1.05) | | 0.17 | | [156] | |  |  |
| XRCC1 | rs25489 | c.839G>C/A | p.Arg280Pro/His | Increased | | North India | | 195 BC(58) + 250 N(41) | | 1.94 (1.20–3.14) | | 0.006 | | [147] | |  |  |
| XRCC1 | rs25489 | c.839G>C/A | p.Arg280Pro/His | Increased | | MA / Asian | | 1931 BC() + 2192 N() | | 1.75 (1.05–2.90) | | 0.01 | | [149] | |  |  |
| XRCC1 | rs25489 | c.839G>C/A | p.Arg280Pro/His | Increased | | MA / asian | | 822 BC() + 881 N() | | 1.869 (1.205-2.898) | | 0.011 | | [150] | |  |  |
| XRCC1 | rs25489 | c.839G>C/A | p.Arg280Pro/His | Increased | | MA / asian | | 2516 BC() + 2579 N() | | 2.094 (1.211–3.621) | | 0.008 | | [151] | |  |  |
| XRCC1 | rs25489 | c.839G>C/A | p.Arg280Pro/His | Increased | | MA* | | 2413 BC() + 2477 N() | | 1.63 (1.24–2.13) | | <0.05 | | [152] | |  |  |
| XRCC1 | rs25489 | c.839G>C/A | p.Arg280Pro/His | Increased | | Chinese | | 234 BC(88) + 253 N(52) | | 2.53 (1.67-3.83) | | <0.001 | | [146] | |  |  |
| XRCC1 | rs25489 | c.839G>C/A | p.Arg280Pro/His | Increased^1^ | | Caucasian | | 547 BC(57) + 579 N(44) | | 1.50 (0.98-2.28) | | 0.06 | | [290] | |  |  |
| XRCC1 | rs25489 | c.839G>C/A | p.Arg280Pro/His | NA | | MA | | 2547 BC() + 1784 N() | | 0.99 (0.55-1.77) | | 0.97 | | [156] | |  |  |
| XRCC1 | rs915927 | c.618A>T/G/C | p.Pro206= | Increased | | Italy / Turin | | 456 BC(234) + 376 N(222) | | 1.55 (1.02-2.37) | |  | | [153] | |  |  |
| XRCC1 | rs915927 | c.618A>T/G/C | p.Pro206= | NA | | Caucasian | | 547 BC(260) + 579 N(270) | | 0.96 (0.73–1.28) | |  | | [290] | |  |  |
| XRCC1 | rs25487 | c.1196A>G/C | p.Gln399Arg/Pro | Increased^1^ | | North India | | 195 BC(106) + 250 N(109) | | 1.51 (0.99–2.31) | | 0.051 | | [147] | |  |  |
| XRCC1 | rs25487 | c.1196A>G/C | p.Gln399Arg/Pro | Increased | | MA | | 5654 BC() +7136 N() | | 1.10 (1.03-1.19 | | 0.008 | | [148] | |  |  |
| XRCC1 | rs25487 | c.1196A>G/C | p.Gln399Arg/Pro | Increased | | MA / Non-Asians | | 4747 BC(2515) + 5993 N(3033) | | 1.091 (1.008-1.180) | | 0.032 | | [154] | |  |  |
| XRCC1 | rs25487 | c.1196A>G/C | p.Gln399Arg/Pro | Decreased | | USA | | 355 BC(86) + 544 N(36) | | 0.6 (0.4-1.0) | |  | | [291] | |  |  |
| XRCC1 | rs25487 | c.1196A>G/C | p.Gln399Arg/Pro | NA | | French | | 51 BC(25) + 45 N(18) | | 0.75 (0.42-1.35) | |  | | [155] | |  |  |
| XRCC1 | rs25487 | c.1196A>G/C | p.Gln399Arg/Pro | NA | | MA / Asian | | 1931 BC() + 2192 N() | | 0.93 (0.70–1.24) | | 0.01 | | [149] | |  |  |
| XRCC1 | rs25487 | c.1196A>G/C | p.Gln399Arg/Pro | NA | | Turkish | | 75 BC() + 100 N() | | 0.72 (0.41-1.26) | | 0.16 | | [171] | |  |  |
| XRCC1 | rs25487 | c.1196A>G/C | p.Gln399Arg/Pro | NA | | Caucasian | | 547 BC(248) + 579 N(259) | | 0.97 (0.75–1.26) | | 0.83 | | [290] | |  |  |
| XRCC1 | rs25487 | c.1196A>G/C | p.Gln399Arg/Pro | NA | | MA | | 3729 BC() + 3927 N() | | 0.95 (0.82-1.10) | | 0.48 | | [156] | |  |  |
| XRCC1 | rs25487 | c.1196A>G/C | p.Gln399Arg/Pro | NA | | UK | | 194 BC(107) + 313 N(156) | | 0.9 (0.6–1.4) | | >0.05 | | [157] | |  |  |
| XRCC1 | rs25487 | c.1196A>G/C | p.Gln399Arg/Pro | NA | | MA | | 5767 BC() + 6919 N() | | 0.97 (0.85–1.10) | | 0.596 | | [158] | |  |  |
| XRCC1 | rs25487 | c.1196A>G/C | p.Gln399Arg/Pro | NA | | MA | | 5759 BC() + 6521 N() | | 0.96 ( 0.80-1.16) | | <0.00001 | | [159] | |  |  |
| XRCC2 | rs3218373 | g.4088G>T/A |  | Decreased | | Spanish | | 1150 BC(6) + 1149 N(16) | | 0.36 (0.13–0.96) | | 0.04 | | [160] | |  |  |
| XRCC2 | rs3218536 | c.563G>C/A | p.Arg188His/Pro | Decreased | | Spanish | | 1150 BC(6) + 1149 N(13) | | 0.36 (0.13–1.00) | | 0.05 | | [160] | |  |  |
| XRCC2 | rs6464268 | g.152381150A>G |  | Decreased | | Spanish | | 1150 BC(12) + 1149 N(25) | | 0.40 (0.19–0.84) | | 0.01 | | [160] | |  |  |
| XRCC4 | rs1805377 | c.894-7G>A |  | Increased | | Spanish | | 1150 BC() + 1149 N() | | 1.33 (1.08–1.64) | | 0.01 | | [160] | |  |  |
| XRCC4 | rs1805377 | c.894-29390_894-29389insCCT |  | Increased | | India | | 211 BC(82) + 244 N(66) | | 1.94 (1.12–3.34) | | 0.017 | | [161] | |  |  |
| XRCC4 | rs1805377 | c.894-7G>A |  | NA | | India | | 211 BC(70) + 244 N(79) | | 1.13 (0.72–1.78) | | 0.574 | | [161] | |  |  |
| XRCC4 | rs6869366 | g.3519T>G |  | Increased | | Taiwanese | | 158 BC() + 158 N() | | 1.85 (1.15-2.98) | | 0.00994 | | [162] | |  |  |
| XRCC4 | rs6869366 | g.3519T>G |  | Decreased | | India | | 211 BC(8) + 244 N(17) | | 0.27 (0.09–0.81) | | 0.02 | | [161] | |  |  |
| XRCC5 | rs828907 | g.3713G>T |  | Increased | | Taiwanese | | 288 BC(128) + 288 N(91) | |  | | 0.0055 | | [163] | |  |  |
| XRCC7 | rs3213245 | g.5044C>T/A |  | Increased | | southwestern Han Chinese | | 302 BC(185) + 311 N(152) | | 2.36 (1.13−4.92) | | 0.022 | | [164] | |  |  |
| PRKDC | rs7003908 | c.6465+375G>T |  | Increased | | North India | | 212 BC(99) + 250 N(54) | | 4.45 (2.56-7.74) | | 0.001 | | [165] | |  |  |
| PRKDC | rs7003908 | c.6465+375G>T |  | NA | | MA / Asian | | 727 BC(381) + 796 N(446) | | 1 (0.43-2.34) | | <0.000001 | | [292] | |  |  |
| APEX1 | rs1760944 | g.4860T>C/G |  | Decreased | | Chinese | | 234 BC(34) + 253 N(52) | | 0.57 (0.33-0.98) | | <0.05 | | [146] | |  |  |
| APE1 | rs1130409 | c.444T>A | p.Asp148Glu | Increased | | North India | | 195 BC(4) + 250 N(17) | | 0.25 (0.08–0.78) | | 0.017 | | [147] | |  |  |
| APE1 | rs1130409 | c.444T>A | p.Asp148Glu | Decreased | | North india | | 206 BC(4) + 250 N(17) | | 0.27 (0.08-0.86) | | 0.027 | | [166] | |  |  |
| APE1 | rs1130409 | c.444T>A | p.Asp148Glu | NA | | MA | | 3968 BC() + 4475 N() | | 0.984 (0.897–1.078) | | 0.528 | | [167] | |  |  |
| POLB | rs3136717 | c.62-89C>G |  | Increased | | Spanish | | 1150 BC(257) + 1149 N(202) | | 1.30 (1.04-1.62) | | 0.05 | | [168] | |  |  |
| hOGG1 | rs1052133 | c.977C>G | p.Ser326Cys | Increased | | Chinese | | 1050 BC(706) +1402 N(888) | | 1.19 (1.00–1.41) | | 0.042 | | [169] | |  |  |
| OGG1 | rs1052133 | c.977C>G | p.Ser326Cys | Increased | | Turkish | | 75 BC() + 100 N() | | 2.41 (1.36-4.25) | | 0.002 | | [171] | |  |  |
| OGG1 | rs1052133 | c.977C>G | p.Ser326Cys | Increased | | North India | | 212 BC(27) + 250 N(17) | | 2.10 (1.08-4.09) | | 0.028 | | [165] | |  |  |
| OGG1 | rs1052133 | c.977C>G | p.Ser326Cys | Increased | | North India | | 195 BC(27) + 250 N(17) | | 2.10 (1.08–4.09) | | 0.027 | | [147] | |  |  |
| OGG1 | rs1052133 | c.977C>G | p.Ser326Cys | Increased | | Belarusian | | 335 BC(94) + 366 N(132) | | 0.69 (0.50–0.95) | | 0.024 | | [170] | |  |  |
| hogg1 | rs1052133 | c.977C>G | p.Ser326Cys | NA | | MA | | 2474 BC (1183) + 2408 N(1157) | | 1.07 (0.87-1.32) | | 0.53 | | [293] | |  |  |
| hOGG1 | rs1052133 | c.977C>G | p.Ser326Cys | NA | | MA | | 2474 BC(241) + 2498 N(226) | | 1.05 (0.65-1.70) | | 0.85 | | [294] | |  |  |
| hOGG1 | rs1052133 | c.977C>G | p.Ser326Cys | NA | | MA | | 2521 BC() +2408 N() | | 1.07 (0.81-1.42) | | 0.001 | | [295] | |  |  |
| OGG1 | rs125701 | g.3851G>A |  | Decreased | | Spanish | | 1150 BC(248) + 1149 N(280) | | 0.78 (0.63-0.96) | | 0.02 | | [168] | |  |  |
| SMUG1 | rs2029167 | g.54590133G>A |  | Increased | | USA | | 801 BC(196) + 801 N(148) | | 1.42 (1.11-1.82) | | 0.005 | | [172] | |  |  |
| MUTYH | rs3219487 | g.12588A>T/G |  | Increased | | USA | | 801 BC(149) + 801 N(117) | | 1.37 (1.04-1.80) | | 0.024 | | [172] | |  |  |
| DDB2 | rs11039130 | g.47229316C>T |  | Increased | | Caucasian | | 803 BC() + 803 N() | | 1.64 (1.14-2.35) | | 0.007 | | [173] | |  |  |
| Nucleotide excision repair | | | | | | | | | | | | | | | |  |  |
| ERCC1 | rs3212961 | c.525+33C>T |  | Increased | | Spanish | | 1150 BC(279) + 1149 N(243) | | 1.2 (1.0-1.5) | | 0.06 | | [176] | |  |  |
| ERCC1 | rs967591 | g.77153C>T/G/A |  | Decreased | | Italy / Turin | | 456 BC(325) + 376 N(288) | | 0.66 (0.46-0.95) | |  | | [153] | |  |  |
| ERCC1 | rs735482 | g.75085T>G |  | Decreased | | Italy / Turin | | 456 BC(324) + 376 N(283) | | 0.62 (0.42-0.90) | |  | | [153] | |  |  |
| ERCC1 | rs2336219 | g.74681C>T |  | Decreased | | Italy / Turin | | 456 BC(323) + 376 N(275) | | 0.63 (0.43-0.93) | |  | | [153] | |  |  |
| ERCC2 | rs13181 | c.2251A>C | p.Lys751Gln | Increased | | MA / caucasian | | 6360 BC() + 7894 N() | | 1.14 (1.01–1.29) | | 0.043 | | [177] | |  |  |
| ERCC2 | rs13181 | c.2251A>C | p.Lys751Gln | Increased | | MA | | 7062 BC() + 8832 N() | | 1.10 (1.03–1.18) | | 0.004 | | [178] | |  |  |
| ERCC2 | rs13181 | c.2251A>C | p.Lys751Gln | Increased | | Chinese | | 215 BC(48) + 245 N(34) | | 1.65 (1.12-2.73) | | 0.033 | | [179] | |  |  |
| ERCC2 | rs13181 | c.2251A>C | p.Lys751Gln | Increased | | India | | 270 BC(196) + 252 N(148) | | 1.86 (1.23-2.73) | | 0.0008 | | [180] | |  |  |
| ERCC2 | rs13181 | c.2251A>C | p.Lys751Gln | Increased | | Belarusian | | 354 BC() + 418 N() | | 0.67 | | 0.012 | | [181] | |  |  |
| ERCC2 | rs13181 | c.2251A>C | p.Lys751Gln | Increased | | MA / Asian | | 6836 BC() + 8251 N() | | 1.13 (1.02–1.26) | | 0.02 | | [296] | |  |  |
| ERCC2 | rs13181 | c.2251A>C | p.Lys751Gln | Increased | | MA | | 5368 BC(764) + 6683 N(876) | | 1.12 (1.01-1.25) | | 0.04 | | [185] | |  |  |
| ERCC2 | rs13181 | c.2251A>C | p.Lys751Gln | Increased | | Tunisian | | 140 BC() + 200 N() | | 0.94 (0.88-1.0) | | 0.0535 | | [15] | |  |  |
| XPD | rs13181 | c.2251A>C | p.Lys751Gln | Increased | | UK | | 194 BC(130) + 313 N(116) | | 2.7 (1.8–3.9) | | 0.023 | | [157] | |  |  |
| ERCC2 | rs13181 | c.2251A>C | p.Lys751Gln | NA | | MA | | 5425 BC(2906) + 5669 N(3007) | | 1.03 (0.95–1.11) | | 0.53 | | [297] | |  |  |
| ERCC2 | rs13181 | c.2251A>C | p.Lys751Gln | NA | | MA | | 3258 BC(48) + 3399 N(33) | | 1.07 (0.97–1.18) | | <0.05 | | [184] | |  |  |
| ERCC2 | rs13181 | c.2251A>C | p.Lys751Gln | NA | | USA | | 210 BC(29) + 229 N(31) | | 0.8 (0.4-1.4) | |  | | [298] | |  |  |
| XPD | rs13181 | c.2251A>C | p.Lys751Gln | NA | | French | | 51 BC(18) + 45 N(17) | | 0.80 (0.46-1.39) | |  | | [155] | |  |  |
| ERCC2 | rs1799793 | c.934G>A | p.Asp312Asn | Increased | | Belarusian | | 418 BC() + 354 N() | | 0.67 | | 0.012 | | [181] | |  |  |
| ERCC2 | rs1799793 | c.934G>A | p.Asp312Asn | Increased | | USA | | 702 BC(625) + 632(591) | | 1.28 (1.01–1.62) | |  | | [183] | |  |  |
| ERCC2 | rs1799793 | c.934G>A | p.Asp312Asn | Increased | | North India | | 206 BC(34) + 250 N(18) | | 3.30 (1.72-6.35) | | 0.001 | | [166] | |  |  |
| ERCC2 | rs1799793 | c.934G>A | p.Asp312Asn | Increased | | MA | | 2302 BC(612) + 2486 N(584) | | 1.14 (1.01–1.28) | | <0.05 | | [184] | |  |  |
| ERCC2 | rs1799793 | c.934G>A | p.Asp312Asn | Increased | | MA | | 3797 BC() + 5094 N() | | 1.26 (1.11–1.42) | | 0.001 | | [177] | |  |  |
| ERCC2 | rs1799793 | c.934G>A | p.Asp312Asn | Increased | | MA | | 4139 BC() + 5643 N() | | 1.29 (1.13–1.48) | | <0.001 | | [178] | |  |  |
| ERCC2 | rs1799793 | c.934G>A | p.Asp312Asn | Increased | | Belarusian | | 333 BC(178) + 364 N(169) | | 1.35 (1.0–1.82) | | 0.047 | | [170] | |  |  |
| ERCC2 | rs1799793 | c.934G>A | p.Asp312Asn | Increased | | MA | | 6440 BC(2279) + 8782 N(2971) | | 1.15 (1.04-1.28) | | 0.009 | | [185] | |  |  |
| ERCC2 | rs1799793 | c.934G>A | p.Asp312Asn | Increased | | Taiwanese | | 308 BC(155) + 308 N(109) | | 1.85 (1.34-2.56) | | 0.0086 | | [186] | |  |  |
| ERCC2 | rs1799793 | c.934G>A | p.Asp312Asn | NA | | French | | 51 BC(19) + 45 N(18) | | 0.91 (0.51-1.64) | |  | | [155] | |  |  |
| ERCC2 | rs238406 | c.468A>C | p.Arg156= | Increased | | Spanish | | 1150 BC(865) + 1149 N(798) | | 1.3 (1.1-1.6) | | 0.006 | | [176] | |  |  |
| ERCC2 | rs238406 | c.468A>C | p.Arg156Arg | Increased | | MA | | 1348 BC() + 1370 N() | | 1.36 (1.15–1.61) | | <0.001 | | [178] | |  |  |
| ERCC2 | rs238406 | c.468A>C | p.Arg156Arg | Increased | | Chinese | | 215 BC(154) + 245 N(153) | | 1.54 (1.19-2.01) | | 0.002 | | [179] | |  |  |
| ERCC5 | rs1047769 | c.760A>G | p.Met254Val | Increased | | Spanish | | 1150 BC(98) + 1149 N(73) | | 1.4 (1.0-2.0) | | 0.04 | | [176] | |  |  |
| ERCC5 | rs17655 | c.3310G>C | p.Asp1104His | Increased | | Han-Chinese | | 72 BC(57) + 278 N(233) | | 1.07 (0.86–1.87) | | 0.048 | | [187] | |  |  |
| ERCC5 | rs17655 | c.3310G>C | p.Asp1104His | NA | | MA | | 2613 BC(1215) + 2934 N(1415) | | 1.08 (0.85–1.38) | | 0.52 | | [299] | |  |  |
| ERCC6 | rs2228526 | c.3289A>G | p.Met1097Val | Increased | | Belarusian | | 418 BC() + 354 N() | |  | |  | | [181] | |  |  |
| ERCC6 | rs2228526 | c.3289A>G | p.Met1097Val | NA | | MA | | 1419 BC() + 1568 N() | | 1.10 (0.97-1.25) | | 0.449 | | [272] | |  |  |
| ERCC6 | rs2228526 | c.3289A>G | p.Met1097Val | NA | | Taiwanese | | 288 BC(40) + 288 N(46) | | 0.85 (0.54-1.34) | | <0.05 | | [188] | |  |  |
| ERCC6 | rs2228528 | c.1196G>A | p.Gly399Asp | Increased | | Belarusian | | 418 BC() + 354 N() | |  | |  | | [181] | |  |  |
| ERCC6 | rs2228528 | c.1196G>A | p.Gly399Asp | Increased | | Taiwanese | | 288 BC(196) + 288 N(176) | | 1.36 (0.96-1.91) | | <0.05 | | [188] | |  |  |
| GTF2H1 | rs4150667 | c.1560+657C>A |  | Increased | | Caucasian | | 803 BC() + 803 N() | | 1.55 (1.12-2.15) | | 0.008 | | [173] | |  |  |
| RAD23B | rs1805335 | c.554-15A>G |  | Increased | | Spanish | | 1150 BC(787) + 1149 N(729) | | 1.3 (1.1-1.5) | | 0.01 | | [176] | |  |  |
| XPC |  | insertion/deletion polymorphism (PAT) in intron 9 |  | Increased | | Chinese Han | | 600 BC(92) + 609 N(73) | | 1.52 (1.06-2.18) | | 0.022 | | [300] | |  |  |
| XPC |  | insertion/deletion polymorphism (PAT) in intron 9 |  | Increased | | MA | | 2778 BC() + 3006 N() | | 1.33 (1.03–1.72) | | 0.019 | | [301] | |  |  |
| XPC | NA | insertion/deletion polymorphism (PAT) in intron 9 |  | NA | | MA | | 2210 BC(848) + 2278 N(896) | | 0.96 (0.85-1.08) | | 0.73 | | [189] | |  |  |
| XPC | rs2228000 | c.1496C>T | p.Val499Arg | Increased | | MA | | 1084 BC(87) + 1114 N(54) | | 1.49 (1.06–2.09) | | 0.02 | | [189] | |  |  |
| XPC | rs2228000 | c.1496C>T | p.Val499Arg | Increased | | MA | | 2893 BC() + 3056 N() | | 1.54 (1.21–1.97) | | 0.001 | | [190] | |  |  |
| XPC | rs2228000 | c.1496C>T | p.Ala499Val | Increased | | MA | | 5581 BC() + 6351 N() | | 1.33 (1.06–1.68) | |  | | [191] | |  |  |
| XPC | rs2228000 | c.1496C>T | p.Ala499Val | Increased | | MA | | 3767 BC() + 3907 N() | | 1.12 (1.04–1.21) | | 0.002 | | [192] | |  |  |
| XPC | rs2228000 | c.1496C>T | p.Ala499Val | Increased | | MA | | 2113 BC() + 2249 N() | | 1.82 (1.19–2.79) | | 0.029 | | [193] | |  |  |
| XPC | rs2228001 | c.2815C>A | p.Gln939Lys | Increased | | French | | 51 BC(22) + 45 N(6) | | 7.24 (2.01-26.12) | |  | | [155] | |  |  |
| XPC | rs2228001 | c.2815C>A | p.Gln939Lys | Increased | | MA | | 4927 BC() + 5185 N() | | 1.36 (1.09–1.68) | | 0.001 | | [194] | |  |  |
| XPC | rs2228001 | c.2815C>A | p.Gln939Lys | Increased | | Han-Chinese | | 94 BC(53) + 304 N(185) | | 1.89 (1.21–3.24) | | 0.02 | | [187] | |  |  |
| XPC | rs2228001 | c.2815C>A | p.Gln939Lys | Increased | | MA | | 4828 BC() + 4890 N() | | 1.352 (1.088-1.681) | | 0 | | [195] | |  |  |
| XPC | rs2228001 | c.2815C>A | p.Gln939Lys | Increased | | MA | | 3934 BC() + 4269 N() | | 1.39 (1.08–1.79) | | 0.005 | | [193] | |  |  |
| XPC | rs2228001 | c.2815C>A | p.Gln939Lys | NA | | MA | | 1387 BC(218) + 1362 N(201) | | 1.08 (0.88-1.33) | | 0.004 | | [189] | |  |  |
| XPC | rs2228001 | c.2815C>A | p.Gln939Lys | NA | | MA | | 5064 BC() + 5208 N() | | 1.13 (0.95–1.34) | | 0.171 | | [190] | |  |  |
| XPC | rs2228001 | c.2815C>A | p.Gln939Lys | NA | | UK | | 574 BC(87) + 579 N(84) | | 0.99 (0.69–1.43) | | 0.97 | | [302] | |  |  |
|  | | | | | | | | | | | | | | | |  |  |
| XRCC3 | rs861539 | c.722C>T | p.Thr241Met | Increased | | MA | | 3086 BC() + 3150 N() | | 1.17 (1.00–1.36) | | 0.05 | | [160] | |  |  |
| XRCC3 | rs861539 | c.722C>T | p.Thr241Met | Increased | | Chinese | | 150 BC(15) + 150 N(5) | | 3.22 (1.14–9.11) | | 0.03 | | [199] | |  |  |
| XRCC3 | rs861539 | c.722C>T | p.Thr241Met | Increased | | MA | | 5325 BC() + 7280 N() | | 1.17 (1.03–1.34) | | <0.05 | | [198] | |  |  |
| XRCC3 | rs861539 | c.722C>T | p.Thr241Met | Increased | | MA | | 5667 BC() + 7609 N() | | 1.2 (1.05–1.35) | | 0.005 | | [197] | |  |  |
| XRCC3 | rs861539 | c.722C>T |  | NA | | India | | 211 BC(9) + 244 N(11) | | 0.91 (0.33–2.01) | | 0.88 | | [161] | |  |  |
| XRCC3 | rs861539 | c.722C>T | p.Thr241Met | NA | | MA | | 265 BC() + 427 N() | | 1.11 (0.83–1.49) | |  | | [303] | |  |  |
| XRCC3 | rs861539 | c.722C>T | p.Thr241Met | NA | | French | | 51 BC(28) + 45 N(23) | | 0.62 (0.33-1.18) | |  | | [155] | |  |  |
| XRCC3 | rs1799794 | g.7557A>G |  | Increased | | Northwest Chinese | | 227 BC() + 260 N() | | 2.12 (1.31–3.43) | | 0.002 | | [200] | |  |  |
| XRCC3 | rs861530 | c.194-571A>G |  | Increased | | Northwest Chinese | | 227 BC() + 260 N() | | 2.19 (1.35–3.55) | | 0.001 | | [200] | |  |  |
| BRCA2 | rs11571833 | c.9976A>T | p.Lys3326Ter | Increased | | MA / European descent | | 3591 BC() + 4132 N() | | 1.6 (1.12–2.29) | | 0.01 | | [201] | |  |  |
| NBN | rs1805794 | c.553G>C | p.Glu185Gln | Increased | | MA | | 3542 BC() + 4210 N() | | 1.13 (1.02-1.26) | | 0.022 | | [202] | |  |  |
| FANCI | rs8032440 | c.3651+688C>A |  | Increased | | USA | | 801 BC(104) + 801 N(77) | | 1.4 (1.01-1.93) | | 0.044 | | [172] | |  |  |
| PNKP | rs3739177 | c.199-170G>A |  | Decreased | | USA | | 801 BC(100) + 801 N(127) | | 0.7 (0.52-0.94) | | 0.016 | | [172] | |  |  |
| Poly-ADP-ribosylation | | | | | | | | | | | | | |  | |  |  |
| PARP1 | rs1136410 | c.2285T>C | p.Val762Ala | Increased | | Spanish | | 1150 BC(313) + 1149 N(255) | | 1.24 (1.02-1.51) | |  | | [168] | |  |  |
| PARP1 | rs3219123 | c.2278-39C>T |  | Increased | | USA | | 801 BC(77) + 801 N(100) | | 0.69 (0.50-0.96) | | 0.029 | | [172] | |  |  |
| PARP1 | rs12568297 | g.226544606G>C |  | Increased | | USA | | 801 BC(492) + 801 N(465) | | 1.16 (1.0-1.35) | | 0.044 | | [172] | |  |  |
| PARP2 | rs1713413 | c.640-129C>G |  | Increased | | USA | | 801 BC(112) + 801 N(79) | | 1.51 (1.10-2.07) | | 0.01 | | [172] | |  |  |
| PARP4 | rs2862907 | g.25093650A>C/G |  | Increased | | USA | | 801 BC(326) + 801 N(373) | | 0.79 (0.64-0.97) | | 0.025 | | [172] | |  |  |
| Cell cycle | | | | | | | | | | | | | | | |  |  |
| Tumor suppressor genes | | | | | | | | | | | | | | | |  |  |
| TP53 | NA | intron 3, 16 bp Duplication |  | Decreased | | USA | | 702 BC(636) + 632(618) | | 0.72 (0.55–0.94) | |  | | [183] | |  |  |
| TP53 | rs1042522 | c.215C>G | p.Pro72Arg | Increased | | Bangladeshi | | 102 BC(35) + 140 N(21) | | 3.02 (1.42-6.40) | | <0.01 | | [207] | |  |  |
| TP53 | rs1042522 | c.215C>G | p.Pro72Arg | Increased | | Kashmiri | | 108 BC(86) + 138 N(79) | | 2.9 (1.5–4.5) | | 0.00001 | | [304] | |  |  |
| TP53 | rs1042522 | c.215C>G | p.Pro72Arg | Increased | | Taiwanese | | 127 BC(84) + 427 N(228) | |  | | 0.039 | | [206] | |  |  |
| TP53 | rs1042522 | c.215C>G | p.Pro72Arg | Increased | | MA / East Asians++ | | 2345 BC() + 3200 N() | | 1.32 (1.07–1.62) | | 0.008 | | [205] | |  |  |
| TP53 | rs1042522 | c.215C>G | p.Pro72Arg | Decreased | | Brazilian | | 94 BC(24) + 159 N(60) | | 0.56 (0.32-0.99) | | 0.049 | | [209] | |  |  |
| TP53 | rs1042522 | c.215C>G | p.Pro72Arg | Decreased | | North India | | 200 BC(4) + 265 N(18) | | 0.29 (0.08–1.02) | | 0.053 | | [305] | |  |  |
| TP53 | rs1042522 | c.215C>G | p.Pro72Arg | NA | | Spanish | | 1058 BC(444) + 1138 N(472) | | 1.04 (0.91–1.20) | | 0.5 | | [306] | |  |  |
| TP53 | rs1042522 | c.215C>G | p.Pro72Arg | NA | | Saudi Arabia | | 52 BC(14) + 102 N(28) | | 0.7 (0.5–1.2) | | 0.2 | | [18] | |  |  |
| TP53 | rs1042522 | c.215C>G | p.Pro72Arg | NA | | Taiwanese | | 59 BC(37) + 81 N(60) | | 1.12 (0.40-30.18) | |  | | [60] | |  |  |
| TP53 | rs1042522 | c.215C>G | p.Pro72Arg | NA | | Taiwanese | | 96 BC() + 427 N() | |  | |  | | [206] | |  |  |
| P53 | rs1042522 | c.98C>T/G/A | p.Pro72Leu/Arg/His | Decreased | | Chinese | | 120 BC(37) + 120 BC(55) | | 0.53 (0.31-0.89) | | 0.02 | | [307] | |  |  |
| TP53 | rs17878362 | intron 3 16-bp duplication / microsatellite |  | Decreased | | USA / Caucasians | | 618 BC() + 636 N() | | 0.74 (0.56–0.96) | | 0.032 | | [210] | |  |  |
| P53 | rs121912651 | c.625C>T/G | p.Arg248Trp/Gly | Increased | | North India | | 200 BC(78) + 200 N(48) | | 1.78 (1.20-2.62) | | 0.004 | | [208] | |  |  |
| H19 | rs2839698 | g.5213C>T/G |  | Decreased | | Netherlands | | 177 BC(74) + 204 N(109) | | 0.60 (0.36–0.99) | |  | | [211] | |  |  |
| H19 | rs217727 | g.7158C>T |  | Increased | | Chinese | | 200 BC() + 200 N() | |  | | 0.008 | | [212] | |  |  |
| H19 | rs217727 | g.7158C>T |  | Increased | | Chinese | | 1049 BC(148) + 1399 N(156) | | 1.31 (1.03-1.67) | | 0.029 | | [213] | |  |  |
| RUNX3 | rs760805 | c.481+2142T>A |  | Increased | | Han Chinese | | 368 BC() + 368 N() | | 1.97 (1.44-2.69) | |  | | [214] | |  |  |
| CHEK2 | rs17879961 | c.470T>G/C | p.Ile157Ser/Thr | Decreased | | MA / European descent | | 3591 BC() + 4132 N() | | 0.67 (0.53–0.85) | | 1.0×10^−3^ | | [201] | |  |  |
| TSC2 | rs2073636 | c.482-348A>C |  | Increased | | USA | | 800 BC(525) + 800 N(486) | | 1.2 (1.03-1.39) | | 0.017 | | [172] | |  |  |
| Inhibitors of apoptosis | | | | | | | | | | | | | | | |  |  |
| BIRC5 | rs2071214 | c.385G>A/C/T | p.Glu129Lys/Gln/Ter | Increased | | MA | | 6468 BC() + 7983 N() | | 1.51 (1.04-2.18) | | 0.029 | | [216] | |  |  |
| BIRC5 | rs8073069 | g.4497G>A/C |  | Increased | | MA / Asian | | 6468 BC() + 7983 N() | | 1.37 (1.01-1.84) | | 0.04 | | [216] | |  |  |
| BIRC5 | rs9904341 | g.5091G>A/C/T |  | Increased | | MA / Asian | | 6468 BC() + 7983 N() | | 1.4 (1.13–1.74) | | 0.002 | | [216] | |  |  |
| BIRC5 | rs9904341 | g.5091G>A/C/T |  | Increased | | Asians | | 435 BC() + 546 N() | | 1.77 (1.21–2.59) | | 0.004 | | [217] | |  |  |
| BIRC5 | rs9904341 | g.5091G>A/C/T |  | Increased | | Japanese | | 235 BC(86) + 346 N(87) | | 1.85 (1.27–2.70) | | 0.001 | | [218] | |  |  |
| BIRC5 | rs9904341 | g.5091G>A/C/T |  | Increased | | North India | | 200 BC(32) + 200 N(15) | | 2.61 (1.16–5.87) | | 0.021 | | [219] | |  |  |
| BIRC5 | rs3764383 | g.3575C>G/T |  | Increased | | North India | | 200 BC(27) + 200 N(11) | | 2.54 (1.01–6.35) | | 0.047 | | [219] | |  |  |
| BIRC5 | rs17878467 | g.4881C>G/T |  | Decreased | | MA / Asian | | 6468 BC() + 7983 N() | | 0.69 (0.51–0.92) | | 0.012 | | [216] | |  |  |
| BIRC5 | rs17878467 | g.4881C>G/T |  | Decreased | | North India | | 200 BC(46) + 200 N(64) | | 0.54 (0.30–0.96) | | 0.035 | | [219] | |  |  |
| Others | | | | | | | | | | | | | | | |  |  |
| CCND1 | rs9344 | c.723G>A | p.Pro241= | Increased | | Japanese | | 222 BC(58) + 317 N(53) | | 1.76 (1.09-2.84) | | 0.022 | | [222] | |  |  |
| CCND1 | rs9344 | c.723G>A | p.Pro241= | Increased | | MA | | 2371 BC(608) + 2669 N(619) | | 1.21 (1.01–1.45) | | 0.04 | | [223] | |  |  |
| CCND1 | rs9344 | c.723G>A | p.Pro241= | Increased | | MA* | | 3153 BC() + 3670 N() | | 1.26 (1.13–1.41) | | <0.001 | | [192] | |  |  |
| CCND1 | rs9344 | c.723G>A | p.Pro241= | Increased | | Japanese | | 173 BC() + 0 N() | | 3.67 (1.001–13.453) | | 0.0049 | | [224] | |  |  |
| CCND1 | rs9344 | c.723G>A | p.Pro241= | Increased | | Taiwanese | | 101 BC(9) + 243 N(49) | | 0.34 (0.15-0.76) | | 0.008 | | [225] | |  |  |
| CCND1 | rs9344 | c.723G>A | p.Pro241= | NA | | Non-Hispanic white in LA | | 515 BC(115) + 612 N(132) | | 0.90 (0.60–1.33) | | 0.61 | | [221] | |  |  |
| CCNE1 | rs8102137 | g.30296853T>C |  | Increased | | MA | | 1601 BC() + 1819 N() | |  | |  | | [116] | |  |  |
| CCNE1 | rs8102137 | g.30296853T>C |  | Increased | | MA | |  | |  | |  | | [11] | |  |  |
| CCNE1 | rs8102137 | g.30296853T>C |  | Increased | | Swedish | | 11791 BC() + 52822 N() | | 1.13 (1.09-1.17) | | 1.7x10^-11^ | | [73] | |  |  |
| CCNE1 | rs8102137 | g.30296853T>C |  | Increased | | European decent | | 11791 BC() + 52822 N() | | 1.13 (1.09-1.17) | | 1.7×10^−11^ | | [75] | |  |  |
| CCNE1 | rs8102137 | g.30296853T>C |  | Increased | | MA | | BC() + N() | | 1.13 (1.09–1.17) | |  | | [39] | |  |  |
| POLG | rs3087374 | c.3708G>T | p.Gln1236His | Increased | | USA | | 801 BC(135) + 801 N(104) | | 1.41 (1.06-1.88) | | 0.018 | | [172] | |  |  |
| PSCA | rs2294008 | g.15206C>T |  | Increased | | MA | |  | |  | |  | | [11] | |  |  |
| PSCA | rs2294008 | g.15206C>T |  | Increased | | US and European | | 6667 BC() + 39590 N() | | 1.15 (1.10-1.20) | | 2.14x10^-10^ | | [228] | |  |  |
| PSCA | rs2294008 | g.15206C>T |  | Increased | | Chinese | | 581 BC(309) + 580 N(264) | | 1.38 (1.09–1.75) | | 0.007 | | [229] | |  |  |
| PSCA | rs2294008 | g.15206C>T |  | Increased | | MA | | 5393 BC() + 7324 N() | | 1.11 (1.06–1.17) | | 5.79×10^−5^ | | [230] | |  |  |
| PSCA | rs2294008 | g.15206C>T |  | Increased | | Chinese | | 1210 BC() + 1008 N() | | 1.23 (1.07-1.41) | | 0.003 | | [231] | |  |  |
| PSCA | rs2294008 | g.15206C>T |  | Increased | | MA | | 9617 BC() + 16323 N) | | 1.29 (1.20-1.40) | |  | | [232] | |  |  |
| PSCA | rs2294008 | g.15206C>T |  | Increased | | Japanese | | 539 BC(469) + 5581 N(4495) | | 1.2 | | 0.0092 | | [79] | |  |  |
| PSCA | rs2294008 | g.15206C>T |  | Increased | | Korean | | 411 BC(341) + 1700 N(1286) | | 1.58 (1.15-2.17) | | 0.05 | | [233] | |  |  |
| PSCA | rs2294008 | g.15206C>T |  | Increased | | Chinese Bai | | 87 BC(49) + 72 N(37) | | 1.14 (1.08–1.57) | | 0.031 | | [234] | |  |  |
| PSCA | rs2294008 | g.15206C>T |  | Increased | | Chinese Dai | | 75 BC(53) + 50 N(33) | | 1.33 (1.12–1.70) | | 0.007 | | [234] | |  |  |
| PSCA | rs2294008 | g.15206C>T |  | Increased | | Chinese Han | | 142 BC(76) + 117 N(55) | | 1.34 (1.17–1.69) | | 0.005 | | [234] | |  |  |
| PSCA | rs2294008 | g.15206C>T |  | Increased | | MA | | 14021 BC(6498) + 26871 N(12613) | | 1.16 (1.12-1.20) | | <0.00001 | | [235] | |  |  |
| PSCA | rs2294008 | g.15206C>T |  | Increased | | Swedish | | 6667 BC() + 39590 N() | | 1.15 (1.10–1.20) | | 2.1x10^-10^ | | [73] | |  |  |
| PSCA | rs2294008 | g.15206C>T |  | Increased | | MA | | BC() + N() | | 1.15 (1.10–1.20) | |  | | [39] | |  |  |
| PSCA | rs2294008 | g.15206C>T |  | Increased | | Chinese | | 1050 BC() + 1404 N() | | 1.21 (1.07-1.37) | | 3.004 x 10^-3^ | | [37] | |  |  |
| PSCA | rs2978974 | g.5139G>A |  | Increased | | MA | | BC () + N () | | 1.11 | |  | | [70] | |  |  |
| Cell architecture | | | | | | | | | | | | | | | |  |  |
| CAV1 | rs1049334 | g.40542G>A/T |  | Increased | | MA | | 13778 BC() + 20581 N() | | 1.240 (1.052-1.462) | | 0.011 | | [220] | |  |  |
| CAV1 | rs3807987 | c.195+13091G>A |  | Increased | | Taiwanese | | 375 BC() + 375 N() | |  | | 1.4×10^-14^ | | [237] | |  |  |
| CAV1 | rs7804372 | c.196-4772T>A |  | Increased | | Taiwanese | | 375 BC() + 375 N() | |  | | 6.2×10^-3^ | | [237] | |  |  |
| CAV1 | rs7804372 | c.196-4772T>A |  | Decreased | | MA | | 13778 BC() + 20581 N() | | 0.734 (0.544-0.99) | | 0.043 | | [220] | |  |  |
| CLTA | rs10972786 | c.217+2218G>T |  | Increased | | European | | 3532 BC() + 5120 N() | | 1.27 (1.11-1.45) | | 0.0004 | | [23] | |  |  |
| CLTC | rs7224631 | c.796-301G>A |  | Increased | | European | | 3532 BC() + 5120 N() | | 1.19 (1.06-1.32) | | 0.0023 | | [23] | |  |  |
| DNM1 | rs13285411 | c.1422+1482C>A |  | Increased | | European | | 3532 BC() + 5120 N() | | 0.93 (0.84-1.03) | | 0.1463 | | [23] | |  |  |
| DNM2 | rs4804528 | c.386-173G>T |  | Increased | | European | | 3532 BC() + 5120 N() | | 0.95 (0.89-1.02) | | 0.1437 | | [23] | |  |  |
| KANK2 | rs4804149 | c.2093-229A>G |  | Decreased | | European | | 3532 BC() + 5120 N() | | 0.92 (0.85-0.99) | | 0.0202 | | [23] | |  |  |
| LSP1 | rs907611 | g.4873G>A |  | Increased | | MA / European | | 6901 BC() + 12280 N() | | 1.15 (1.09-1.21) | | 4.11 × 10^-8^ | | [238] | |  |  |
| LSP1 | rs907611 | g.4873G>A |  | NA | | MA / Chinese | | 1050 BC() + 1404 N() | | 0.12 | | 0.129 | | [70] | |  |  |
| RSPH3 | rs12216499 | g.159368524G>C/T |  | Increased | | MA | | BC() + N() | | 1.33 | |  | | [70] | |  |  |
| Cell motricity | | | | | | | | | | | | | | | |  |  |
| DNAL4 | rs738141 | g.2096G>A |  | Increased | | European | | 3532 BC() + 5120 N() | | 1.08 (1.0-1.18) | | 0.0645 | | [23] | |  |  |
| Cell connection | | | | | | | | | | | | | | | |  |  |
| CDH1 | rs16260 | g.4840C>A |  | Increased | | Chinese | | 180 BC(241) + 110 N(92) | | 3.47 (2.00–6.02) | | <0.001 | | [239] | |  |  |
| CDH1 | NA |  |  | Increased | | Chinese | | 180 BC(97) + 110 N(38) | | 2.15 (1.32–3.52) | |  | | [239] | |  |  |
| Extracellular degradation | | | | | | | | | | | | | | | |  |  |
| MMP1 | rs1799750 | g.3471del |  | Increased | | Turkish | | 102 BC(49) + 94 N(22) | | 2.79 (1.53-5.60) | | <0.01 | | [241] | |  |  |
| MMP1 | rs1799750 | g.3471del |  | Increased | | Northern India | | 200 BC(97) + 200 N(60) | | 3.04 (1.71–5.39) | | 0.001 | | [242] | |  |  |
| MMP1 | rs1799750 | g.3471del |  | Decreased | | Polish | | 241 BC(173) + 199 N(160) | | 0.62 (0.39–0.98) | | 0.042 | | [243] | |  |  |
| MMP1 | rs1799750 | g.3471del |  | Increased | | MA | | 1103 BC() + 1053 N() | | 1.44 (1.05-1.97) | | 0.022 | | [244] | |  |  |
| MMP1 | rs1799750 | g.3471del |  | Decreased | | MA | | 1096 BC() + 1048 N() | | 0.57 (0.36–0.93) | | 0.001 | | [245] | |  |  |
| MMP2 | rs243865 | g.3726C>T |  | Increased | | Northern India | | 200 BC(98) + 200 N(69) | | 2.61 (1.63–4.17) | | <0.001 | | [246] | |  |  |
| MMP2 | rs243865 | g.3726C>T |  | NA | | Polish | | 241 BC(98) + 199 N(79) | | 1.05 (0.71–1.55) | | 0.799 | | [243] | |  |  |
| MMP2 | rs243865 | g.3726C>T |  | Increased | | MA | | 839 BC() + 775 N() | | 2.1 (1.38-3.10) | | 0 | | [244] | |  |  |
| MMP2 | rs243865 | g.3726C>T |  | Decreased | | MA / Asian | | 1020 BC() + 960 N() | | 0.41 (0.18–0.94) | | 0.195 | | [245] | |  |  |
| MMP7 | rs11568818 | g.102401661T>A/C |  | Increased | | Northern India | | 200 BC(52) + 200 N(30) | | 2.38 (1.30–4.34) | | 0.005 | | [242] | |  |  |
| MMP7 | rs11568818 | g.102401661T>A/C |  | Decreased | | MA | | 440 BC() + 399 N() | | 0.81 (0.66–0.98) | | 0.325 | | [245] | |  |  |
| MMP7 | rs11568818 | g.102401661T>A/C |  | NA | | Taiwan | | 375 BC(41) + 375 N(47) | | 0.84 (0.54-1.32) | | 0.496 | | [247] | |  |  |
| MMP7 | rs11568818 | g.102401661T>A/C |  | NA | | Chinese | | 355 BC(65) + 435 N(61) | | 1.21 (0.88–1.67) | | 0.231 | | [308] | |  |  |
| MMP11 | rs28382575 | c.1425T>C | p.Pro475= | Increased | | Taiwan | | 431 BC(26) + 650 N(19) | | 2.045 (1.088-3.843) | | 0.026 | | [248] | |  |  |
| Metabolic | | | | | | | | | | | | | | | |  |  |
| Alcohol metabolism | | | | | | | | | | | | | | | |  |  |
| AKR1C3 | rs12529 | c.15C>G/T | p.His5Gln/= | Decreased | | Turkish | | 250 BC(18) + 250 N(64) | | 0.352 (0.175-0.708) | | 0.003 | | [249] | |  |  |
| COMT | rs4680 | c.472G>A | p.Val158Met | Decreased | | South Egypt | | 314 BC() + 352 N() | | 0.63 (0.44-0.91) | | 0.004 | | [250] | |  |  |
| COMT | rs4680 | c.472G>A | p.Val158Met | Increased | | MA | | 3285 BC() + 3594 N() | | 0.826 (0.717-0.951) | | 0.231 | | [251] | |  |  |
| COMT | rs4680 | c.472G>A | p.Val158Met | NA | | French / Caucasian | | 51 BC(42) + 45 N(34) | | 1.41 (0.51-3.94) | |  | | [19] | |  |  |
| Solute carriers | | | | | | | | | | | | | | | |  |  |
| SLC14A | rs17674580 | c.148-354C>A |  | Increased | | MA | | 6024 BC() + 49110 N() | | 1.17 (1.11–1.22) | | 7.6×10^−11^ | | [253] | |  |  |
| SLC14A | rs17674580 | c.148-354C>A |  | Increased | | MA | | BC() + N() | | 1.17 (1.11–1.22) | |  | | [39] | |  |  |
| SLC14A1 | rs1058396 | c.1006G>A | p.Asp336Asn | Increased | | MA | | BC () + N () | | 1.14 | |  | | [70] | |  |  |
| SLC14A1 | rs10775480 | c.831+669T>A |  | Increased | | MA | |  | |  | |  | | [11] | |  |  |
| SLC14A1 | rs10775480 | c.831+669T>A |  | Increased | | European decent | | 5883 BC(5801) + 8277 N(7894) | | 1.16 (1.10-1.22) | | 8.9x10^−9^ | | [254] | |  |  |
| SLC14A1 | rs10853535 | c.831+934C>A |  | Increased | | European decent | | 5883 BC(4499) + 8277 N(6068) | | 1.15 (1.08-1.22) | | 4.41x10^−6^ | | [254] | |  |  |
| SLC14A1 | rs10853535 | c.831+934C>A |  | Increased | | MA | | BC() + N() | | 1.16 | |  | | [70] | |  |  |
| SLC14A1 | rs17674580 | c.148-354C>A |  | Increased | | Japanese | | 539 BC(91) + 5581 N(607) | | 1.54 | | 1.8×10^−4^ | | [79] | |  |  |
| SLC14A1 | rs17674580 | c.148-354C>A |  | Increased | | India | | 225 BC(38) + 240 N(19) | | 3.01 (1.37–6.63) | | 0.006 | | [117] | |  |  |
| SLC14A1 | rs17674580 | c.148-354C>A |  | Increased | | Chinese | | 1050 BC() + 1404 N() | | 1.65 (1.38-1.99) | | 8.507 x 10^-8^ | | [37] | |  |  |
| SLC14A1 | rs7238033 | c.831+353T>C |  | Increased | | European decent | | 5883 BC(3992) + 8277 N(4977) | | 1.20 (1.13-1.28) | | 8.7x10^-9^ | | [254] | |  |  |
| SLC14A1 | rs7238033 | c.831+353T>C |  | Increased | | MA | | BC () + N () | | 1.2 | |  | | [70] | |  |  |
| SLC14A1 | rs7238033 | c.831+353T>C |  | Increased | | MA | | BC() + N() | | 1.20 (1.13–1.28) | |  | | [39] | |  |  |
| SLC2A1 | rs1385129 | c.45C>T | p.Ala15= | Decreased | | Chinese | | 314 BC(12) + 204 N(39) | |  | | <0.001 | | [255] | |  |  |
| SLC39A11 | rs11871756 | c.692+6545G>C |  | Increased | | MA | | BC() + N() = 4590 | | 1.43 (1.24–1.63) | | 0.0002 | | [256] | |  |  |
| SLC39A11 | rs11077654 | c.306+21183T>G |  | Increased | | MA | | BC() + N() = 4576 | | 0.76 (0.68–0.85) | | 0.001 | | [256] | |  |  |
| SLC39A11 | rs9913017 | c.306+16675T>G |  | Increased | | MA | | BC() + N() = 4548 | | 0.76 (0.68–0.85) | | 0.002 | | [256] | |  |  |
| SLC39A11 | rs4969054 | c.306+15222C>G |  | Increased | | MA | | BC() + N() = 4416 | | 0.78 (0.69–0.88) | | 0.02 | | [256] | |  |  |
| SLCO1B1 | rs2306283 | c.388A>C/G/T | p.Asn130His/Asp/Tyr | Increased | | Japanese | | 237 BC(213) + 246 N(208) | | 2.01 (1.14–3.56) | | 0.016 | | [257] | |  |  |
| Mitochondrial folate metabolic enzymes | | | | | | | | | | | | | | | |  |  |
| MTHFD2 | rs1667627 | c.101+3323C>T |  | Increased | | USA | | 832 BC(152) + 1191 N(183) | | 1.7 (1.3-2.3) | | <0.004 | | [89] | |  |  |
| MTHFR | rs1801131 | c.1286A>C | p.Glu429Ala | Increased | | Iranian | | 158 BC(25) +316 N(23) | | 3.51 (2.42–5.64) | | 0.001 | | [259] | |  |  |
| MTHFR | rs1801131 | c.1286A>C | p.Glu429Ala | NA | | Turkish | |  | |  | | 0.278 | | [263] | |  |  |
| MTHFR | rs1801131 | c.1286A>C | p.Glu429Ala | Increased | | MA | | BC() + N() | | 1.29 (1.08-1.55) | |  | | [260] | |  |  |
| MTHFR | rs1801131 | c.1286A>C | p.Glu429Ala | Increased | | MA / Asian and African | | 915 BC() + 1103 N() | | 1.29 (1.00–1.66) | | 0.048 | | [261] | |  |  |
| MTHFR | rs1801131 | c.1286A>C | p.Glu429Ala | Increased | | Tunisian | | 111 BC(47) + 131 N(37) | | 1.86 (1.04-3.33) | | 0.03 | | [262] | |  |  |
| MTHFR | rs1801131 | c.1286A>C | p.Glu429Ala | Increased | | Tunisian | | 185 BC(78) + 191 N(60) | | 1.62 (1.03- 2.55) | | 0.03 | | [267] | |  |  |
| MTHFR | rs1476413 | c.1632+35G>C |  | Decreased | | USA | | 219 BC(10) + 273 N(27) | | 0.40 (0.18-0.88) | |  | | [266] | |  |  |
| MTHFR | rs1801131 | c.1286A>C | p.Glu429Ala | NA | | Chinese | | 312 BC(6) + 325 N(7) | | 0.90 (0.30-2.72) | | >0.05 | | [264] | |  |  |
| MTHFR | rs1801133 | c.665C>T | p.Ala222Val | Increased | | Chinese | | 312 BC(61) + 325 N(42) | | 2.00 (1.23–3.25) | | 0.017 | | [264] | |  |  |
| MTHFR | rs1801133 | c.665C>T | p.Ala222Val | Increased | | Turkish | |  | |  | | 0.036 | | [263] | |  |  |
| MTHFR | rs1801133 | c.665C>T | p.Ala222Val | Increased | | MA | | BC() + N() | | 0.79 (0.64-0.97) | |  | | [260] | |  |  |
| MTHFR | rs1801133 | c.665C>T | p.Ala222Val | Increased | | MA / Asian | | 859 BC() + 1191 N() | | 1.38 (1.13–1.69) | | 0.002 | | [261] | |  |  |
| MTHFR | rs1801133 | c.665C>T | p.Ala222Val | Increased | | Saudi arabia | | 52 BC(5) + 102 N(0) | | 3.6 (1.8–7.1) | | 0.0002 | | [18] | |  |  |
| MTHFR | rs1801133 | c.665C>T | p.Ala222Val | NA | | Iranian | | 158 BC(17) +316 N(30) | | 1.28 (0.81–1.42) | | 0.74 | | [259] | |  |  |
| MTHFR | rs1801133 | c.665C>T | p.Ala222Val | NA | | MA | | 3570 BC() + 3926 N() | | 0.998 (0.869–1.145) | | 0.117 | | [309] | |  |  |
| MTHFR | rs1801133 | c.665C>T | p.Ala222Val | NA | | MA | | 3570 BC() + 3926 N() | | 1.00 (0.87-1.15) | | 0.975 | | [310] | |  |  |
| MTHFR | rs1801133 | c.665C>T | p.Ala222Val | NA | | Tunisian | | 111 BC(78) + 131 N(90) | | 1.04 (0.71-1.51) | | 0.86 | | [262] | |  |  |
| MTHFR | rs1801133 | c.665C>T | p.Ala222Val | NA | | Tunisian | | 185 BC(98) + 191 N(110) | | 0.83 (0.54-1.27) | | 0.42 | | [267] | |  |  |
| MTR | rs1805087 | c.2756A>G | p.Asp919Gly | Increased | | Tunisian | | 111 BC(61) + 131 N(45) | | 2.33 (1.34-4.06) | | 0.001 | | [262] | |  |  |
| MTR | rs1805087 | c.2756A>G | p.Asp919Gly | Increased | | Tunisian | | 185 BC(80) + 191 N(52) | | 2.13 (1.35-3.37) | | 0.0008 | | [267] | |  |  |
| MTRR | rs1801394 | c.66A>G | p.Ile22Met | Increased | | Saudi arabia | | 52 BC(20) + 102 N(10) | | 4.1 (2.5–6.7) | | <0.0001 | | [18] | |  |  |
| MTRR | rs1801394 | c.66A>G | p.Ile22Met | NA | | Tunisian | | 185 BC(126) + 191 N(114) | | 1.44 (0.92-2.25) | | 0.11 | | [267] | |  |  |
| Metabolism of water-soluble vitamins | | | | | | | | | | | | | | | |  |  |
| NAMPT | rs61330082 | g.105926865G>A/T |  | Increased | | Chinese | | 407 BC(105) + 316 N(63) | | 1.24 (1.00–1.52) | | 0.047 | | [268] | |  |  |
| NMNAT2 | rs4652795 | c.86-3254G>A |  | Increased | | European | | 3532 BC() + 5120 N() | | 0.92 (0.86-0.98) | | 0.0099 | | [23] | |  |  |
| NMNAT3 | rs7636269 | c.-103+5379A>G |  | Increased | | European | | 3532 BC() + 5120 N() | | 1.12 (1.05-1.2) | | 0.0004 | | [23] | |  |  |
| NMRK2 | rs2304191 | c.-215+56T>C |  | Increased | | European | | 3532 BC() + 5120 N() | | 1.11 (1.01-1.23) | | 0.0355 | | [23] | |  |  |
| Others | | | | | | | | | | | | | | | |  |  |
| GPX1 | rs968601828 | c.593A>C | p.Gln198Pro | Increased | | Japanese | | 213 BC(47) + 209 N(22) | | 2.63 (1.45-4.75) | | 0.001 | | [311] | |  |  |
| GPX1 | rs968601828 | c.593A>C | p.Gln198Pro | Increased | | MA | | 1092 BC() + 1179 N() | | 1.876 (1.011–3.480) | | <0.001 | | [273] | |  |  |
| GPX1 | rs968601828 | c.593A>C | p.Gln198Pro | NA | | Morocco | | 32 BC() + 40 N() | |  | | 0.435 | | [312] | |  |  |
| GPX1 | rs1050450 | c.599C>T | p.Pro200Leu | Increased | | MA | | 1092 BC() + 1179 N() | | 2.111 (1.020-4.368) | | <0.001 | | [313] | |  |  |
| GPX1 | rs1050450 | c.599C>T | p.Pro200Leu | Increased | | Turkish | | 157 BC(30) + 224 N(20) | | 1.67 (1.17–2.40) | | 0.005 | | [314] | |  |  |
| GPX1 | rs1050450 | c.599C>T | p.Pro200Leu | NA | | Egypt | | 625 BC(35) + 626 N(38) | | 1.28 (0.75 - 2.19) | |  | | [59] | |  |  |
| NOS3 | rs1799983 | c.894T>A | p.Asp298Glu | Increased | | Turkish | | 66 BC() + 88 N() | |  | | <0.001 | | [315] | |  |  |
| NOS3 | rs1799983 | c.894T>A/G | p.Asp298Glu | Increased | | Turkish | | 75 BC(59) + 143 N(75) | | 0.324 (0.106-0.990) | | 0.026 | | [316] | |  |  |
| NOS3 | rs1799983 | c.894T>A | p.Asp298Glu | Increased | | Sweden | | 359 BC(40) + 164 N(8) | | 2.88 (1.21–6.85) | | 0.017 | | [317] | |  |  |
| PON1 | rs662 | c.575A>G | p.Gln192Arg | Increased | | Turkish | | 76 BC(15) + 135 N(14) | | 2.125 (0.963-4.686) | | 0.058 | | [318] | |  |  |
| POR | rs1057868 | c.1508C>T | p.Ala503Val | Decreased | | Chinese | | 1050 BC(118) + 1404 N(207) | | 0.727 (0.570–0.927) | | 0.008 | | [319] | |  |  |
| PXYLP1 | rs3210458 | c.1026C>G/T | p.Phe342Leu/= | Increased | | European | | 3532 BC() + 5120 N() | | 1.12 (1.00-1.25) | | 0.0421 | | [23] | |  |  |
| QPRT | rs3862476 | g.29687360C>A/T |  | Increased | | European | | 3532 BC() + 5120 N() | | 1.19 (1.04-1.35) | | 0.0087 | | [23] | |  |  |
| TYMS | rs34743033 | c.*34+112_*34+185delins |  | Decreased | | Tunisian | | 185 BC(5) +191 N(25) | | 0.12 (0.03–0.40) | | 0.0001 | | [267] | |  |  |
| NQO1 | rs1437135 | c.7+2508T>C |  | Increased | | European | | 3532 BC() + 5120 N() | | 0.91 (0.84-0.99) | | 0.0275 | | [23] | |  |  |
| NQO1 | rs1800566 | c.559C>T | p.Pro187Ser | Increased | | USA / Caucasian | | 265 BC(90) + 261 N(76) | | 1.51 (1.01–2.25) | | 0.04 | | [320] | |  |  |
|  |  |  |  |  | |  | |  | |  | |  | |  | |  |  |
| NQO1 | rs1800566 | c.559C>T | p.Pro187Ser | Increased | | India | | 200 BC(95) + 200 N(72) | | 1.6 (1.07–2.40) | | 0.02 | | [321] | |  |  |
| NQO1 | rs1800566 | c.559C>T | p.Pro187Ser | Increased | | MA | | 2286 BC() + 2294 N() | | 2.54 (1.188-5.469) | | 0.016 | | [321] | |  |  |
| NQO1 | rs1800566 | c.559C>T | p.Pro187Ser | Increased | | MA* | | 4298 BC(2735) + 4275 N(2782) | | 1.43 (1.08–1.90) | | 0.012 | | [322] | |  |  |
| NQO1 | rs1800566 | c.559C>T | p.Pro187Ser | Increased | | MA | | 4298 BC() + 4275 N() | | 1.43 (1.08–1.90) | | 0.009 | | [323] | |  |  |
| NQO1 | rs1800566 | c.559C>T | p.Pro187Ser | Increased | | MA* | | 3041 BC() + 3128 N() | | 1.15 (1.01-1.30) | | 0.03 | | [324] | |  |  |
| NQO1 | rs1800566 | c.559C>T | p.Pro187Ser | NA | | Lebanese | | 54 BC() + 106 N() | | 0.72 (0.08-6.6) | | 0.77 | | [28] | |  |  |
| NQO1 | rs1800566 | c.559C>T | p.Pro187Ser | NA | | MA | | 2937 BC() + 3008 N() | | 1.12 (0.99–1.26) | | 0.069 | | [325] | |  |  |
| NQO1 | rs1800566 | c.559C>T | p.Pro187Ser | NA | | MA | | 2661 BC() + 2738 N() | | 1.08 (0.96–1.21) | | 0.2 | | [326] | |  |  |
| Others | | | | | | | | | | | | | | | |  |  |
| ACP6 | rs1344 | c.1239C>T | p.His413= | Increased | | European | | 3532 BC() + 5120 N() | | 1.11 (1.04-1.18) | | 0.0017 | | [23] | |  |  |
| CBX6 / APOBEC3A | rs1014971 | g.39332623C>G/T |  | Increased | | MA / Chinese | | 1050 BC() + 1404 N() | | 1.14 | | 0.05 | | [70] | |  |  |
| APOBEC3A | rs1014971 | g.39332623C>G/T |  | Increased | | European decent | | 11806 BC() + 52609 N() | | 0.88 (0.85–0.91) | | 8×10^−12^ | | [75] | |  |  |
| APOBEC3A | rs1014971 | g.39332623C>G/T |  | Increased | | MA | |  | |  | |  | | [11] | |  |  |
| APOBEC3A | rs1014971 | g.39332623C>G/T |  | Increased | | Non-latino white | | 489 BC(460) + 578 N(536) | | 1.24 (1.02–1.52) | | 0.032 | | [327] | |  |  |
| APOBEC3A | rs1014971 | g.39332623C>G/T |  | Increased | | Swedish | | 11806 BC() + 52609 N() | | 0.88 (0.85–0.91) | | 8.4x10^-12^ | | [73] | |  |  |
| ARRB1 | rs667791 | c.52-1009C>T |  | Increased | | European | | 3532 BC() + 5120 N() | | 1.11 (1.04-1.19) | | 0.0014 | | [23] | |  |  |
| C12orf73 | rs2700505 | c.108+721A>G |  | Increased | | USA | | 801 BC(452) + 801 N(411) | | 1.17 (1.01-1.37) | | 0.039 | | [172] | |  |  |
| C12orf73 | rs812498 | g.104354799T>A/C |  | Increased | | USA | | 801 BC(313) + 801 N(273) | | 1.24 (1.0-1.53) | | 0.046 | | [172] | |  |  |
| CASC11 | rs9642880 | g.128718068G>A/T |  | Increased | | French | | 231 BC(53) +261 N(50) | | 1.72 (1.1-2.8) | | 0.028 | | [328] | |  |  |
| CASC11 | rs9642880 | g.128718068G>A/T |  | Increased | | Chinese | | 1210 BC() + 1008 N() | | 1.14 (1.0-1.29) | | 0.048 | | [231] | |  |  |
| CASC11 | rs9642880 | g.128718068G>A/T |  | Increased | | Japanese | | 539 BC(289) + 5581 N(2656) | | 1.15 | | 0.039 | | [79] | |  |  |
| CASC11 | rs9642880 | g.128718068G>A/T |  | Increased | | MA | | 23084 BC() + 97164 N() | | 1.18 (1.14-1.22) | | <0.05 | | [329] | |  |  |
| CASC11 | rs9642880 | g.128718068G>A/T |  | Increased | | MA / European | | 3855 BC() + 37985 N() | | 1.22 (1.15–1.29) | | 9.34x10^-12^ | | [330] | |  |  |
| CASC11 | rs9642880 | g.128718068G>A/T |  | Increased | | German | | 212 BC() + 194 N() | | 1.362 (1.023-1.813) | | 0.029 | | [331] | |  |  |
| CASC11 | rs9642880 | g.128718068G>A/T |  | Increased | | Non-Hispanic white and Chinese | | 1050 BC() +1137 N() | | 1.32 (1.16–1.50) | | 0.000024 | | [332] | |  |  |
| CASC11 | rs9642880 | g.128718068G>A/T |  | Increased | | MA | | BC() + N() | | 1.22 (1.15-1.29) | |  | | [39] | |  |  |
| CASC11 | rs9642880 | g.128718068G>A/T |  | Increased | | MA / Chinese | | 1050 BC() + 1404 N() | | 1.24 | | 0.01 | | [70] | |  |  |
| CASC11 | rs9642880 | g.128718068G>A/T |  | Increased | | Chinese | | 415 BC(149) + 465 N(223) | | 1.65 (1.25–2.17) | | 0.0003 | | [118] | |  |  |
| CASC11 | rs9642880 | g.128718068G>A/T |  | Increased | | Chinese | | 1050 BC() + 1404 N() | | 1.24 (1.09-1.30) | | 9.763 x 10^-3^ | | [37] | |  |  |
| CASC11 | rs9642880 | g.129324232T>C/G |  | Increased | | Chinese | | 581 BC() + 1561 N() | | 1.17 (1.06-1.30) | | <0.001 | | [333] | |  |  |
| CASC11 | rs10094872 | g.128719884A>T |  | Increased | | MA | |  | |  | |  | | [11] | |  |  |
| CDKAL1 | rs4510656 | c.517+7823C>A |  | Increased | | MA / European | | 7697 BC() + 13110 N() | | 0.89 (0.85-0.93) | | 6.98 × 10^-7^ | | [238] | |  |  |
| CDKAL1 | rs4510656 | c.517+7823C>A |  | Increased | | MA | | BC() + N() | | 1.12 | |  | | [70] | |  |  |
| CDKAL1 | rs7747724 | c.469-7511T>C |  | Increased | | MA | |  | |  | |  | | [11] | |  |  |
| CWC27 | rs2042329 | c.42+2737T>A |  | Increased | | Chinese | | 3385 BC(83) + 4630 N(44) | | 1.40 (1.27–1.55) | | 4.61×10^-11^ | | [334] | |  |  |
| DDX20 | rs197414 | c.2077C>A/T | p.Arg693Ser/Cys | Increased | | USA / Caucasian | | 746 BC(23) + 746 N(8) | | 2.50 (1.08–5.78) | | 0.03 | | [335] | |  |  |
| FBXO5 | rs9479476 | c.103+2450A>G |  | Decreased | | European | | 3532 BC() + 5120 N() | | 0.83 (0.75-0.93) | | 0.001 | | [23] | |  |  |
| FOXF2 | rs1711973 | g.1402537T>A/C/G |  | Increased | | MA | | BC() + N() | | 1.34 | |  | | [70] | |  |  |
| GBF1 | rs1057050 | g.104142294G>A/C |  | ? | | European | | 3532 BC() + 5120 N() | | 0.9 (0.78-1.04) | | 0.1673 | | [23] | |  |  |
| HOTAIR | rs874945 | g.54355451C>T |  | Increased | | Chinese | | 1050 BC(399) + 1407 N(465) | | 1.23 (1.04-1.46) | | 0.014 | | [336] | |  |  |
| HOXB5 | rs9299 | g.46669430C>A/T |  | Increased | | Chinese | | 391 BC(308) + 391 N(278) | | 1.487 (1.07-2.06) | | 0.017 | | [337] | |  |  |
| JAG1 | rs4813953 | g.10991138T>C |  | Increased | | MA | | BC() + N() | | 1.16 | |  | | [70] | |  |  |
| JAG1 | rs4813953 | g.10991138T>C |  | Increased | | Chinese | | 581 BC() + 1561 N() | | 1.09 (1.02–1.17) | | 0.016 | | [333] | |  |  |
| JAG1 | rs6104690 | g.10988099G>A/C/T |  | Increased | | MA | | BC() + N() | | 1.12 | |  | | [70] | |  |  |
| JAG1 | rs6104690 | g.10988099G>A/C/T |  | Increased | | European | | 15058 BC() + 286270 N() | |  | | 2.19×10^−11^ | | [238] | |  |  |
| JAG1 | rs6104690 | g.10988099G>A/C/T |  | Increased | | MA / European | | 7678 BC() + 13088 N() | | 0.89 (0.85-0.93) | | 7.13 × 10^-7^ | | [338] | |  |  |
| JAG1 | rs62185668 | g.10961935C>A/T |  | Increased | | MA | | 6936 BC() + 99 682 N() | | 1.19 (1.13-1.26) | | 1.5x10^-11^ | | [339] | |  |  |
| LOC105379243 | rs2645446 | g.11649069G>A/C/T |  | Increased | | USA | | 801 BC(538) + 801 N(500) | | 1.25 (1.01-1.55) | | 0.037 | | [172] | |  |  |
| LRP6 | rs10743980 | c.55+6820A>T |  | Decreased | | USA / Caucasian | | 803 BC(131) + 803 N(156) | | 0.76 (0.58- 0.99) | | 0.039 | | [340] | |  |  |
| LRRC43 | rs4758680 | g.122655352T>A |  | Increased | | Chinese | | 294 BC(104) + 478 N(132) | | 1.43 (1.05-1.96) | | 0.02 | | [341] | |  |  |
| LZIC | rs1220398 | c.336+481T>C |  | Increased | | European | | 3532 BC() + 5120 N() | | 0.89 (0.81-0.98) | | 0.0169 | | [23] | |  |  |
| MAP2K4 | rs4791489 | g.129142C>T |  | Increased | | Spanish | | 1047 BC() + 988 N() | | 0.49 | | 0.03 | | [101] | |  |  |
| MAP3K7 | rs812606 | c.1276-857T>G |  | Increased | | Spanish | | 1047 BC() + 988 N() | | 2.68 | | 0.005 | | [101] | |  |  |
| MCF2L | rs4907479 | c.80-9969G>A |  | Increased | | European | | 15058 BC() + 286270 N() | |  | | 3.3×10^−10^ | | [338] | |  |  |
| MDM2 | rs937282 | g.4827C>G |  | Increased | | Chinese | | 234 BC() + 253 N() | | 2.45 (1.02-5.72) | | 0.152 | | [342] | |  |  |
| MDM2 | rs2279744 | c.14+309T>G |  | Increased | | MA / Caucasian | | 972 BC() + 1012 N() | | 1.41 (1.10-1.81) | | 0.006 | | [343] | |  |  |
| MDM2 | rs2279744 | c.14+309T>G |  | NA | | MA | | 1254 BC() + 1787 N() | | 1.06 (0.89–1.27) | | 0.5 | | [344] | |  |  |
| MDM2 | rs2279744 | c.14+309T>G |  | NA | | German / Caucasian | | 224 BC(149) + 140 N(89) | | 1.138 (0.732–1.771) | | 0.573 | | [345] | |  |  |
| MIF | rs1016305644 | g.4925A>C |  | Decreased | | Southeast China | | 325 BC(119) + 345 N(170) | | 0.57 (0.41–0.79) | | <0.001 | | [346] | |  |  |
| MIR143HG / CARMEN | rs353293 | g.148807226C>T |  | Decreased | | Chinese | | 333 BC(65) + 536 N(148) | | 0.64 (0.46–0.88) | | 0.006 | | [347] | |  |  |
| MIR146A | rs2910164 | g.159912418C>G |  | NA | | India | | 212 BC(85) + 250 N(115) | | 0.79 (0.54-1.15) | | 0.221 | | [348] | |  |  |
| miR146a | rs2910164 | g.159912418C>G |  | Decreased | | Chinese | | 1019 BC () + 1182 N() | | 0.8 (0.71–0.90) | | 2.920×10^−4^ | | [349] | |  |  |
| MIR196A2 | rs11614913 | g.54385599C>T |  | Decreased | | Southwest China | | 159 BC(66) + 298 N(166) | | 0.56 (0.38–0.83) | | 0.004 | | [350] | |  |  |
| MIR196A2 | rs11614913 | g.54385599C>T |  | NA | | India | | 212 BC(136) + 250 N(141) | | 1.36 (0.93-1.98) | | 0.11 | | [348] | |  |  |
| MYNN | rs10936599 | c.18C>G/T | p.His6Gln/= | Increased | | MA / European | | 7691 BC() + 13108 N() | | 0.85 (0.81-0.90) | | 4.53 × 10^-9^ | | [238] | |  |  |
| MYNN | rs10936599 | c.18C>G/T | p.His6Gln/= | Increased | | Chinese | | 1050 BC() + 1404 N() | | 1.22 (1.09-1.37) | | 7.179 x 10^-4^ | | [37] | |  |  |
| NADSYN1 | rs4945007 | g.71220184C>T |  | Increased | | European | | 3532 BC() + 5120 N() | | 1.1 (0.96-1.25) | | 0.1555 | | [23] | |  |  |
| NAMPTP1 | rs2505568 | g.1929A>T/C |  | Decreased | | Chinese | | 407 BC(37) + 316 N(77) | | 0.31 (0.20–0.47) | | <0.0001 | | [268] | |  |  |
| PAG1 | rs5003154 | c.-233-4548A>T |  | Increased | | MA | |  | |  | |  | | [11] | |  |  |
| PDCD6 | rs4957014 | c.163+15127T>G |  | Decreased | | Chinese | | 332 BC(125) + 509 N(232) | | 0.72 (0.54–0.95) | | 0.023 | | [351] | |  |  |
| PDCD6 | rs3756712 | c.367+2221A>C |  | Decreased | | Chinese | | 332 BC(17) + 509 N(47) | | 0.53 (0.30–0.94) | | 0.024 | | [351] | |  |  |
| PINX1 | rs1469557 | g.10706801C>T |  | Decreased | | USA / Caucasian | | 803 BC(418) + 803 N(271) | | 0.76 (0.61–0.94) | | 0.0077 | | [352] | |  |  |
| PINX1 | rs17152584 | c.302-2097C>T |  | Decreased | | USA / Caucasian | | 803 BC() + 803 N() | | 0.76 (0.60–0.96) | | 0.0186 | | [352] | |  |  |
| PINX1 | rs6995541 | c.471+6443T>C |  | Decreased | | USA / Caucasian | | 803 BC() + 803 N() | | 0.82 (0.70–0.97) | | 0.0199 | | [352] | |  |  |
| PINX1 | rs9657541 | c.472-19738G>C |  | Decreased | | USA / Caucasian | | 803 BC() + 803 N() | | 0.78 (0.63–0.97) | | 0.022 | | [352] | |  |  |
| PINX1 | rs11250080 | c.471+3236A>G |  | Decreased | | USA / Caucasian | | 803 BC() + 803 N() | | 0.66 (0.46–0.95) | | 0.025 | | [352] | |  |  |
| PINX1 | rs7826180 | c.472-4418C>T |  | Decreased | | USA / Caucasian | | 803 BC() + 803 N() | | 0.60 (0.38–0.96) | | 0.0301 | | [352] | |  |  |
| PINX1 | rs2409655 | c.471+1871T>C |  | Increased | | USA / Caucasian | | 803 BC() + 803 N() | | 1.24 (1.01–1.52) | | 0.0392 | | [352] | |  |  |
| POT1 | rs4360236 | c.124+5581G>A |  | Decreased | | USA / Caucasian | | 803 BC() + 803 N() | | 0.75 (0.58–0.97) | | 0.0261 | | [352] | |  |  |
| PTGS2 (COX-2) | rs20417 | g.4239G>C/A |  | Increased | | Taiwanese | | 375 BC(89) + 375 N(60) | | 1.63 (1.13-2.35) | | 0.0102 | | [353] | |  |  |
| PTGS2 (COX-2) | rs20417 | g.4239G>C/A |  | Increased | | North India | | 212 BC(84) + 250 N(73) | | 1.9 (1.20–2.69) | | 0.004 | | [354] | |  |  |
| PTGS2 (COX-2) | rs20432 | c.640-275T>G |  | Increased | | Spanish | | 1047 BC() + 988 N() | | 0.28 | | 0.003 | | [101] | |  |  |
| PTGS2 (COX-2) | rs5275 | g.11502T>C/A |  | Decreased | | USA / Caucasian | | 635 BC(344) + 635 N(397) | | 0.68 (0.54-0.87) | | 0.002 | | [355] | |  |  |
| RAG1 | rs2227973 | c.2459A>G | p.Lys820Arg | Increased | | USA | | 702 BC(605) + 632 N(595) | | 1.32 (1.00–1.73) | |  | | [183] | |  |  |
| RGS4 | rs10759 | g.12956G>A/T |  | Decreased | | USA / Caucasian | | 803 BC() + 803 N() | | 0.77 (0.66–0.90) | | 0.00073 | | [356] | |  |  |
| RGS6 | rs2074647 | g.635362G>A |  | Decreased | | USA / Caucasian | | 477 BC() + 446 N() | | 0.66 (0.46-0.95) | |  | | [357] | |  |  |
| RPL7P35 | rs7918064 | g.112314007T>A/C |  | significant protective effect | | European | | 3532 BC() + 5120 N() | | 0.9 (0.84-0.97) | | 0.0073 | | [23] | |  |  |
| SFRP1 | rs3242 | g.41119554G>A/C |  | Increased | | German / Caucasian | | 403 BC(257) + 332 N(185) | | 1.399 (1.039-1.882) | | 0.028 | | [358] | |  |  |
| SH3GL2 | rs2209426 | c.187+4808G>A |  | Increased | | European | | 3532 BC() + 5120 N() | | 0.87 (0.80-0.95) | | 0.002 | | [23] | |  |  |
| SLAMF1 | rs1061217 | g.160580015A>C |  | Increased | | Spanish | | 1047 BC() + 988 N() | | 1.73 | | 0.077 | | [101] | |  |  |
| SMAD3 | rs12324036 | c.206+18792T>C |  | Decreased | | USA / Caucasian | | 801 BC(361) + 801 N(408) | | 0.69 (0.54–0.88) | | 2.6x10^-3^ | | [126] | |  |  |
| SULT1A1 | rs9282861 | c.638G>A | p.Arg213His | Increased | | MA** | | 2036 BC() + 2273 N() | | 1.218 (1.067–1.392) | | 0.0044 | | [359] | |  |  |
| SULT1A1 | rs9282861 | c.638G>A | p.Arg213His | Increased | | MA | | 1688 BC(1084) + 2842 N(1539) | | 1.45 (1.18–1.78) | | 0.0004 | | [360] | |  |  |
| TACC3 | rs798766 | c.1591+1211T>C |  | Increased | | Chinese | | 1210 BC() + 1008 N() | | 1.31 (1.09-1.57) | | 0.003 | | [231] | |  |  |
| TACC3 | rs798766 | c.1591+1211T>C |  | Increased | | MA / European | | 4739 BC() + 45549 N() | | 1.24 (1.17–1.32) | | 9.9×10^−12^ | | [361] | |  |  |
| TACC3 | rs798766 | c.1591+1211T>C |  | Increased | | Chinese | | 581 BC() + 1561 N() | | 1.39 (1.15-1.67) | | <0.001 | | [333] | |  |  |
| TACC3 | rs798766 | c.1591+1211T>C |  | Increased | | Swedish | | 4580 BC() + 45269 N() | | 1.24 (1.17–1.32) | | 9.9x10^-12^ | | [73] | |  |  |
| TACC3 | rs798766 | c.1591+1211T>C |  | Increased | | Chinese | | 1050 BC() + 1404 N() | | 1.24 (1.05-1.46) | | 0.012 | | [37] | |  |  |
| TACC3 | rs798766 | c.1591+1211T>C |  | Increased | | Chinese | | 815 BC(230) + 1141 N(253) | | 1.36 (1.10–1.67) | | 0.005 | | [362] | |  |  |
| TACC3 | rs798766 | c.1591+1211T>C |  | Increased | | French | | 231 BC(12) +261 N(30) | | 1.84 (0.9–2.3) | | 0.01 | | [328] | |  |  |
| TACC3 | rs798766 | c.1591+1211T>C |  | Increased | | MA | | BC() + N() | | 1.24 (1.17–1.32) | |  | | [39] | |  |  |
| TERF2 | rs251796 | c.1341-42T>C |  | Increased | | USA / Caucasian | | 803 BC() + 803 N() | | 1.53 (1.08–2.16) | | 0.0154 | | [352] | |  |  |
| TEP1 | rs1760897 | c.346T>C | p.Ser116Pro | Increased | | USA | | 832 BC(75) + 1191 N(75) | | 1.8 (1.2-2.6) | | <0.004 | | [89] | |  |  |
| TEP1 | rs2228041 | c.3464G>C/A | p.Arg632Pro/Gln | Increased | | USA / Caucasian | | 803 BC(69) + 803 N(108) | | 1.66 (1.19–2.31) | | 0.0023 | | [352] | |  |  |
| TEP1 | rs2228026 | c.1719T>C | p.Ile573= | Increased | | USA / Caucasian | | 803 BC(59) + 803 N(94) | | 1.72 (1.20–2.44) | | 0.0025 | | [352] | |  |  |
| TEP1 | rs1713418 | g.20834809A>G |  | Increased | | USA / Caucasian | | 803 BC(535) + 803 N(559) | | 1.42 (1.10–1.83) | | 0.0075 | | [352] | |  |  |
| TEP1 | rs2297615 | c.5508+18T>A |  | Increased | | USA / Caucasian | | 803 BC() + 803 N() | | 1.24 (1.05–1.46) | | 0.0097 | | [352] | |  |  |
| TEP1 | rs2229101 | c.6006T>G | p.Leu2002= | Increased | | USA / Caucasian | | 803 BC() + 803 N() | | 1.46 (1.06–2.00) | | 0.0192 | | [352] | |  |  |
| TEP1 | rs2104978 | c.7685A>G | p.His2562Arg | Increased | | USA / Caucasian | | 803 BC() + 803 N() | | 1.40 (1.03–1.91) | | 0.0309 | | [352] | |  |  |
| TEP1 | rs1713440 | c.568-710A>T |  | Increased | | USA / Caucasian | | 803 BC() + 803 N() | | 1.17 (1.01–1.35) | | 0.0403 | | [352] | |  |  |
| TERT | rs2736098 | c.915G>A | p.Ala305Ala | Increased | | MA | | BC() + N() | | 1.16 (1.08–1.23) | |  | | [39] | |  |  |
| TERT | rs2736098 | c.915G>A | p.Ala305Ala | NA | | MA | | 1050 BC() + 1404 N() | | 1.01 | | 0.858 | | [70] | |  |  |
| TERT | rs2736098 | c.915G>A | p.Ala305Ala | Increased | | India | | 225 BC(42) + 240 N(28) | | 2.78 (1.38-5.59) | | 0.004 | | [117] | |  |  |
| TERT-CLPTM1L | rs401681 | c.1316-153G>A |  | Increased | | MA | | 8829 BC() + 42150 N() | | 1.11 (1.07–1.15) | | <0.001 | | [363] | |  |  |
| THBS1 | rs2664139 | g.39872583T>C/G |  | Increased | | Chinese | | 609 BC(89) + 670 N(75) | | 1.43 (1.01-2.04) | | 0.047 | | [364] | |  |  |
| TNKS | rs7825818 | c.1749+1180T>A |  | Increased | | USA / Caucasian | | 803 BC() + 803 N() | | 1.32 (1.06–1.64) | | 0.0118 | | [352] | |  |  |
| TNKS | rs10503380 | g.9410495C>T |  | Increased | | USA / Caucasian | | 803 BC() + 803 N() | | 1.21 (1.03–1.43) | | 0.0234 | | [352] | |  |  |
| ZNF350 | rs2278415 | c.1503A>T | p.Arg501Ser | Decreased | | Spanish | | 1150 BC() + 1149 N() | | 0.76 (0.62–0.93) | | 0.01 | | [160] | |  |  |
|  | rs12485497 | g.32261987C>A/T |  | Increased | | MA | | 1601 BC() + 1819 N() | |  | |  | | [116] | |  |  |
|  | rs10234749 | g.152387869T>A/C/G |  | Decreased | | Spanish | | 1150 BC(29) + 1149 N(51) | | 0.50 (0.31–0.81) | | 0.01 | | [160] | |  |  |
|  | rs2198843 | g.75001230C>G/T |  | Increased | | Korean | | 269 BC() + 789 N() | | 4.54 (1.0–20.7) | | 0.03 | | [365] | |  |  |
|  | rs35868376 | g.189631841G>A |  | Increased | | MA | | 1601 BC() + 1819 N() | |  | |  | | [116] | |  |  |
|  | rs1014971 | g.39332623C>G/T |  | Increased | | Japanese | | 539 BC(373) + 5581 N(3465) | | 1.19 | | 0.0074 | | [79] | |  |  |
|  | rs1014971 | g.39332623C>G/T |  | Increased | | MA | | BC() + N() | | 0.88 (0.85–0.91) | |  | | [39] | |  |  |
|  | rs4687103 | g.189632664A>G |  | Increased | | MA | | 1601 BC() + 1819 N() | |  | |  | | [116] | |  |  |
|  | rs4687104 | g.189632900A>G |  | Increased | | Turkish | | 250 BC(28) + 250 N(15) | | 2.218 (1.123-4.381) | | 0.02 | | [249] | |  |  |
|  | rs4687104 | g.189632900A>G |  | Increased | | MA | | 1601 BC() + 1819 N() | |  | |  | | [116] | |  |  |
|  | rs34666239 | g.189633513T>A/C |  | Increased | | MA | | 1601 BC() + 1819 N() | |  | |  | | [116] | |  |  |
|  | rs35076301 | g.189633520T>C |  | Decreased | | Chinese | | 314 BC(120) + 204 N(108) | |  | | 0.033 | | [255] | |  |  |
|  | rs35075630 | g.189633862G>A |  | Increased | | MA | | 1601 BC() + 1819 N() | |  | |  | | [116] | |  |  |
|  | rs13089435 | g.189634919G>T |  | Increased | | MA | | 1601 BC() + 1819 N() | |  | |  | | [116] | |  |  |
|  | rs62278269 | g.189636970G>T |  | Increased | | MA | | 1601 BC() + 1819 N() | |  | |  | | [116] | |  |  |
|  | rs35392448 | g.189637003A>G |  | Increased | | MA | | 1601 BC() + 1819 N() | |  | |  | | [116] | |  |  |
|  | rs13059523 | g.189637194G>A |  | Increased | | MA | | 1601 BC() + 1819 N() | |  | |  | | [116] | |  |  |
|  | rs12491886 | g.189637587C>T |  | Increased | | MA | | 1601 BC() + 1819 N() | |  | |  | | [116] | |  |  |
|  | rs4687108 | g.189638487A>G |  | Increased | | MA | | 1601 BC() + 1819 N() | |  | |  | | [116] | |  |  |
|  | rs837775 | g.189643673C>T |  | Increased | | MA | | 1601 BC() + 1819 N() | |  | |  | | [116] | |  |  |
|  | rs56031831 | g.189646516T>C |  | Increased | | MA | | 1601 BC() + 1819 N() | |  | |  | | [116] | |  |  |
|  | rs833052 | g.43723335A>C/G/T |  | Increased | | MA / Asian | | 4359 BC(840) + 5417 N(1148) | | 1.51 (1.11‐2.07) | | 0.009 | | [123] | |  |  |
|  | rs398652 | g.56525569G>A/T |  | Decreased | | USA | | 969 BC(233) + 956 N(271) | | 0.81 (0.67–0.97) | | 0.025 | | [366] | |  |  |
|  | rs1839072 | g.189631640A>G |  | Increased | | MA | | 1601 BC() + 1819 N() | |  | |  | | [116] | |  |  |
|  | rs1447932 | g.189631865A>G |  | Increased | | MA | | 1601 BC() + 1819 N() | |  | |  | | [116] | |  |  |
|  | rs1447933 | g.189631877A>C |  | Increased | | MA | | 1601 BC() + 1819 N() | |  | |  | | [116] | |  |  |
|  | rs4479569 | g.189631932C>G/T |  | Increased | | MA | | 1601 BC() + 1819 N() | |  | |  | | [116] | |  |  |
|  | rs35076301 | g.189633520T>C |  | Increased | | MA | | 1601 BC() + 1819 N() | |  | |  | | [116] | |  |  |
| Not associated | | | | | | | | | | | | | | | |  |  |
| CYP1A1 / CYP1A2 | rs2472297 | g.17998C>T |  | NA | | European | | 3532 BC() + 5120 N() | | 1.03 (0.95-1.11) | | 0.4758 | | [23] | |  |  |
| CYP1A2 | rs2069514 | g.28338G>A |  | NA | | MA | | 2415 BC() + 2208 N() | |  | |  | | [20] | |  |  |
| CYP2D6 | rs3892097 | g.6047G>A |  | NA | | North India | | 100 BC(18) + 76N(14) | | 1.00 (0.46-2.16) | | 0.05 | | [62] | |  |  |
| CYP2D6 | rs3892097 | g.6047G>A |  | NA | | Tunisian | | 80 BC() + 109 N() | |  | | >0.05 | | [269] | |  |  |
| CYP4B1 | rs4646487 | c.517C>T | p.Arg173Trp | NA | | Japanese | | BC(52) + N(55) | | 1.23 (0.80–1.89) | | 0.352 | | [367] | |  |  |
| GSTM3 | rs1332018 | g.110282972G>T |  | NA | | German | | 293 BC() + 176 N() | |  | |  | | [368] | |  |  |
| GSTO1 | rs4925 | c.419C>A | p.Ala140Asp | NA | | Serbian | | 187 BC(18) + 140 N(12) | | 1.3 (0.6–3.1) | | 0.45 | | [56] | |  |  |
| UGT1A4 / UGT1A6 | rs4148328 | c.1307+574C>T |  | NA | | European | | 3532 BC() + 5120 N() | | 0.91 (0.85-0.98) | | 0.0086 | | [23] | |  |  |
| UGT1A8 | rs1105880 | c.856-73715A>G |  | NA | | USA | | 718 BC(426) + 783 N(427) | | 1.18 (1.01-1.38) | | 0.037 | | [77] | |  |  |
| UGT1A8 | rs6759892 | c.856-74011T>G |  | NA | | USA | | 718 BC(485) + 783 N(494) | | 1.17 (1.01-1.36) | | 0.038 | | [77] | |  |  |
| UGT2B7 | rs7439366 | c.802T>C | p.Tyr268His | NA | | German | | 211 BC(58) + 210 N(73) | | 1.36 (0.78–2.34) | |  | | [369] | |  |  |
| LOC105369818 / IL22 | rs1179246 | g.11802T>G |  | NA | | Chinese | | 210 BC(41) + 210 N(47) | | 0.83 (0.49-1.43) | | 0.51 | | [94] | |  |  |
| CD44 | rs1425802 | g.2985T>C |  | NA | | Taiwan | | 275 BC(170) + 275 N(176) | | 0.911 (0.644–1.287) | |  | | [370] | |  |  |
| CD44 | rs713330 | c.1153+611C>A |  | NA | | Taiwan | | 275 BC(44) + 275 N(52) | | 0.817 (0.525–1.270) | |  | | [370] | |  |  |
| CD44 | rs11821102 | g.97128G>A |  | NA | | Taiwan | | 275 BC(41) + 275 N(53) | | 0.734 (0.469–1.148) | |  | | [370] | |  |  |
| CD44 | rs13347 | g.97856C>A/T |  | NA | | Taiwan | | 275 BC(137) + 275 N(132) | | 1.075 (0.770–1.503) | |  | | [370] | |  |  |
| CTLA4 | rs5742909 | g.204732347C>T |  | NA | | India | | 200 BC(5) + 200 N(7) | | 0.48 (0.08–2.82) | | 0.414 | | [82] | |  |  |
| IL13 | rs1800925 | g.3945C>G/T |  | NA | | Chinese | | 817 BC(256) + 1141 N(317) | | 1.18 (0.96–1.43) | |  | | [371] | |  |  |
| IL13 | rs20541 | c.431A>C/G/T | p.Gln144Pro/Arg/Leu | NA | | Chinese | | 817 BC(417) + 1141 N(577) | | 1.01 (0.84-1.21) | |  | | [371] | |  |  |
| IL22 | rs1182844 | g.10853A>T/G/C |  | NA | | Chinese | | 210 BC(43) + 210 N(39) | | 1.19 (0.71-2.00) | | 0.52 | | [94] | |  |  |
| IL31 | rs7977932 | c.166-95G>C |  | NA | | Chinese | | 294 BC(70) + 478 N(93) | | 0.80 (0.57–1.11) | | 0.18 | | [341] | |  |  |
| IL4 | rs2243250 | g.4782C>G/T |  | NA | | Chinese | | 817 BC(303) + 1141 N(439) | | 0.95 (0.78-1.14) | |  | | [371] | |  |  |
| IL4R | rs1801275 | c.1727A>G | p.Gln576Arg | NA | | Chinese | | 817 BC(253) + 1141 N(344) | | 1.04 (0.86-1.27) | |  | | [371] | |  |  |
| IL4R | rs1805010 | c.223A>C/G/T | p.Ile75Leu/Val/Phe | NA | | Chinese | | 817 BC(604) + 1141 N(835) | | 1.03 (0.84-1.26) | |  | | [371] | |  |  |
| IL4R | rs1805015 | c.1507T>C | p.Ser503Pro | NA | | Chinese | | 817 BC(143) + 1141 N(189) | | 1.08 (0.85-1.37) | | 0.852 | | [371] | |  |  |
| IL6 | rs1800796 | g.4481G>A/C |  | NA | | India | | 232 BC(60) + 250 N(60) | | 1.10 (0.73–1.66) | | 0.67 | | [285] | |  |  |
| IL6 | rs1800797 | g.4456A>C/G/T |  | NA | | India | | 232 BC(131) + 250 N(126) | | 1.29 (0.89–1.82) | | 0.2 | | [285] | |  |  |
| TLR3 | rs3775290 | c.1377C>A/T | p.Phe459Leu/= | NA | | North India | | 200 BC(94) + 200 N(78) | | 1.39 (0.93-2.06) | | 0.107 | | [107] | |  |  |
| TLR4 | rs11536865 | g.4564G>C |  | NA | | Chinese | | 436 BC() + 522 N() | |  | |  | | [108] | |  |  |
| TLR4 | rs4986791 | c.1196C>T | p.Thr399Ile | NA | | North India | | 200 BC(37) + 200 N(27) | | 1.45 (0.85–2.49) | | 0.174 | | [107] | |  |  |
| TLR9 | rs352140 | c.1635G>A/C/T | p.Pro545= | NA | | North India | | 200 BC(104) + 200 N(117) | | 0.78 (0.52-1.41) | | 0.191 | | [107] | |  |  |
| HIF1A | rs11549465 | c.1744C>T | p.Pro582Ser | NA | | MA | | 221 BC() + 461 N() | | 1.12 (0.65-1.92) | | 0.923 | | [372] | |  |  |
| HIF1A | rs11549467 | c.1762G>A | p.Ala588Thr | NA | | MA | | 219 BC() + 461N() | | 0.75 (0.40-1.40) | | 0.82 | | [372] | |  |  |
| VDR | rs731236 | c.1056T>C | p.Ile352= | NA | | North India | | 130 BC(50) + 346 N(138) | | 1.377 (0.639–2.968) | | 0.477 | | [113] | |  |  |
| IGFBP3 | rs2854744 | g.4797C>G/A |  | NA | | MA | | 1450 BC() + 1725 N() | | 1.11 (0.91-1.37) | | 0.4446 | | [373] | |  |  |
| NGF | rs12760036 | c.-136-12079T>G |  | NA | | European | | 3532 BC() + 5120 N() | | 0.85 (0.76-0.96) | | 0.0096 | | [23] | |  |  |
| OSMR | rs2278329 | c.1657G>A |  | NA | | Chinese | | 306 BC(405) + 459 N(614) | | 0.97 (0.78–1.20) | | 0.77 | | [374] | |  |  |
| TGFB1 | rs1800471 | c.74G>C/A | p.Arg25Pro/Gln | NA | | India | | 237 BC(19) + 301 N(34) | | 0.67 (0.37–1.21) | | 0.18 | | [105] | |  |  |
| TGFB1 | rs1800471 | c.74G>C/A | p.Arg25Pro/Gln | NA | | Spanish | | 1157 BC(129) + 1157 N(142) | | 0.80 (0.61–1.05) | | 0.081 | | [286] | |  |  |
| TGFB1 | rs1800472 | c.788C>T | p.Thr263Ile | NA | | Spanish | | 1157 BC(81) + 1157 N(71) | | 1.1 (0.77–1.55) | |  | | [286] | |  |  |
| TGFB1 | rs13447341 | c.140A>G | p.Glu47Gly | NA | | India | | 237 BC(17) + 301 N(27) | | 0.77 (0.41–1.45) | | 0.416 | | [105] | |  |  |
| TGFBR1 | rs11466445 | c.52_54GCG[4]/[13] | p.Ala22_Ala26del | NA | | Spanish | | 1157 BC(199) + 1157 N(191) | | 0.99 (0.79–1.25) | | 0.704 | | [286] | |  |  |
| TGFBR1 | rs334358 | c.1386+547G>T |  | NA | | Spanish | | 1157 BC(359) + 1157 N(327) | | 1.07 (0.88–1.29) | | 0.682 | | [286] | |  |  |
| TGFBR1 | rs868 | g.49245A>G |  | NA | | Spanish | | 1157 BC(351) + 1157 N(316) | | 1.07 (0.88–1.30) | | 0.475 | | [286] | |  |  |
| TGFBR1 | rs928180 | c.575-2409A>C |  | NA | | Spanish | | 1157 BC(175) + 1157 N(179) | | 0.96 (0.75–1.22) | | 0.779 | | [286] | |  |  |
| TNF-α | rs1800629 | g.4682G>A |  | NA | | MA | | 1311 BC() + 1436 N() | | 1.01 (0.83–1.23) | | 0.94 | | [270] | |  |  |
| TNF-α | rs1800629 | g.4682G>A |  | NA | | MA | | 1142 BC() + 1228 N() | | 1.01 (0.75,1.35) | | 0.955 | | [271] | |  |  |
| VEGFA | rs833061 | g.4534C>G/T |  | NA | | MA | | 1717 BC() + 2451 N() | | 1.03 (0.88,1.21) | | 0.718 | | [122] | |  |  |
| VEGFA | rs35569394 | ‐2549I/D | 18-bp insertion/deletion | NA | | Tunisian | | 218 BC(144) + 204 N(143) | | 0.83 (0.55‐1.25) | | 0.374 | | [125] | |  |  |
| ATG16L1 | rs2241880 | c.898A>G | p.Thr300Ala | NA | | Turkish | | 69 BC(62) + 156 N(150) | | 1.135 (0.759-1.697) | | 0.537 | | [375] | |  |  |
| DR4 | rs4871857 | c.626G>T/C | p.Arg209Thr | NA | | North India | | 200 BC(114) + 225 N(128) | | 0.87 (0.55-1.38) | | 0.568 | | [138] | |  |  |
| DR4 | rs20576 | c.683A>C | p.Ala228Glu | NA | | North India | | 200 BC(127) + 225 N(134) | | 1.32 (0.83-2.09) | | 0.247 | | [138] | |  |  |
| XRCC1 | rs1799780 | c.602-33C>T |  | NA | | Caucasian | | 547 BC(56) + 579 N(47) | | 1.33 (0.88–2.01) | | 0.18 | | [290] | |  |  |
| XRCC1 | rs2307187 | g.5120C>T |  | NA | | Caucasian | | 547 BC(13) + 579 N(16) | | 0.91 (0.43–1.92) | | 0.8 | | [290] | |  |  |
| XRCC1 | rs25496 | c.215T>C | p.Val72Ala | NA | | Caucasian | | 547 BC(0) + 579 N(0) | |  | |  | | [290] | |  |  |
| XRCC1 | rs2682558 | g.37415G>A |  | NA | | Caucasian | | 547 BC(142) + 579 N(162) | | 0.91 (0.69–1.20) | | 0.48 | | [290] | |  |  |
| XRCC1 | rs2682585 | g.3443T>G/C/A |  | NA | | Caucasian | | 547 BC(22) + 579 N(21) | | 1.15 (0.61–2.18) | | 0.66 | | [290] | |  |  |
| XRCC1 | rs2682586 | g.3232T>G/C/A |  | NA | | Caucasian | | 547 BC(181) + 579 N(187) | | 1.07 (0.82–1.39) | | 0.62 | | [290] | |  |  |
| XRCC1 | rs3213239 | g.3668_3671dup |  | NA | | Caucasian | | 547 BC(80) + 579 N(91) | | 1.02 (0.71–1.48) | | 0.91 | | [290] | |  |  |
| XRCC1 | rs3213245 | g.5044C>T/A |  | NA | | Caucasian | | 547 BC(266) + 579 N(275) | | 1.00 (0.76–1.31) | | 0.97 | | [290] | |  |  |
| XRCC1 | rs3213401 | g.37413_37419del |  | NA | | Caucasian | | 547 BC(59) + 579 N(58) | | 1.15 (0.78–1.70) | | 0.48 | | [290] | |  |  |
| XRCC1 | rs3547 | c.1896A>T/G | p.Gln632His/= | NA | | Caucasian | | 547 BC(268) + 579 N(275) | | 0.94 (0.72–1.24) | | 0.67 | | [290] | |  |  |
| XRCC3 | rs1799796 | c.562-14A>T |  | NA | | India | | 211 BC(6) + 244 N(7) | | 1.30 (0.36–3.43) | | 0.831 | | [161] | |  |  |
| XRCC4 | rs28360071 | c.315+31120_315+31149del |  | NA | | India | | 211 BC(47) + 244 N(50) | | 1.08 (0.65–1.80) | | 0.754 | | [161] | |  |  |
| XRCC5 | rs11685387 | g.4795C>T |  | NA | | Taiwanese | | 288 BC(79) + 288 N(68) | |  | | 0.4983 | | [163] | |  |  |
| XRCC5 | rs9288518 | c.2109+2011A>G |  | NA | | Taiwanese | | 288 BC(87) + 288 N(98) | |  | | 0.3375 | | [163] | |  |  |
| ERCC6 | rs2228529 | c.4238A>G | p.Gln1413ARG | NA | | Taiwanese | | 288 BC(37) + 288 N(45) | | 0.8 (0.50-1.27) | | <0.05 | | [188] | |  |  |
| ERCC6 | rs4253211 | c.3689G>C | p.Arg1230Pro | NA | | MA | | 1851 BC() + 1900 N() | | 0.9 (0.77-1.05) | | 0.567 | | [272] | |  |  |
| RAD51 | rs1801320 | g.5170G>C |  | NA | | India | | 270 BC(111) +251 N(118) | | 0.79 (0.55–1.14) | | 0.188 | | [180] | |  |  |
| MGMT | rs1803965 | c.159C>T | p.Leu53= | NA | | Southern China | | 167 BC(38) + 204 N(31) | | 1.60 (0.94–2.73) | | 0.063 | | [376] | |  |  |
| MGMT | rs12917 | c.250C>T | p.Leu84Phe | NA | | Southern China | | 167 BC(35) + 204 N(31) | | 1.40 (0.82–2.41) | | 0.149 | | [376] | |  |  |
| RAP1B | rs11177325 | g.69064978C>T |  | NA | | European | | 3532 BC() + 5120 N() | | 0.95 (0.89-1.02) | | 0.1951 | | [23] | |  |  |
| BIRC5 | rs1042489 | g.14930T>C/G |  | NA | | MA | | 6468 BC() + 7983 N() | | 1.12 (0.88–1.42) | | 0.365 | | [216] | |  |  |
| SOD2 | rs4880 | c.47T>C | p.Val16Ala | NA | | MA | | 1367 BC () + 1792 N () | | 1.038 (0.782–1.377) | | 0.015 | | [273] | |  |  |
| AP1B1 | rs5763140 | g.29718321C>T |  | NA | | European | | 3532 BC() + 5120 N() | | 1.08 (0.97-1.19) | | 0.15 | | [23] | |  |  |
| AP2A2 | rs7483870 | c.474-1076G>A |  | NA | | European | | 3532 BC() + 5120 N() | | 0.96 (0.89-1.04) | | 0.3014 | | [23] | |  |  |
| ARF1 | rs3768331 | c.-37-2084C>T |  | NA | | European | | 3532 BC() + 5120 N() | | 1.05 (0.98-1.12) | | 0.1536 | | [23] | |  |  |
| TMEM107 | rs3202848 | g.8075G>A |  | NA | | European | | 3532 BC() + 5120 N() | | 0.93 (0.86-1.00) | | 0.0572 | | [23] | |  |  |
| MMP11 | rs738791 | c.108+2360C>T |  | NA | | Taiwan | | 431 BC(250) + 650 N(341) | | 1.222 (0.946–1.579) | | <0.05 | | [248] | |  |  |
| MMP11 | rs2267029 | c.108+2751A>G |  | NA | | Taiwan | | 431 BC(201) + 650 N(285) | | 1.107 (0.858–1.428) | | <0.05 | | [248] | |  |  |
| MMP11 | rs738792 | c.113C>T | p.Ala38Val | NA | | Taiwan | | 431 BC(224) + 650 N(309) | | 1.160 (0.900–1.495) | | <0.05 | | [248] | |  |  |
| MMP11 | rs131451 | g.3509C>A/T |  | NA | | Taiwan | | 431 BC(292) + 650 N(417) | | 1.121 (0.857–1.465) | | <0.05 | | [248] | |  |  |
| MMP12 | rs2276109 | g.4974A>G |  | NA | | Polish | | 241 BC(67) + 199 N(51) | | 1.21 (0.78–1.88) | | 0.372 | | [243] | |  |  |
| MMP7 | rs11568819 | g.102401633G>A |  | NA | | Taiwan | | 375 BC(0) + 375 N(0) | |  | |  | | [247] | |  |  |
| MMP8 | rs11225395 | g.4206T>G/C |  | NA | | MA | | 8140 BC(4372) + 10 529 N(5066) | | 0.98 (0.92–1.04) | | 0.429 | | [274] | |  |  |
| MMP8 | rs35866072 | c.1379A>C | p.Lys460Thr | NA | | MA | | 8140 BC(3019) + 10 529 N(3544) | | 0.94 (0.67–1.32) | | 0.729 | | [274] | |  |  |
| MMP8 | rs1940475 | c.259A>G | p.Lys87Glu | NA | | MA | | 8140 BC(749) + 10 529 N(1919) | | 1.05 (0.93–1.18) | | 0.43 | | [274] | |  |  |
| MMP9 | rs3918242 | g.3430C>T |  | NA | | Polish | | 241 BC(91) + 199 N(65) | | 1.30 (0.86–1.95) | | 0.209 | | [243] | |  |  |
| MMP9 | rs3918242 | g.3430C>T |  | NA | | MA | | 839 BC() + 775 N() | | 1 (0.55-1.80) | | 0.998 | | [244] | |  |  |
| MMP9 | rs3918242 | g.3430C>T |  | NA | | Turkish | | 100 BC() + 100 N() | |  | |  | | [275] | |  |  |
| MMP9 | rs3918241 | g.3189T>A |  | NA | | MA | | 6154 BC() + 6330 N() | |  | |  | | [276] | |  |  |
| MMP9 | rs2250889 | c.1721G>A/C/T | p.Arg574Gln/Pro/Leu | NA | | MA | | 6154 BC() + 6330 N() | |  | |  | | [276] | |  |  |
| MMP9 | rs17576 | c.836A>C/G/T | p.Gln279Pro/Arg/Leu | NA | | MA | | 6154 BC() + 6330 N() | |  | |  | | [276] | |  |  |
| MMP9 | rs17577 | c.2003G>A/C | p.Arg668Gln/Pro | NA | | MA | | 6154 BC() + 6330 N() | |  | |  | | [276] | |  |  |
| TIMP1 | rs2070584 | g.9830T>C/G |  | NA | | Polish | | 241 BC(50) + 199 N(21) | | 0.66 (0.11–3.67) | | 0.638 | | [243] | |  |  |
| TIMP2 | rs8179090 | g.76921889C>G |  | NA | | Turkish | | 100 BC() + 100 N() | |  | |  | | [275] | |  |  |
| TIMP3 | rs9619311 | g.4892T>C |  | NA | | Polish | | 241 BC(111) + 199 N(95) | | 0.99 (0.67–1.45) | | 0.952 | | [243] | |  |  |
| MnSOD | rs4880 | c.47T>C | p.Val16Ala | NA | | Turkish | | 157 BC(105) + 224 N(135) | | 1.14 (0.90–1.43) | | 0.262 | | [314] | |  |  |
| MTHFR | rs2274976 | c.1781G>A | p.Arg594Gln | NA | | Iranian | | 158 BC(13) +316 N(27) | | 0.93 (0.69–1.51) | | 0.87 | | [259] | |  |  |
| MTRR | rs1532268 | c.524C>T | p.Ser175Leu | NA | | Tunisian | | 185 BC(141) + 191 N(136) | | 1.3 (0.80-2.11) | | 0.32 | | [267] | |  |  |
| NAMPT | rs9034 | g.105890056A>C/G/T |  | NA | | Chinese | | 407 BC(52) + 316 N(44) | | 1.10 (0.72–1.70) | | 0.65 | | [268] | |  |  |
| NMRK1 | rs7021664 | c.496+886A>T |  | NA | | European | | 3532 BC() + 5120 N() | | 0.94 (0.83-1.06) | | 0.3193 | | [23] | |  |  |
| NOS3 | rs2070744 | c.-51-762C>G |  | NA | | Turkish | | 75 BC(62) + 143 N(102) | | 0.787 (0.525-1.180) | | 0.246 | | [316] | |  |  |
| STX4 | rs10871454 | c.145-1168C>T |  | NA | | European | | 3532 BC() + 5120 N() | | 1 (0.94-1.07) | | 0.9722 | | [23] | |  |  |
| VAMP5 | rs719023 | c.4-360T>C |  | NA | | European | | 3532 BC() + 5120 N() | | 0.94 (0.88-1.00) | | 0.0631 | | [23] | |  |  |
| CENPA | rs2060390 | g.27024478G>A/C |  | NA | | European | | 3532 BC() + 5120 N() | | 0.98 (0.91-1.06) | | 0.6106 | | [23] | |  |  |
| CENPQ | rs4267943 | c.187G>A | p.Gly63Arg | NA | | European | | 3532 BC() + 5120 N() | | 0.94 (0.87-1.01) | | 0.0706 | | [23] | |  |  |
| MIR499A / MIR499B | rs3746444 | g.33578251A>C/G |  | NA | | India | | 212 BC(117) + 250 N(129) | | 1.09 (0.75-1.59) | | 0.622 | | [348] | |  |  |
| NDC80 | rs13381300 | c.-10+413T>G |  | NA | | European | | 3532 BC() + 5120 N() | | 0.91 (0.8-1.04) | | 0.1673 | | [23] | |  |  |
| NT5C3B | rs9907244 | g.39980900T>A/C/G |  | NA | | European | | 3532 BC() + 5120 N() | | 0.95 (0.89-1.01) | | 0.1094 | | [23] | |  |  |
| NTRK1 | rs1888861 | g.17420C>A/G/T |  | NA | | European | | 3532 BC() + 5120 N() | | 0.95 (0.88-1.03) | | 0.2275 | | [23] | |  |  |
| RIBC1 | rs1264013 | c.942C>A/T | p.Thr314= | NA | | European | | 3532 BC() + 5120 N() | | 1 (0.95-1.05) | | 0.9876 | | [23] | |  |  |
| SFRP1 | rs921142 | g.41169276T>A/C |  | NA | | German / Caucasian | | 403 BC(128) + 332 N(165) | |  | | 0.432 | | [358] | |  |  |
| SGF29 | rs4788073 | c.76-1685A>G |  | NA | | European | | 3532 BC() + 5120 N() | | 0.99 (0.93-1.06) | | 0.8344 | | [23] | |  |  |
| SULT1A1 | rs1968752 | g.8282A>T/C |  | NA | | European | | 3532 BC() + 5120 N() | | 1.01 (0.95-1.08) | | 0.7321 | | [23] | |  |  |
| TARS1 | rs2292016 | g.4901G>T |  | NA | | Chinese | | 306 BC(407) + 459 N(591) | | 1.20 (0.84–1.70) | | 0.39 | | [374] | |  |  |
| ZNF106 | rs4924682 | g.42771300T>C |  | NA | | European | | 3532 BC() + 5120 N() | | 1.27 (0.95-1.70) | | 0.1087 | | [23] | |  |  |

Table 2: Summary of all genes that were taken into consideration in this study. NA = Not associated, MA= meta-analysis, MA* = meta-analysis with an association in the Asian race, MA**= meta-analysis with an association in the Caucasian race, MA***= meta-analysis with an association in the Chinese population. For the population the size of the sample of bladder cancer or normal people was reported as a number before BC (bladder cancer) or N (normal) and number between brackets represents the number of people in the sample that have the mutation of interest
